# Metal Ion Signaling in Biomedicine

**DOI:** 10.1021/acs.chemrev.4c00577

**Published:** 2025-01-02

**Authors:** Raphaël Rodriguez, Sebastian Müller, Ludovic Colombeau, Stéphanie Solier, Fabien Sindikubwabo, Tatiana Cañeque

**Affiliations:** † Institut Curie, CNRS, INSERM, PSL Research University, 75005 Paris, France; ‡ Université Paris-Saclay, UVSQ, 78180 Montigny-le-Bretonneux, France

## Abstract

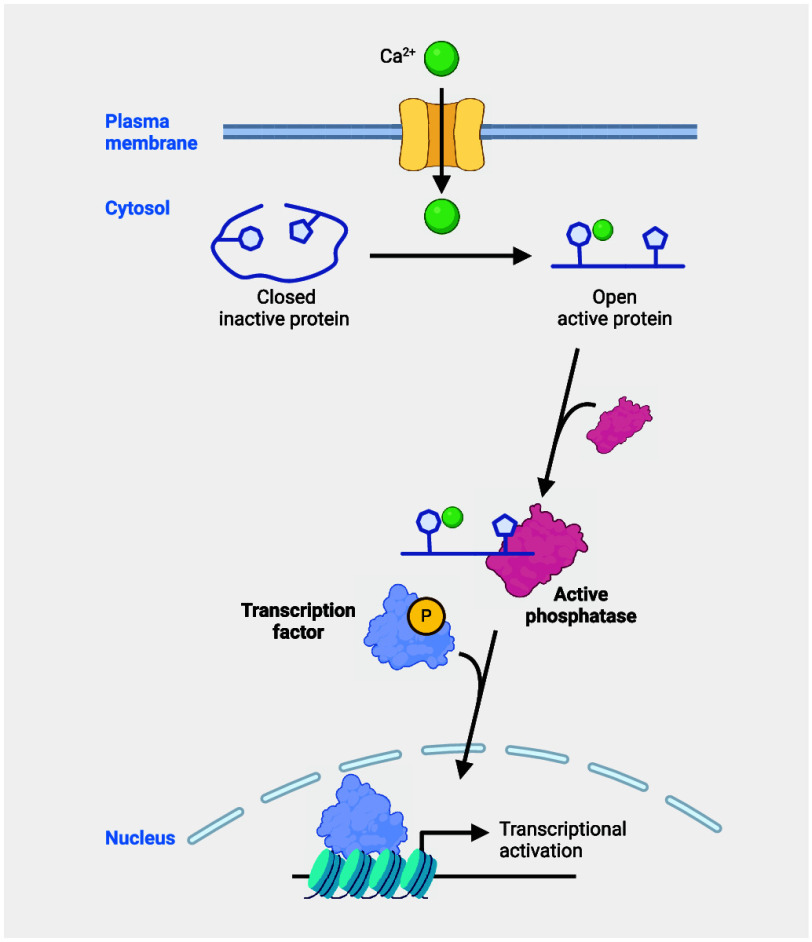

Complex multicellular organisms are composed of distinct tissues involving specialized cells that can perform specific functions, making such life forms possible. Species are defined by their genomes, and differences between individuals within a given species directly result from variations in their genetic codes. While genetic alterations can give rise to disease-causing acquisitions of distinct cell identities, it is now well-established that biochemical imbalances within a cell can also lead to cellular dysfunction and diseases. Specifically, nongenetic chemical events orchestrate cell metabolism and transcriptional programs that govern functional cell identity. Thus, imbalances in cell signaling, which broadly defines the conversion of extracellular signals into intracellular biochemical changes, can also contribute to the acquisition of diseased cell states. Metal ions exhibit unique chemical properties that can be exploited by the cell. For instance, metal ions maintain the ionic balance within the cell, coordinate amino acid residues or nucleobases altering folding and function of biomolecules, or directly catalyze specific chemical reactions. Thus, metals are essential cell signaling effectors in normal physiology and disease. Deciphering metal ion signaling is a challenging endeavor that can illuminate pathways to be targeted for therapeutic intervention. Here, we review key cellular processes where metal ions play essential roles and describe how targeting metal ion signaling pathways has been instrumental to dissecting the biochemistry of the cell and how this has led to the development of effective therapeutic strategies.

## 1 Introduction

**Figure 1:**
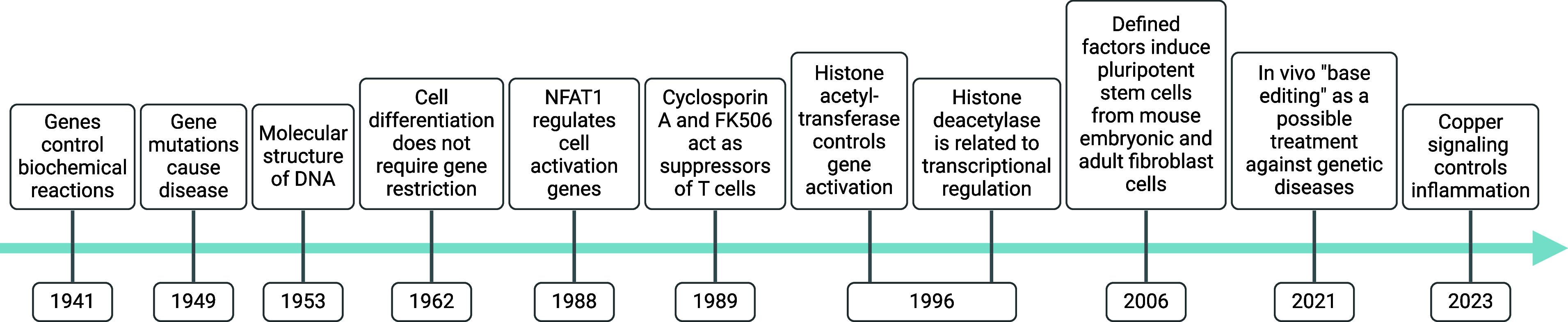
Genetic versus nongenetic control of cell identity and the rise of metal ion signaling. Timeline of discoveries describing how genetic and nongenetic chemical events can control cell identity, illustrating the central roles of metal ions. Figure generated with BioRender.com.

In 1941, Beadle and Tatum reported the discovery that genes control biochemical events in the cell.^1^ Shortly thereafter, Pauling and co-workers elucidated the molecular basis of sickle cell anemia^2^ (Figure 1). Back then, it was anticipated that mutations in genomic DNA could lead to disease-causing dysfunctional gene products from which emerged the concept of “*molecular disease*” and the science of “*molecular medicine*” (Figure 1). These discoveries were made without prior knowledge of the double helical structure of DNA, which was reported only a few years later by Franklin, Crick, Watson, and others^3,4^ (Figure 1). Seventy years later, molecular editing of this genetic defect using CRISPR/Cas technology corrected sickle cell anemia in vivo^5−8^ (Figure 1). In 1962, Gurdon demonstrated that terminally differentiated cell nuclei can give rise to healthy organisms using nuclear transplantation, which illustrated that cell differentiation, acquisition of a specific phenotype by a cell, does not necessarily involve gene restriction^9^ (Figure 1). Almost half a century later, Yamanaka identified the key transcription factors (TFs) essential to produce pluripotent stem cells from adult fibroblasts^10^ (Figure 1). These studies represent a real conceptual rupture, illuminating that cells can adopt different states or identities independently of genetic alterations, a biological process broadly termed cell plasticity or cell-state transitioning.

This challenges the view that cancer cells acquire resistance to therapy exclusively through genetic mutations. Indeed, cancer cells can exploit mechanisms similar to that found in development,^11^ allowing them to shift between highly proliferative to less proliferative yet more invasive states, to become refractory to current standard-of-care therapies, including antiproliferative drugs, and to promote cancer metastases.^12^ While gene mutations contribute to tumorigenesis, the capacity of a cell to adapt quickly to its environment and to adopt distinct states independently of genetic alterations drives drug tolerance. Similarly, immune cells can rapidly respond to the presence of pathogens and activate the appropriate clearance mechanisms by adopting distinct phenotypes, for instance, that of inflammatory macrophages.^13^ The acquisition of distinct cell states marks many biological and pathophysiological processes other than cancer, such as wound healing and inflammation. To this end, cells have evolved molecular mechanisms allowing rapid and reversible adaptation, which involves the capacity of a cell to convert extracellular physical, chemical, or biochemical signals into metabolic and/or transcriptional changes. In 1996, Allis and Schreiber independently reported the discovery of histone acetyltransferases (HATs) and histone deacetylases (HDACs), respectively, two distinct classes of enzymes, which through chemical modification of histone proteins impact DNA-related processes including gene expression^14,15^ (Figure 1). These findings led to the concept that chromatin acts as a signaling platform rather than a mere structural element required for DNA compaction, and that different chromatin states shape transcriptional profiles and cell identity.^16,17^ Understanding the molecular basis underlying the control of cell states is of paramount importance, as this enables the development of therapeutic strategies to correct potential biochemical imbalances. Cell signaling encompasses the entire biochemical chain of events transducing external cellular stimuli to metabolic and transcriptional changes, enabling cells to acquire distinct physical, chemical, and biological properties.

**Figure 2:**
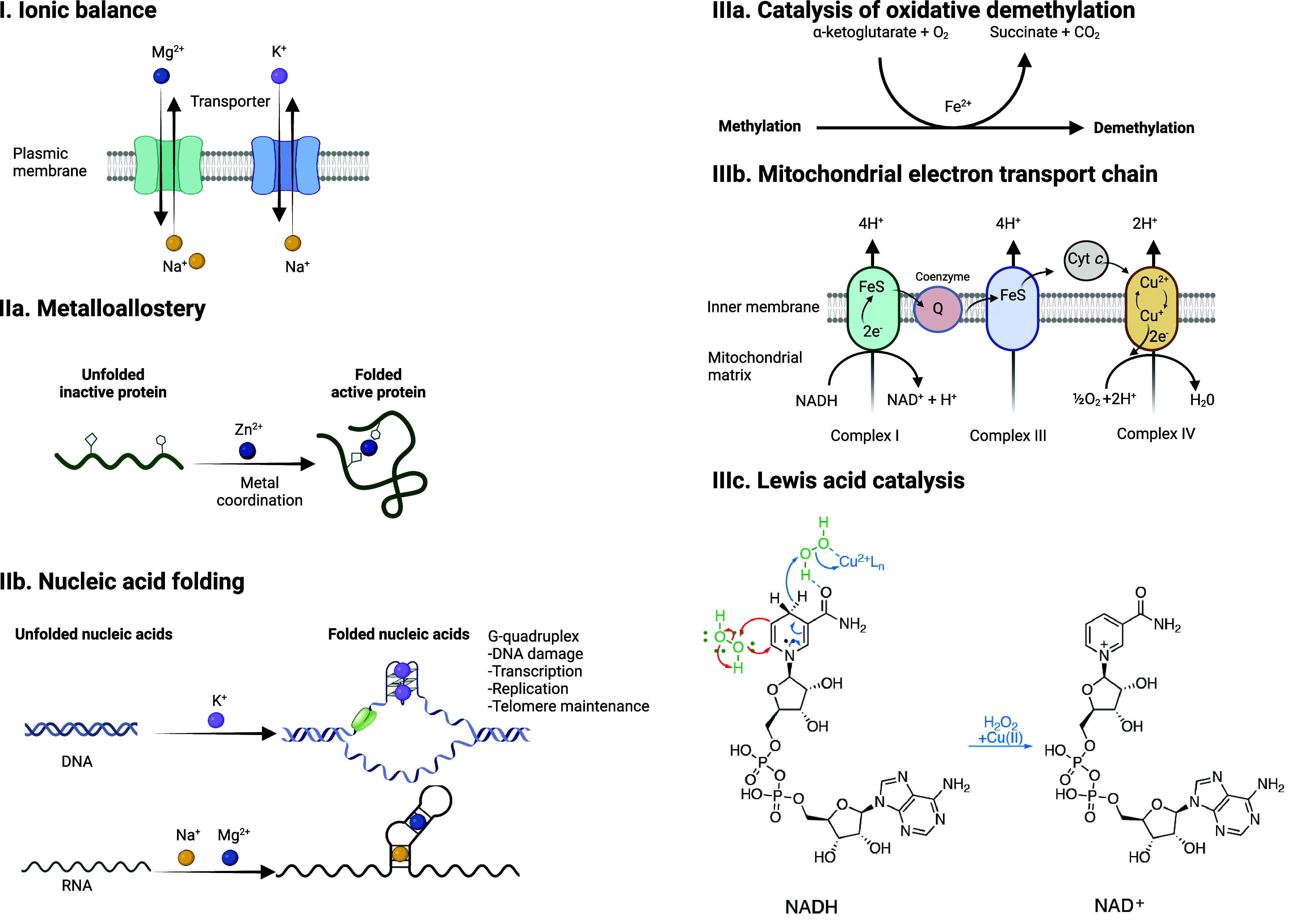
Central roles of metal ions in the biology of the cell. (I) Metal ions act as counterions to maintain ionic balance. (II) Metal ions control protein and nucleic acid folding and multimerization, promoting or inhibiting function. (III) Metal ions promote chemical reactions regulating cell metabolism and the epigenetic control of transcriptional programs. See Abbreviations. Figure generated with BioRender.com.

**Figure 3:**
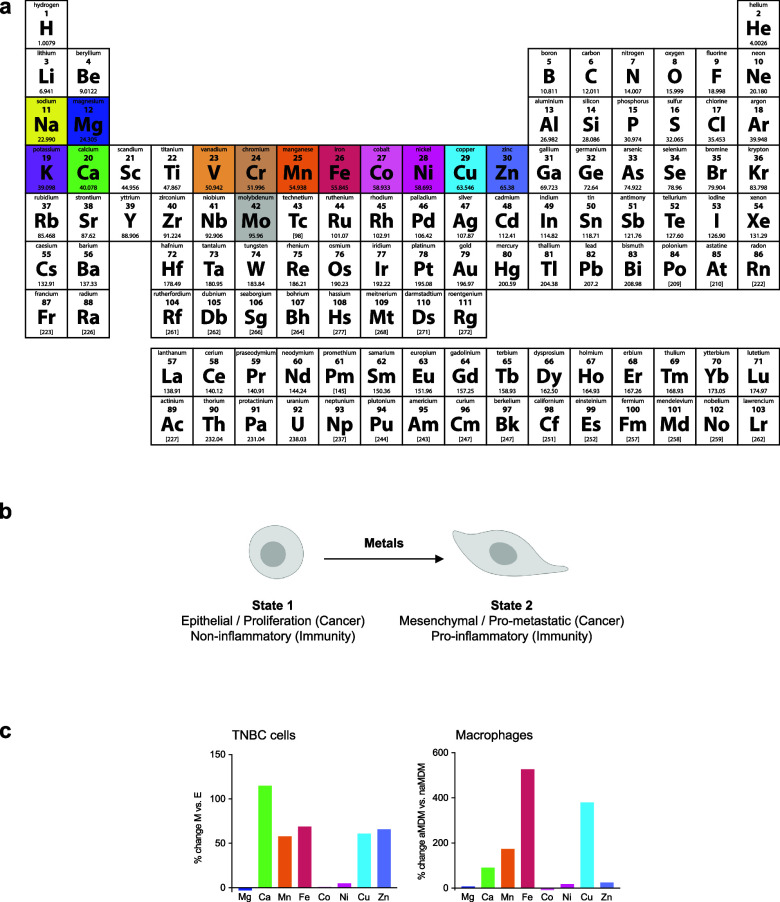
Metals in human biology. (a) Periodic table highlighting metals involved in cell signaling. (b) Schematic illustration of the role of metals in the control of cell identity. (c) ICP-MS quantification of cellular metal contents showing increased levels of specific metals in human triple-negative breast cancer (TNBC) MDA-MB-468 cells comparing mesenchymal (M) versus epithelial (E) cell states and in human monocyte-derived macrophages comparing inflammatory (aMDM) versus noninflammatory (naMDM) cell states. Data reported by the authors.^13^ See Abbreviations. Figure generated with BioRender.com.

Often considered to be trace elements, metals play central roles in cell signaling by balancing negative charges, by enabling cation exchange/transport (e.g., proton, sodium, potassium), as inducers of protein and nucleic acid folding, promoting or inactivating functions by metalloallostery, or as catalysts of chemical reactions (e.g., iron, copper) (Figure 2). Here, we broadly define the action of metal ions in promoting signal transduction within a cell as “*metal ion signaling*”. The capacity of a given metal to promote specific biochemical processes within a cell is inherently linked to its position in the periodic table, that is, its intrinsic electron configuration (Figure 3a). Early on, Tsien recognized the critical role of calcium in developmental biology, identifying rapid increase of intracellular calcium levels in *Xenopus laevis* embryos upon T cell activation and during the cell cycle.^18−21^ It was in the late 1980s that Schreiber and Crabtree truly pioneered the field of cell signaling with the discovery of the first membrane-to-nucleus cell-signaling pathway involving metal ions and showing that calcium promotes specific transcriptional programs in T cells^22−26^ (Figure 1). Metals can readily coordinate protein and nucleic acid residues, inducing conformational changes, thereby enabling activity. Other metals susceptible to readily accept (e.g., redox activity, Lewis acid catalysis) or release electrons can, on the contrary, directly catalyze specific chemical reactions, defining these metals as “*enabling catalysts*” as opposed to “*facilitating cofactors*”.^27^ For example, copper can catalyze the activation of hydrogen peroxide and oxidation of nicotinamide adenine dinucleotide hydride (NADH), which promotes metabolic and epigenetic programming of inflammatory macrophages^13^ (Figure 1). Notably, it was shown that the content of specific metals varies in immune and cancer cells undergoing cell-state transitions and that some of these metals orchestrate the acquisition of a distinct cell identity^13^ (Figure 3b,c).

The field of bioinorganic cell biology has gained momentum with the development of new techniques to investigate metal contents of a cell, their subcellular localizations, and oxidation states. These include organelle-selective and oxidation state-specific reporters coupled to high-resolution fluorescence microscopy,^28−32^ quantitative inductively coupled plasma-mass spectrometry (ICP-MS)^33^ and nanoscale secondary ion mass spectrometry-based imaging.^34^ Cell signaling can go awry and cause disease. Thus, understanding the intricacies of cellular metal ion homeostasis is critical for the development of drugs to rebalance these processes. Biologically active small molecules have been instrumental in dissecting and manipulating the biochemistry of the cell, providing the means to identify druggable targets and to develop new medicines.^35,36^ While TFs, kinases, and other classes of proteins such as chromatin readers have long been thought to be undruggable by small molecules, phenotype-based drug target identification together with structure-based rational design have proven to be powerful approaches to identify previously uncharted targets and to elucidate mechanisms of action (MoA) and cell signaling cascades.^37−43^ While manipulating metal ion homeostasis with chelators and ionophores can confer therapeutic benefits^44^ in diseases characterized by a toxic metal ion imbalance, including myelodysplastic syndrome, β-thalassemia, Menkes disease (MD), and Wilson’s disease (WD), fine-tuning metal ion signaling is until now less common and the rational design of metal targeting compounds remains a challenging endeavor. Nevertheless, one may adopt the optimistic view previously crafted by the Nobel Peace Prize laureate Nelson Mandela and adapted here in the context of metal ion signaling: “*It is undruggable until someone drugs it*”.

In this review, we discuss the roles of metal ions in the biology of the cell and document how cells take up, distribute, store, and export metals. Then, we provide details as to how metals are exploited for their charge balance effects, information storage (supramolecular properties),^45^ and chemical reactivity within the cell (Figure 2). Then we detail how these properties fuel signal transduction within the cell. Finally, we describe examples of metal ion signaling networks that have been targeted with therapeutic value, exploiting metals as direct targets or alternatively drugging other biomolecules upstream or downstream of key metal ion signaling cascades. While we do not provide an exhaustive list of biologically active small molecules targeting metal ion signaling, we have listed key examples of biologically active chemical substances impacting metal ion signaling in Tables 1–13, illustrating the power of chemistry to dissect and manipulate biological processes for basic research purposes and biomedicine.

## 2 Alkali Metal Ion Signaling

### 2.1 Regulation of Sodium and Potassium Homeostasis

**Figure 4:**
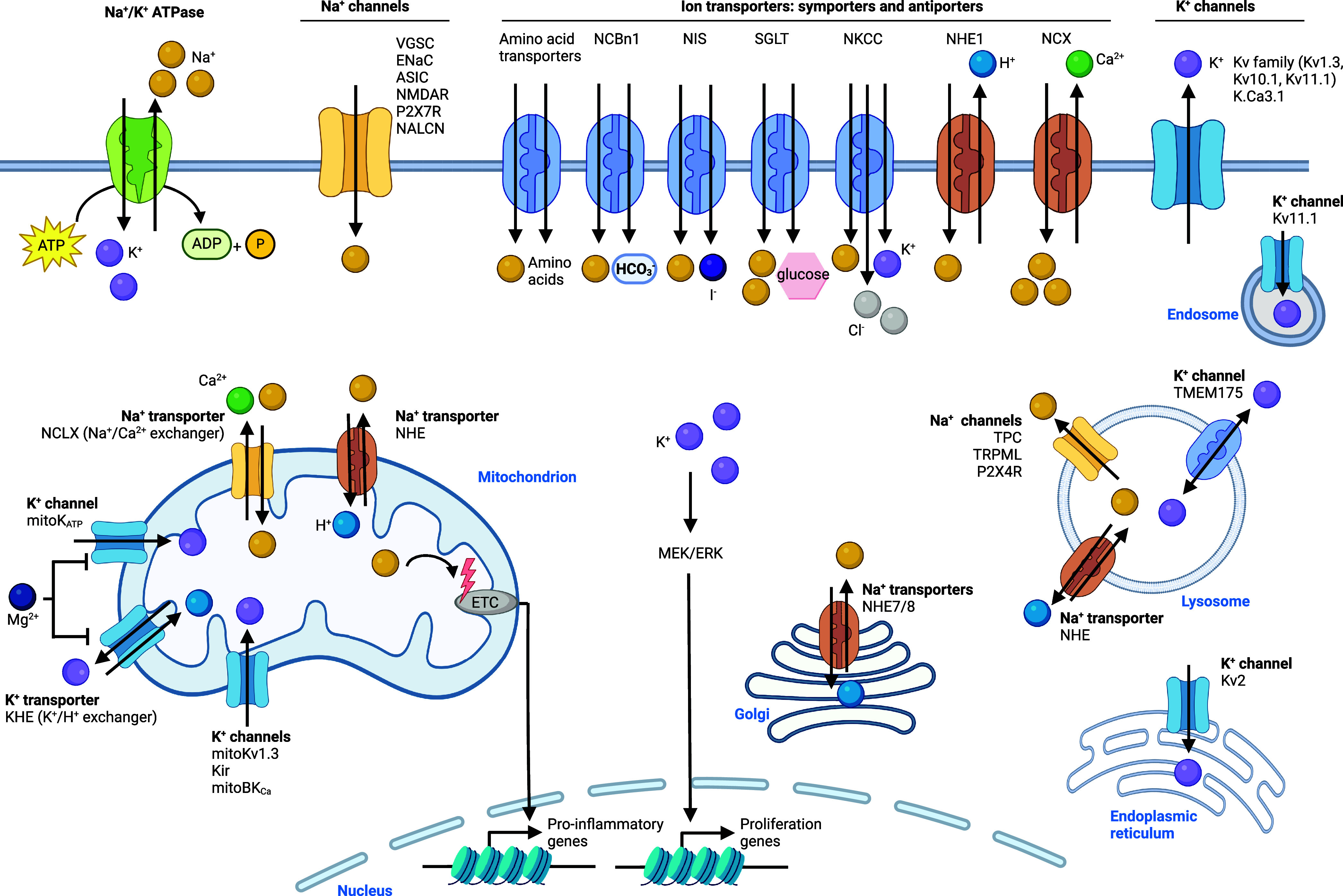
Sodium and potassium signaling. Sodium is taken up into the cell via specific ion channels and transporters. Intracellular sodium is trafficking to mitochondria, the Golgi apparatus, and lysosomes. High sodium levels impact the ETC leading to expression of inflammatory genes in immune cells. Sodium is exported outside of the cell by sodium/potassium ATPase. Potassium is taken up into the cell via specific transporters, such as NKCC and sodium/potassium ATPase. Intracellular potassium is trafficking to lysosomes, mitochondria, and the ER. Potassium activates the MEK/ERK pathway, resulting in the transcription of genes involved in cell proliferation. Potassium is exported outside of the cell by the potassium voltage-gated channel (Kv) and calcium-activated potassium channel (K.Ca3.1). See Abbreviations. Figure generated with BioRender.com.

Sodium and potassium are abundant in cellular systems. Both alkali metals are closely linked in their cellular regulation and function. For instance, some transporters are coupled antiporters or symporters of both metals as depicted Figure 4. Sodium is imported into cells through some cellular metal ion transporters coupled to various ion channels, including the voltage-gated sodium channel (VGSC), the epithelial sodium channel (ENaC), the acid-sensing ion channel (ASIC), the *N*-methyl-d-aspartate receptor (NMDAR), the purinergic P2X receptor 7 (P2X7R), and the sodium leak channel nonselective (NALCN), which are differentially expressed in distinct tissues^46,47^ and of which ENaC is the most well-characterized to date^48^ (Figure 4). Ion channels are membrane proteins that form pores and allow ions to pass through. Sodium can also be taken up by transporters, which are proteins that pump ions via a membrane, often using energy, for instance, from the hydrolysis of adenosine triphosphate (ATP). Symporters transport different types of ions in the same direction, whereas antiporters transport different types of ions in opposite directions over a lipid membrane. Various antiporters have been documented, which include the sodium/calcium exchanger NCX and sodium/proton exchanger NHE1. Various symporters that import sodium have also been described, including amino acid symporters, the sodium/bicarbonate symporter NCBn1, NKCC, which imports sodium, potassium, and chloride, the sodium/glucose symporter SGLT, and the sodium/iodide symporter NIS.^49,50^ Other members of the NHE family are involved in the transport of sodium across membranes of different cell organelles, including NHE7 and NHE8 in the membrane of the Golgi apparatus and NHE in the membrane of lysosomes. NHE is also situated in the membrane of mitochondria, and mitochondrial sodium/calcium exchanger NCLX regulates mitochondrial sodium import and calcium export. Cellular export of sodium is coupled to potassium with the sodium/potassium antiporter, linking the cellular levels of these two alkali metals together. Other cation channels that transport sodium out of lysosomes have been identified, namely, two-pore channel (TPC) and transient receptor potential mucolipin (TRPML). Potassium is one of the most abundant cations in the extracellular matrix (ECM) and regulation of metal homeostasis between the ECM and the cell is essential to maintain cellular functions.^51,52^ Potassium can be imported into cells by the sodium/potassium ATPase^53,54^ or the symporter NKCC, which imports potassium, sodium, and chloride into cells^46^ (Figure 4). Potassium export from cells on the contrary is mediated by potassium channels, including Kv1.3, Kv10.1, and Kv11.1 and calcium-activated potassium channel K.Ca3.1. Potassium transport into mitochondria is also regulated by potassium channels, including mitoKv1.3, mitoK_ATP_, and Kir, whereas the potassium/proton exchanger (KHE) mediates export of potassium from this organelle. The transmembrane protein 175 (TMEM175) has been described as being able to import and export potassium from and into lysosomes. However, it was later reported to act as a proton-activated proton channel. Thus, its contribution to lysosomal potassium homeostasis should be revisited.^55−57^ In addition, voltage-gated potassium channels, such as Kv2, mediate potassium import into the endoplasmic reticulum (ER).

### 2.2 Cellular Functions of Sodium and Potassium

The presence of the NCX antiporter at the cell membrane makes sodium a regulator of intracellular calcium levels. In addition, the sodium/proton antiporter NHE1 plays a crucial role in the regulation of cellular pH, and thus sodium can act as a regulator of intracellular pH.^58^ Importantly, proteins of the NHE family are also integral members of the mitochondrial membrane and responsible for the generation of a proton gradient, which involves pumping protons against their electrochemical gradient across the inner mitochondrial membrane. This process is essential for the production of ATP in eukaryotic cells, and thus, sodium also plays a pivotal role in energy generation via oxidative phosphorylation in the electron transport chain (ETC).^59^ High sodium levels and redistribution of sodium in mitochondria impact the ETC, which leads to expression of inflammatory genes in immune cells.^60^ Thus, sodium is a key regulator of cell metabolism. Sodium also acts as an allosteric regulator of protein function, as exemplified by the opioid receptors, which are a type of G protein-coupled receptor (GPCR), whose folding and signaling activity are influenced by sodium.^61^ Furthermore, sodium, potassium, and magnesium cations impact folding of RNAs and resulting functions.^62^ In particular, alkali metals facilitate the folding of G-quadruplex (G4) DNAs and RNAs.^63−65^ G4 are believed to be involved in a plethora of cellular functions and biological effects,^65−68^ including gene transcription and translation, DNA replication, genome instability, and telomere maintenance.^69,70^ With its unique structure, chromatin operates as a complex signaling platform.^17^ In this context, G4 and other nucleic acid structures represent cell signaling elements. Therefore, alkali metals directly impact cell signaling, affecting the stability and integrity of the nucleic acid structures. In nerve cells, sodium plays a crucial role for the transmission of action potential, where rapid sodium influx and potassium efflux generate potentials across cell membranes.^71^ Sodium influx into astrocytes is mainly regulated by glutamate transporters, whereas efflux is mediated by the sodium/potassium ATPase.^53,54^ Mitochondrial potassium channels also exhibit a cytoprotective function, although the underlying signaling pathways are not clear and require further investigation.^72^ In addition, voltage-gated potassium channels influence the capacity of the plasma membrane to regulate potassium concentrations and thus cell volume via osmotic pressure.^73^ This can affect cell cycle progression, cell proliferation, and ultimately cell death mechanisms including apoptosis.^74,75^ Furthermore, potassium has been shown to activate the mitogen-activated protein kinase kinase/extracellular-signal-regulated kinase (MEK/ERK) pathway, impacting the expression of genes implicated in cell proliferation.^76−78^ Using DNA nanodevices that can detect sodium^79^ or potassium^80^ with subcellular resolution, it was recently shown that sodium and potassium gradients exist across cell membranes. This work led to the discovery that Kv11.1 is an endosomal potassium channel.

### 2.3 Sodium and Potassium Signaling and Diseases

Alteration of sodium homeostasis has been reported in various pathophysiological contexts including cancer, ischemia-reperfusion injury, and cardiovascular diseases.^81,82^ Hyper- or hypokalemia can arise in patients where potassium homeostasis is perturbed at the cellular and/or organismal levels.^83^ Hyperkalemia can lead to thrombocytosis, hemolysis, and high white cell counts, whereas hypokalemia can cause muscle weakness and cramps and lead to arrhythmia.

#### 2.3.1 Sodium and Potassium Signaling in Cancer

Solid tumors have been shown to contain increased levels of sodium. It has been proposed that sodium ions control osmolarity in the cancer microenvironment, potentially impacting cell metabolism and immune function.^81^ Since sodium and ATP production are coupled via the mitochondrial membrane potential, these changes in sodium levels go hand in hand with metabolic alterations. This can directly impact signaling pathways that require phosphorylation events, including Kirsten rat sarcoma virus (K-ras) and mitogen-activated protein kinase (MAPK) signaling.^84^ In addition, since sodium transport is regulated by various antiporters, any changes in sodium homeostasis also impact calcium, glucose, and magnesium levels as well as pH, affecting other cellular processes.^50^ Increased levels of VGSC channels have been observed in breast cancer cells with higher metastatic potential and in breast cancer metastases.^85,86^ This suggests that increased sodium levels in cancer cells are correlated with the acquisition of the metastatic phenotype. Tetrodotoxin (Table 1) is a toxic natural product found in pufferfish. It has been shown to inhibit VGSC channels and to impact cancer cell growth.^85,87^ Ranolazine, another VGSC inhibitor, has also been shown to be effective against metastatic breast cancer.^88,89^ The VGSC inhibitor valproate has been selected for the treatment of cervical cancer.^90^ The VGSC inhibitor phenytoin^91^ inhibits breast cancer growth in preclinical models, and other inhibitors against these classes of sodium channels are under development for the treatment of cancer, including ropivacaine,^81,92,93^ carbamazepine,^87,92,93^ lamotrigine,^92,93^ fosphenytoin,^92,93^ and others (Table 1). These examples highlight that targeting voltage-gated sodium channels may be exploited for the treatment of various cancers, including metastatic disease. Additional research is required to dissect the effect of VGSC on cell signaling in metastatic disease, specifically to better characterize the role of sodium in the acquisition of distinct states of cancer cells. This may illuminate drug effects and reveal how to more effectively exploit sodium signaling in clinical settings. Another class of sodium transporters, NKCC1, has been shown to be upregulated in metastatic hepatocellular carcinoma (HCC),^94^ and the inhibitor bumetanide has been demonstrated to impact tumor growth and metastases in preclinical models of HCC.^81,94^ The sodium-dependent glucose transporters SGLT enable the cellular uptake of glucose, representing an alternative mechanism to the canonical pathway implicating glucose transporters (GLUTs). SGLT transporters are highly expressed in pancreatic and prostate adenocarcinomas, and SGLT2 inhibitors such as canagliflozin (Table 1), which is clinically approved for the treatment of diabetes, showed reduction of pancreatic cancer in xenograft models.^95^ Amiloride is a blocker of ENaC channels and has been shown to inhibit growth of solid tumors.^81,96−98^ Additionally, NMDAR blockers including memantine and MK-801 have been shown to reduce growth of breast cancer in human xenografts.^81,99^ Together, these studies on sodium receptors illustrate how cellular homeostasis of alkali metals is dysregulated in cancer and how targeting specific channels or transporters can be exploited for therapeutic benefits. Small molecules provide the means to dissect metal ion signaling in great detail. Understanding how sodium impacts cancer cell fate and contributes to metastasis and drug-tolerance requires further investigation.

**Table 1:**
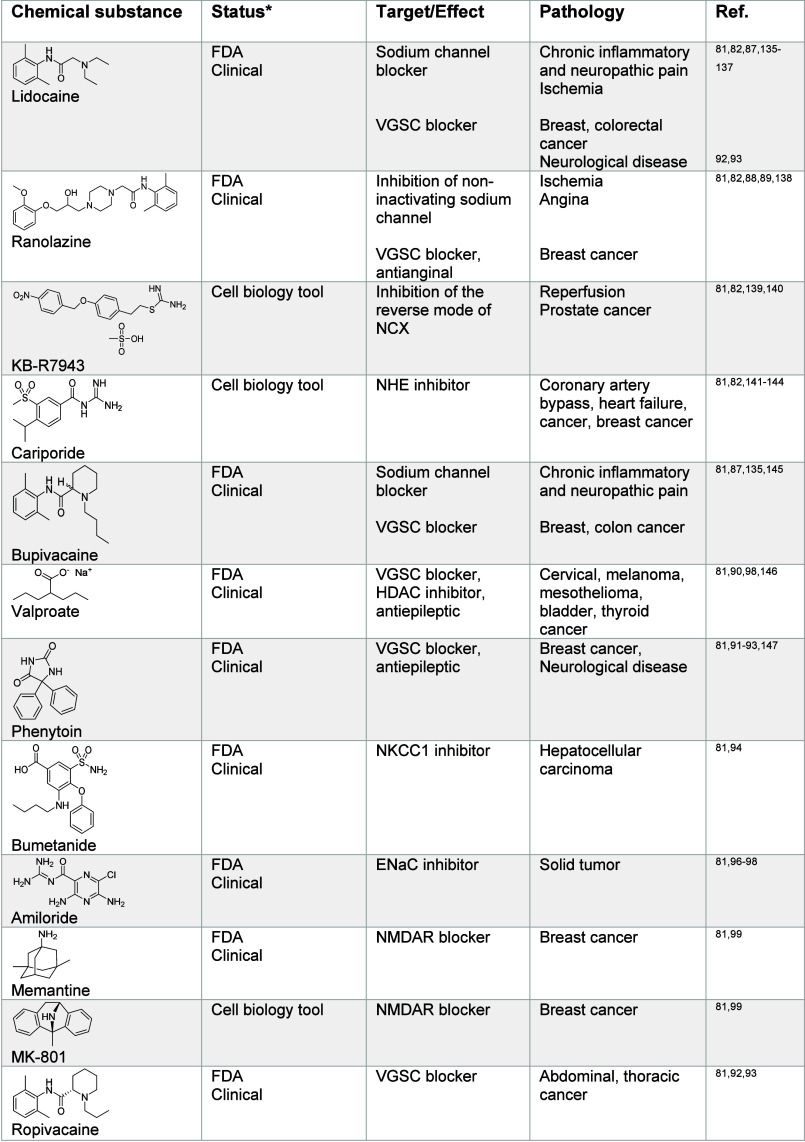

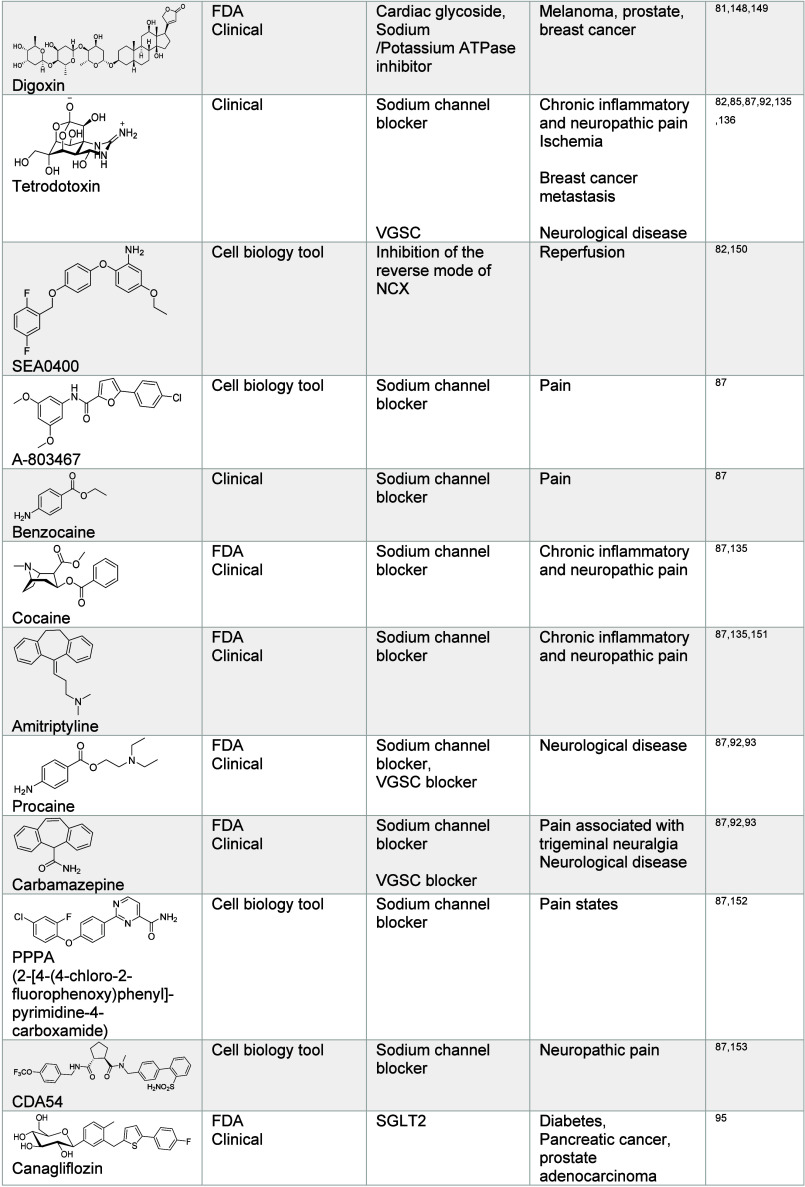

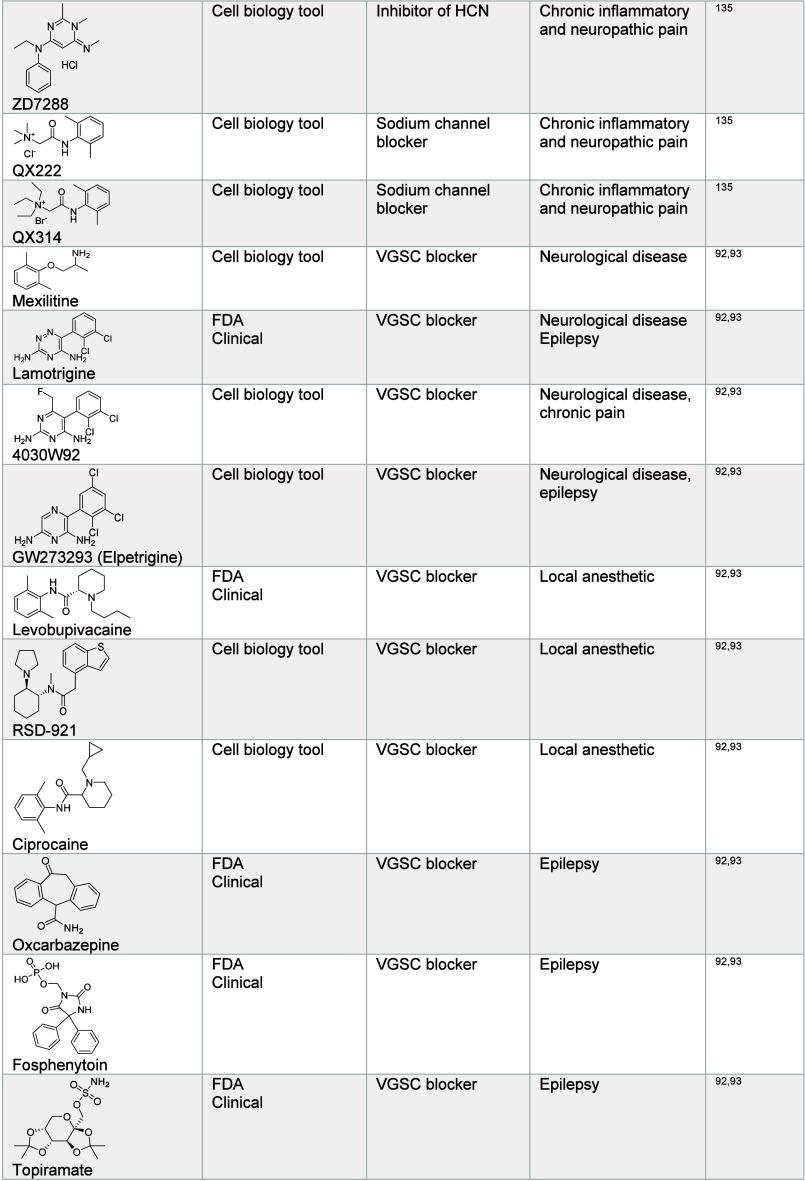

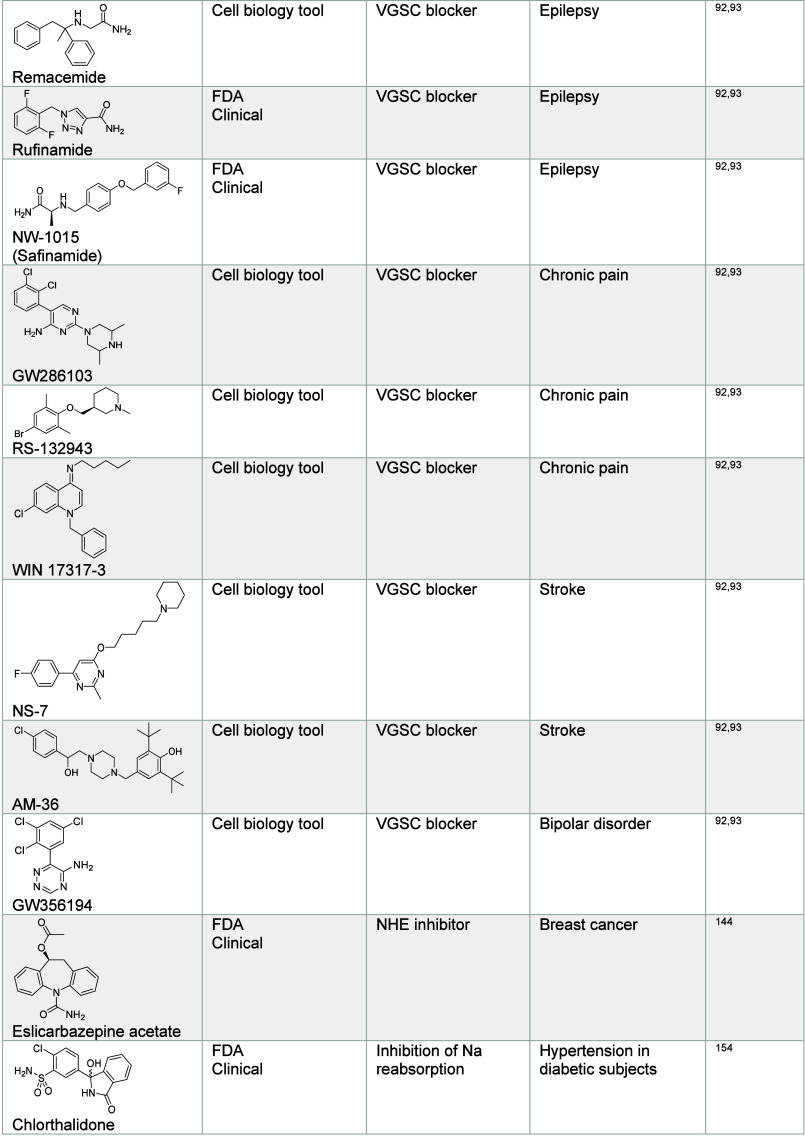
Regulators of Sodium Signaling^81,82,85,87−89,91−99,135−154^

* https://www.fda.gov, https://clinicaltrials.gov

Potassium has been reported to be overabundant in the tumor microenvironment.^100^ It was argued that elevated potassium concentrations lead to perturbations in the electrochemical gradient required for the uptake of nutrients in T cells. This can lead to a reduction in histone acetylation at genes required for T cell effector function. This, in turn, was found to improve T cell multipotency and tumor clearance capacity. In another study, it was shown that distinct cancer types exhibit a reduced expression of the potassium channel Kv1.5 at the cell membrane.^101^ These cells could be sensitized to apoptosis by dichloroacetate, which causes an increased level of expression of Kv1.5. This promoted hyperpolarization of the cell and an inhibition of voltage-dependent entry of calcium, causing decreased glycolysis and increased mitochondrial respiration. Potassium channels have garnered a great deal of interest in cancer research because a dysregulation of these channels has been observed in many cancers, supporting a causal role of potassium in cancer progression, suggesting that targeting potassium signaling, or more broadly cellular potassium homeostasis, can be exploited for therapeutic intervention. For instance, small molecules such as quinidine and quinine (Table 2), block potassium channels with pro-apoptotic properties in glioma cells.^75,102−106^ TRAM-34 targets calcium-activated potassium channels and is under development for the treatment of glioblastoma.^107−109^ Dequalinium and amiodarone target potassium channels in breast cancer cells^105,110−112^ and mitochondrial Kv1.3 channel inhibitors like clofazimine or margatoxin represent promising therapeutic candidates for the treatment of lymphoma and lung adenocarcinoma.^110,113,114^ In addition, the Kv and Kir family blocker cisapride is in development for the treatment of gastric cancer.^110,115^ The antibiotic erythromycin also inhibits these channels^110,116,117^ and is under investigation for the treatment of cancer, autoimmune, and neurodegenerative disorders (for a list of sodium and potassium channel inhibitors, see Table 2). These examples highlight how targeting potassium channels and related signaling networks could provide powerful therapeutic opportunities in cancer. Furthermore, G4 structures, stabilized by potassium in the inner cavity composed of guanine residues are atypical chromatin targets that may provide the basis for drug design.^118,119^ For instance, the small molecule pyridostatin (Table 2) has been shown to target G4 in gene bodies and at telomeres, leading to transcription and replication-dependent DNA damage, activating a DNA damage signaling response and apoptosis in cancer cells.^69,120,121^ Pyridostatin was used in many laboratory settings^122−124^ to challenge the existence and dissect the biology of G4 nucleic acids in the cell, lending strong support to the idea that such structures, whose dynamic folding relies on alkali metals, can be exploited beyond academic research.

**Table 2:**
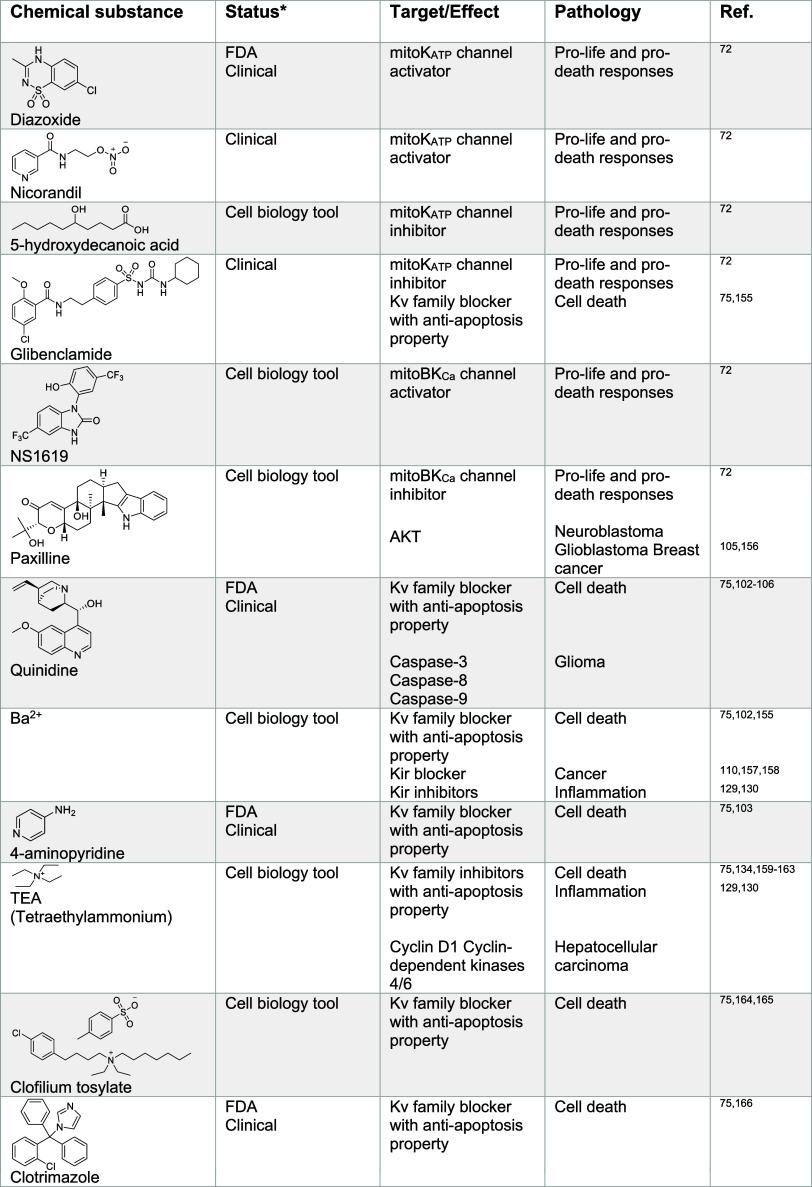

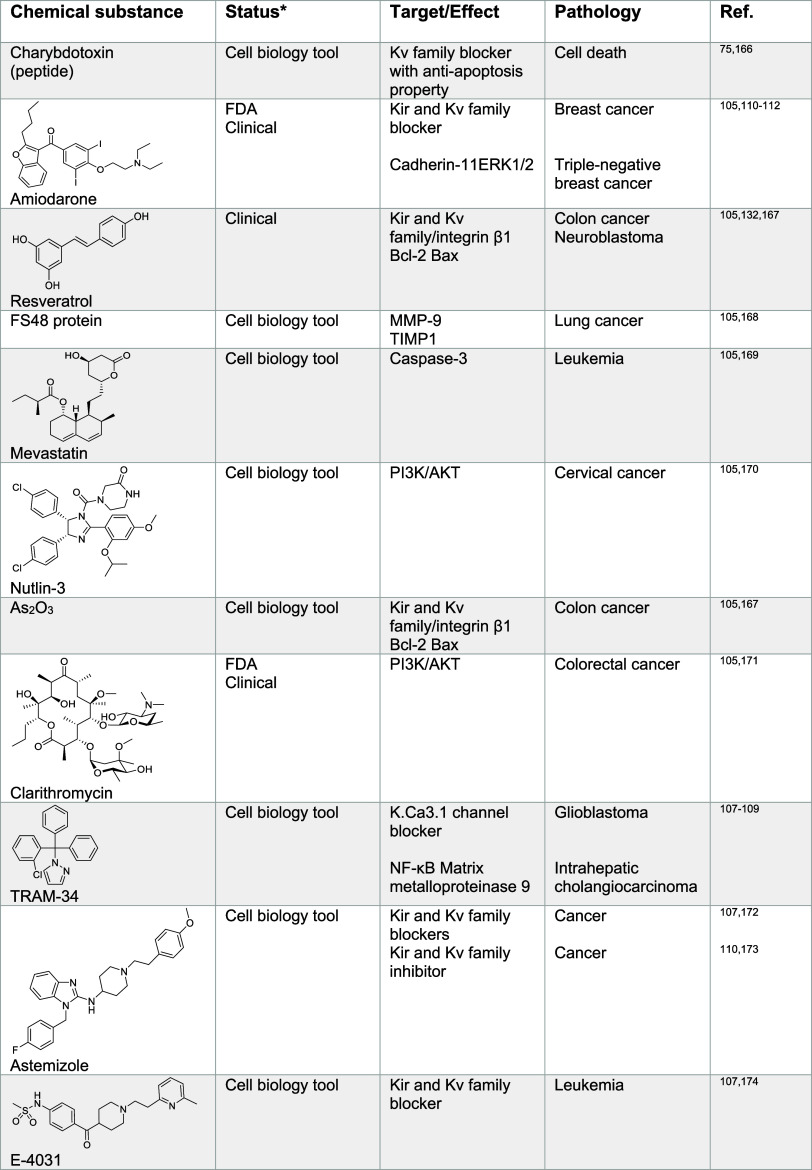

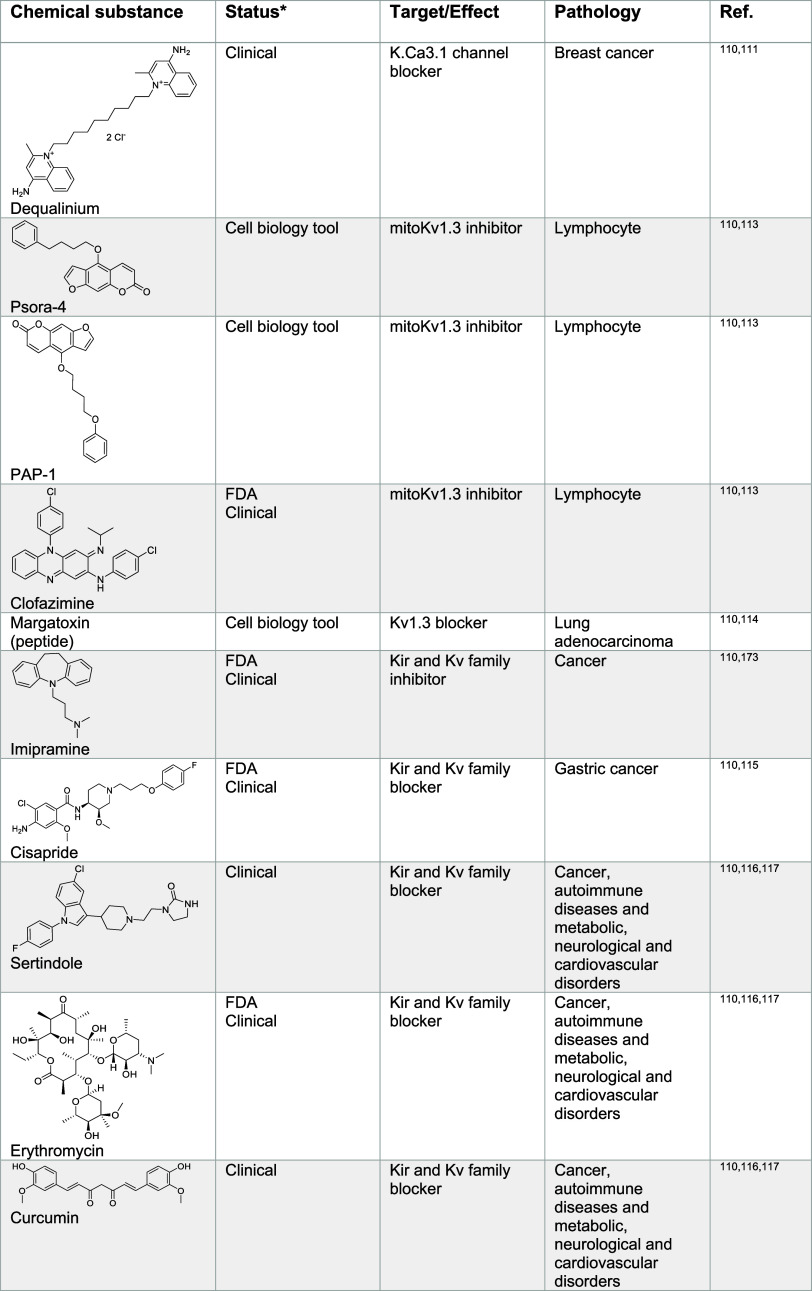

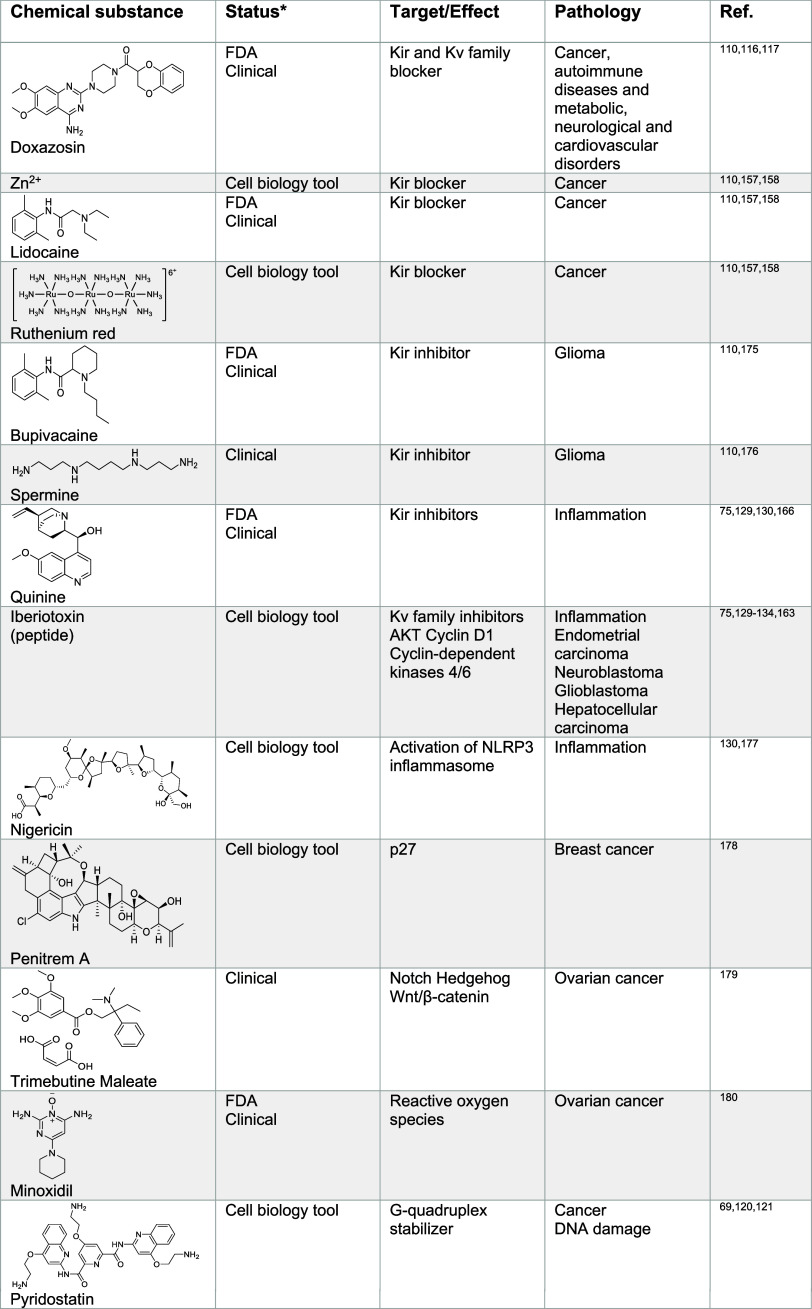
Regulators of Potassium Signaling^72,75,102−117,120,121,129−134,155−180^

* https://www.fda.gov, https://clinicaltrials.gov

#### 2.3.2 Sodium and Potassium Signaling in Immunity and Inflammation

The role of sodium in immune cell activation is complex. Concentrations of sodium chloride were found to be elevated in human and mouse skin infections. In a model of bacterial skin infection by *Leishmania major*, high levels of sodium chloride were found to lead to (p38/MAPK)-dependent nuclear factor of activated T cells 5 (NFAT5) signaling activation and epigenetic alterations in inflammatory macrophages.^125^ Explicit roles of sodium in this context remain incompletely understood and require further efforts. ERK1 and ERK2 activation was also observed in inflammatory macrophages under high sodium level conditions.^126^ In contrast, acquisition of the anti-inflammatory state of macrophages was inhibited under high sodium concentrations, reducing serine/threonine protein kinase (AKT) and mammalian target of rapamycin (mTOR) signaling.^127^ Given the complexity of mechanisms underlying activation of macrophages in vivo^128^ and that macrophage populations can shift between states, the contributions of sodium on macrophage plasticity in vivo requires additional investigation. Potassium has also been documented to play a role in inflammation since blocking potassium channels has been shown to attenuate inflammation.^129−134^ The role of potassium channels in inflammatory settings suggests that targeting key channels with barium ions, tetraethylammonium, iberiotoxin, as well as the FDA-approved drug quinine^129−134^ (Table 2) may be exploited in clinical settings to control immune responses and reduce inflammation.

## 3 Alkaline Earth Metal Ion Signaling

### 3.1 Magnesium Signaling

#### 3.1.1 Regulation of Magnesium Homeostasis

**Figure 5:**
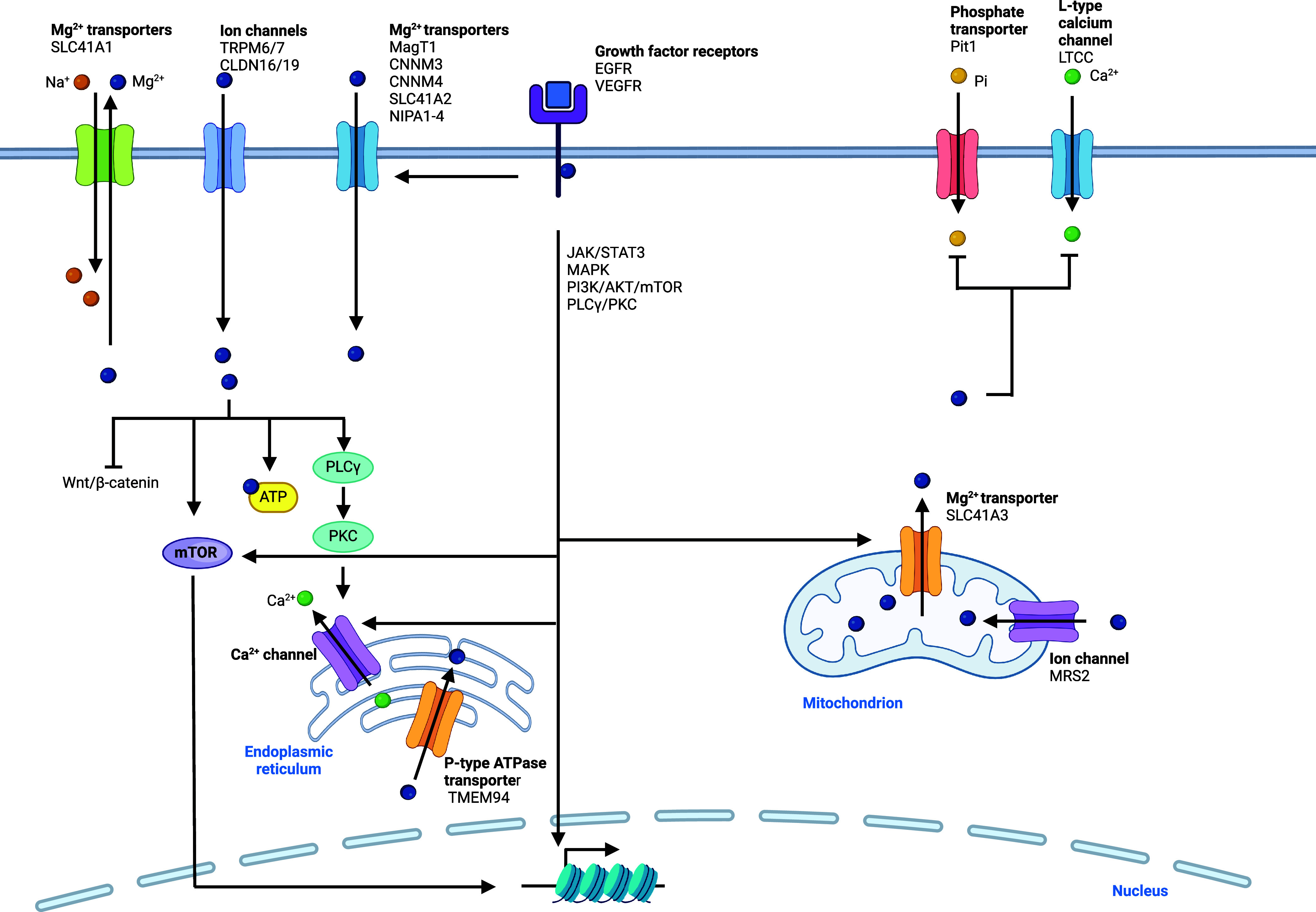
Magnesium signaling. Magnesium is taken up into the cell by ion channels and transporters. Activation of growth factor receptors, such as EGFR, increases magnesium uptake. Magnesium is trafficking to mitochondria via SLC41A3 and MRS2. In the cytosol, magnesium activates mTOR, allows calcium release from the ER, regulates ATP binding, and inactivates Wnt/β-catenin signaling. Magnesium inhibits the phosphate transporter and L-type calcium channel. Magnesium is exported outside of the cell by a magnesium transporter. See Abbreviations. Figure generated with BioRender.com.

In the cell, magnesium is found as a +2 oxidation state, as both a free and bound metal ion, and it is the second most abundant intracellular metal after potassium. Magnesium is imported into the cell through ion channels and transporters. Notably, magnesium channels and transporters include transient receptor potential melastatin 6/7 (TRPM6/7)^181,182^ and the magnesium transporter MagT1^183^ (Figure 5). Since TRPM7 also enables transport of zinc and calcium, strategies designed to target these receptors potentially affect intracellular levels of other metals.^184^ SLC41A2 is also a cell membrane specific magnesium transporter,^185^ and a proton/magnesium exchanger was found to play a major role in cellular magnesium import.^186^ In renal epithelial cells, claudin-16 (CLDN16) and CLDN19 are involved in divalent metal import, including magnesium.^187^ Magnesium transporters responsible for transport into and out of cell organelles have been described. These include mitochondrial magnesium channel MRS2^188,189^ and mitochondrial magnesium export protein SLC41A3.^190^ Magnesium is also found abundantly in the ER, and TMEM94 has been described as an ER-specific magnesium transporter.^191^

#### 3.1.2 Cellular Functions of Magnesium

Magnesium plays key roles in the cell as a regulator of metabolic processes. It affects the activity of a large number of proteins, exerting an activity by directly binding to substrates, such as ATP,^192^ binding to the active sites of enzymes, or acting by means of metalloallostery, promoting enzyme complex formation. By doing so, magnesium controls the function of various enzymes and ion channels. It can also promote the folding of nucleic acids, impacting on functions. It plays a role in many fundamental biological processes including DNA replication, transcription and RNA translation.^193^ Indeed, magnesium stabilizes DNA and RNA structures and many enzymes involving nucleic acid processing require magnesium as a cofactor.^194^ Magnesium can act as a potent antagonist of calcium channels, such as L-type calcium channel (LTCC), and thus, magnesium levels can impact smooth muscle function, where fast calcium release and subsequent calcium binding to calmodulin regulates smooth muscle contraction.^195,196^ This exemplifies how a metal can influence the biological effect of another in this setting by directly controlling the influx of other metals via specific cellular transporters. In muscle cells, magnesium supplementation has been shown to activate mTOR signaling.^197^ It was argued that excess magnesium could promote the formation of magnesium–ATP complexes, thereby promoting phosphorylation cascades and stimulating mTOR signaling. Since other signaling pathways require phosphorylation and magnesium is essential for the activity of kinases, magnesium can stimulate other signaling cascades, including Janus kinase/signal transducer and activator of transcription (JAK/STAT) and MAPK.^198,199^ This might also explain how magnesium can promote osteogenic differentiation of mesenchymal stem cells.^198^

#### 3.1.3 Magnesium Signaling and Diseases

Magnesium levels are altered in several human disease settings, including cancer, cardiovascular diseases, neurological disorders, renal disorders, and diabetes.^200,201^ In particular, many cardiovascular defects are associated with reduced cellular magnesium influx and increased intracellular calcium levels. Thus, magnesium supplementation can actually alleviate some of the associated symptoms.^202,203^ Small molecules that chelate magnesium have been developed as laboratory tools to study cardiovascular, neurodegenerative, and renal diseases. These include ethylenediaminetetraacetic acid (EDTA), aminophenol triacetic acid (APTRA)^202^ and aminophenol-*N*,*N*-diacetate-*O*-methylene-methylphosphinate (APDAP)^203^ (Table 3), although some of these compounds can adversely alter metal homeostasis more broadly, lacking specificity for magnesium binding.

##### 3.1.3.1 Magnesium Signaling in Cancer

In cancer, the contribution and subsequent effects of magnesium are also complex. Although an increase in magnesium levels has been reported in neoplastic cells, it remains unclear whether this effect is due to alterations of calcium levels and signaling or even that of other metals. It has been suggested that increased magnesium concentrations provide cancer cells with a metabolic advantage, in particular as it is needed for ATP production.^206^ Various studies have illustrated the value of targeting magnesium import into cells, for instance by using blockers of TRPM7, such as waixenicin A^207^ (Table 3). This strategy holds great promise for the clinical management of cancer.^208,209^ In addition, since magnesium is crucial for kinase function, signaling pathways like MAPK, JAK/STAT, and others can be impacted by changes in magnesium homeostasis.^184^ Indeed, several growth factors such as epithelial growth factor receptor (EGFR) and vascular endothelial growth factor receptor (VEGFR) can induce signaling pathways involving effector kinases downstream in the signaling cascade, including JAK/STAT3, MAPK, phosphatidylinositol-3 kinase (PI3K)/AKT/mTOR, and phospholipase C γ/protein kinase C (PLCγ/PKC).^184^ Thus, it will be important to better characterize the effect of small-molecule modulators of magnesium homeostasis in cancer.

**Table 3:**
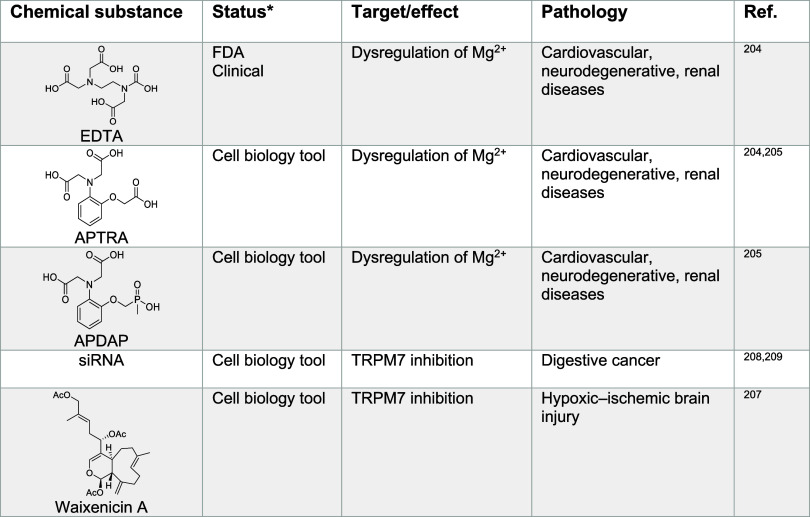
Regulators of Magnesium Signaling^204,205,207−209^

* https://www.fda.gov, https://clinicaltrials.gov

##### 3.1.3.2 Magnesium Signaling in Immunity and Inflammation

Functional magnesium signaling has been documented in immune cells.^210,211^ For instance, mutations in MagT1 have been reported in several diseases, including an X chromosome-linked immunodeficiency characterized by CD4+ lymphopenia and in defective T cell activation. In particular, the latter setting hinted toward a direct effect of magnesium impacting cell states. In this case, T cell receptor signaling has been shown to be impaired upon changes in the intracellular magnesium levels. This leads to alteration of PLCγ1 function and reduction of inositol triphosphate (IP_3_) synthesis, impacting calcium signaling. Another report documented that lymphocyte function-associated antigen 1 (LFA-1) requires magnesium to adopt its active conformation to mediate signaling in T cells, which in turn increases calcium flux, leading to perturbation of cell signaling downstream.^212^ In neurons, γ-aminobutyric acid type A (GABA_A_) receptor signaling triggers release of magnesium from mitochondria, which impacts signaling pathways downstream, involving mainly calcium-dependent signaling, including ERK, cyclic-AMP responsive element-binding protein (CREB), and mTOR.^213^ Patients with non alcoholic steatohepatitis (NASH) are characterized by dysregulated magnesium levels in liver cells due to increased expression of the magnesium transporter CNNM4.^214^ This opens the possibility of targeting this transporter to potentially control this inflammatory disease. Interestingly, acetaminophen overdose causes liver inflammation, magnesium dysregulation, and CNNM4 overexpression.^215^ Taken together, targeting CNNM4 and magnesium balance in the liver might constitute a therapeutic route to treat an array of inflammatory liver diseases and disorders.

### 3.2 Calcium Signaling

#### 3.2.1 Regulation of Calcium Homeostasis

**Figure 6:**
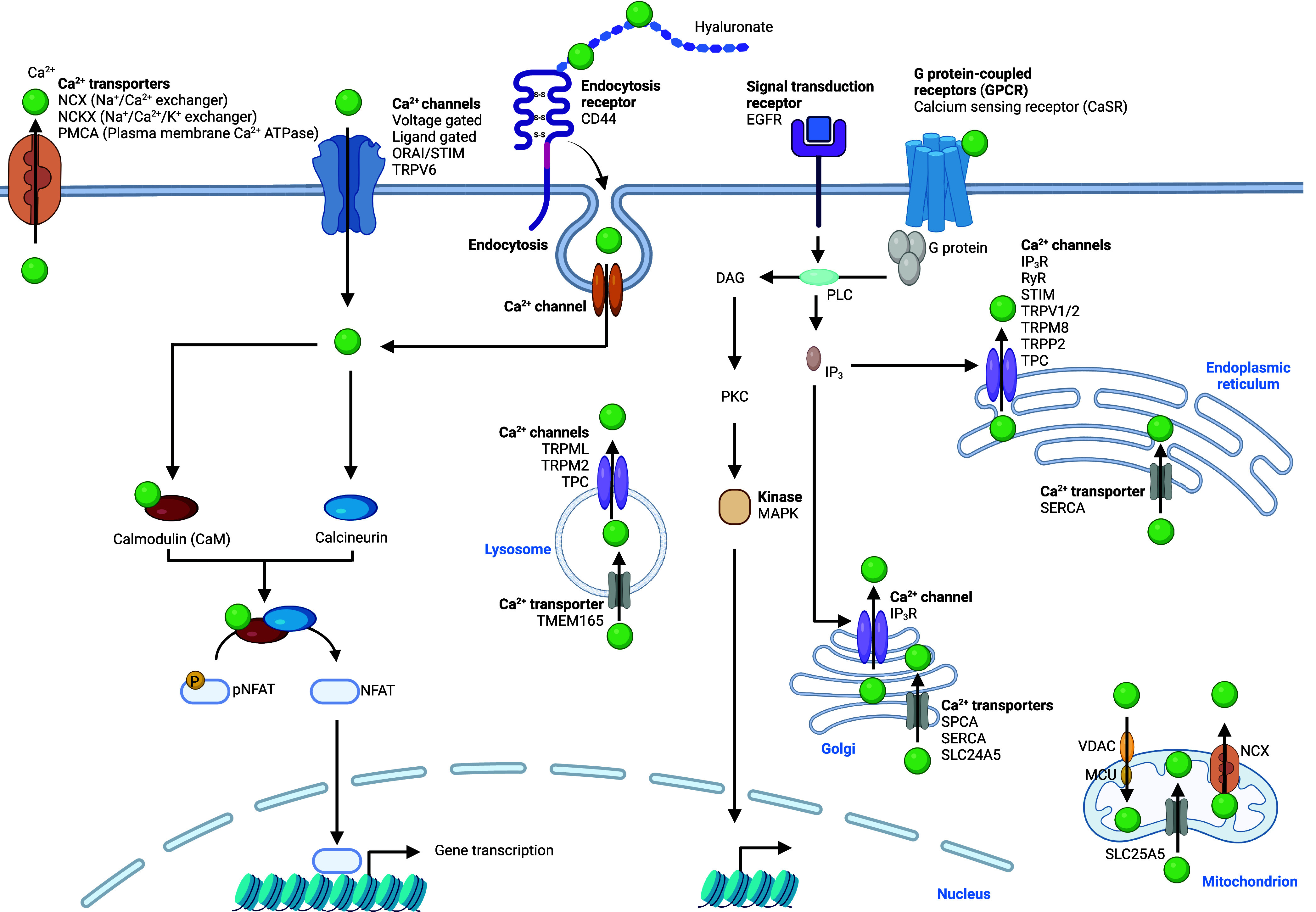
Calcium signaling. Calcium is taken up into the cell via ion channels or by means of CD44/hyaluronan-mediated endocytosis. Intracellular calcium is trafficking to the ER, lysosomes, the Golgi apparatus, and mitochondria. The activity of several proteins is modulated by calcium including the phosphatase calcineurin, the ubiquitous calcium-binding protein CaM and the TF NFAT. Binding of hormones to GPCR triggers IP_3_ production, which releases calcium from the ER and the Golgi apparatus. Calcium is exported outside the cell by calcium transporters. See Abbreviations. Figure generated with BioRender.com.

Calcium ions are found in a +2 oxidation state. Calcium is abundant in the cell, and cytosolic concentrations as a free ion are low, being mostly bound to biomolecules. Calcium levels within organelles are tightly controlled.^32,216−218^ Several mechanisms regulating cellular calcium levels have been identified, involving cellular import and export proteins.^219−222^ Mechanisms of calcium uptake are diverse, the most thoroughly characterized are ion channels,^223^ including voltage-gated and ligand-gated channels as well as calcium release-activated calcium modulator/stromal interaction molecule ORAI/STIM (Figure 6). ORAI/STIM consists of the pore-forming proteins ORAI 1 to 3 and STIM 1 and 2.^224^ Calcium can bind to and activate the family of calcium-sensing receptors (CaSR),^225^ which are GPCR that regulate calcium levels in the blood. Calcium uptake has been shown to be sensitive to poisoning and inactivation by other metals,^226^ including magnesium, cobalt, nickel, cadmium, and manganese ions. These observations support the notion that levels of distinct metals can impact calcium signaling.^227^ Besides lipid membrane channels that can transport calcium, other studies have shown that calcium can also be taken up by endocytosis,^228^ and calcium release from late endosomes and lysosomes is pH-dependent. For example, it has been shown that calcium can be taken up via the plasma membrane glycoprotein cluster of differentiation 44 (CD44)/hyaluronan metal endocytosis pathway in cells undergoing cell state transitions, specifically in macrophages acquiring a pro-inflammatory cell state^13^ and, indeed, calcium signaling is crucial for macrophage activation and function.^229^

Complex machineries have been documented to be responsible for the release of calcium from cell organelles. Calcium is apparently not translocated from the endolysosomal compartment via divalent metal transporter 1 (DMT1).^230^ Other ion channels purportedly play that role. Using a calcium-dependent fluorescent pH-dependent reporter molecule (CalipHluor), ATPase cation transporting 13A2 (ATP13A2) has been shown to affect intracellular calcium levels^231^ and to facilitate lysosomal calcium import.^218^ Given its role in polyamine transport, it remains to be elucidated how this calcium transport is facilitated.^232^ The calcium/proton exchanger CAX was identified and suggested to be implicated in translocation of calcium into lysosomes.^233^ Recently, transmembrane protein 165 (TMEM165) has been identified as an importer of calcium into lysosomes.^234^ In addition to these importers, calcium has been shown to be exported from this compartment by the calcium channels TRPML, TRPM2, and TPC.^219−222^ This network of proteins highlights the importance of lysosomal calcium homeostasis for cells and provides the means to manipulate lysosomal calcium pools for cellular functions. In the cell, IP_3_ is a key signaling molecule that regulates calcium homeostasis (Figure 6). IP_3_ can bind to the IP3 receptor (IP_3_R), which is mainly situated at the membrane of the ER and the Golgi apparatus, where it causes the release of calcium ions into the cytosol. Ryanodine receptor (RyR) represents another class of receptors mostly located at the membrane of the ER or the sarcoplasmic reticulum (SR) in cardiomyocytes. These receptors can also release calcium into the cytosol upon stimuli, defining a positive feedback mechanism that allows rapid calcium build-up for muscle contraction. Other calcium channels that export this metal ion from the ER include STIM, transient receptor potential vanilloid type 1 (TRPV1), TRPM8, transient receptor potential polycystin-2 (TRPP2), and TPC (Figure 6).^219−222^ The calcium transporter sarcoplasmic/endoplasmic reticulum calcium ATPase (SERCA) has been reported to translocate calcium into the ER and the SR in muscle cells.^235^ SERCA as well as secretory pathway calcium ATPase (SPCA) are transporters that import calcium into the Golgi apparatus.^236^ SLC25A5 can also transport calcium into mitochondria and SLC24A5 to the Golgi apparatus.^237^ It has been shown that mitochondrial calcium uniporter (MCU) interacts with voltage-dependent anion channel (VDAC) to mediate calcium transport to mitochondria,^238^ and the calcium transporter NCX can export this metal from mitochondria. Calcium export from cells is mediated by different transporters, including the antiporters NCX, NCKX, and the calcium ATPase PMCA.^223^ Taken together, this complex network of proteins advocates for a prevalent role of calcium signaling, where levels in the cytosol and in different organelles are tightly controlled in a cell type-specific manner.

#### 3.2.2 Cellular Functions of Calcium

Calcium plays a key role in smooth muscle contraction. It binds to calmodulin in the cytosol, which in turn activates myosin light chain kinases.^195,196^ Calcium is generally released quickly via RyR and IP_3_R from the ER and the SR in specialized cells.^239,240^ Calmodulin is a key calcium-dependent signaling protein whose activation occurs upon binding to calcium. Activated calmodulin can then form complexes with various proteins to relay signals, including to TFs,^241^ such as CREB.^242^ The regulation of this TF takes place via calcium/calmodulin-dependent protein kinases.^243^ Importantly, the ER and the SR store intracellular calcium, whose dysregulated homeostasis has been reported in various diseases, including diabetes, cardiovascular diseases, and cancers.^244^ Given that changes in cell states can rely on alterations of calcium signaling, it is conceivable that controlling calcium levels can provide control over the acquisition of a distinct cell identity. Pioneering work illuminated calcineurin and NFAT^22^ signaling, a pathway that connects the cell membrane to the nucleus, which is activated by calcium and plays a central role in development and T cell activation. Calcium regulates the function of calcineurin,^245^ a phosphatase that promotes translocation of NFAT to the nucleus, thereby activating specific transcriptional programs.^25^ Calcium plays a pivotal role during the development and maturation of neurons and ultimately the formation of neural networks. The underlying gene expression programs of these cells are controlled, at least partly, by calcium via activation of specific signaling pathways.^246,247^ MAPK signaling involves ERK, and interactions between ERK and other proteins are regulated by calcium.^248^ Another study demonstrated that calcium concentrations changed during the early steps of embryonic stem-cell-derived neural precursor development. These changes were linked to a functional calcium response network and alterations in RyR receptor expression during different stages of development.^249^ Interestingly, oscillations of calcium spikes were observed during neuronal development.^250^

#### 3.2.3 Calcium Signaling and Diseases

##### 3.2.3.1 Calcium Signaling in Cancer

Specific GPCR are located in the cell membranes of particular cell types and can sense extracellular calcium levels. These GPCR are CaSR that are essentially found in neuronal cells. These receptors mediate important cellular signaling functions,^251^ relaying signals by means of induced conformational changes upon calcium binding in the extracellular matrix. For instance, it has been proposed that neural cell plasticity is strongly controlled by extracellular calcium signaling rather than intracellular calcium levels.^252,253^ CaSR dysregulations have been associated with cardiovascular diseases and cancer.^254^ In colorectal cancer, CaSR have been associated with antitumorigenic properties. In this context, receptor agonists represent interesting candidates for the treatment of colorectal cancer.^255^ Indeed, elevated intake of dietary calcium has been associated with reduced risk of colon cancer.^256^ In general, calcium levels and calcium signaling are altered in cancer cells.^257^ Signaling via these CaSR can determine cell fate, which makes them attractive potential therapeutic targets. These receptors are not solely activated by calcium, but also by molecules such as polyamines like spermine and the aminoglycoside antibiotic neomycin,^258−260^ providing a starting point for the development of new anticancer strategies. Given the effect of spermine on calcium and potassium channels (Tables 2 and 4), this opens the question of the specificity of this molecule and potentially others. Targeting calcium signaling in cancer, in particular specific calcium channels, has gained momentum with the development of several promising small molecules with therapeutic potential^261^ (Table 4). For instance, 4-chloro-*m*-cresol, suramin, and ryanodine were shown to activate RyR,^262−265^ whereas dantrolene and ruthenium red can inhibit these channels.^265,266^ IP_3_R activators, such as adenophostin A can promote calcium release from the ER.^267^ Conversely, IP_3_R inhibitors such as xestospongin B^268,269^ or heparin^270^ can block calcium translocation into the cytosol. Interestingly, xestospongin B has been shown to trigger apoptosis in neuroblastoma cells in vitro.^268^ Apoptosis has been linked to alterations of calcium homeostasis,^271^ potentially involving the B-cell lymphoma 2 (Bcl-2) protein regulating calcium fluxes^272^ and inducing ER stress. Dysregulation of ER calcium levels have been reported in cancer cells where calcium might be involved in malignant transformations, making small molecules modulators of calcium channels activity valuable cell biology tools and potential therapeutics in oncology.^273^ Other small molecule activators and inhibitors of calcium channels have been identified, including compounds targeting ORAI/STIM,^274−280^ voltage gated channels^281−288^ and others (Table 4). Notable ORAI/STIM inhibitors include *N*-methylnitrendipine (MRS-1844) and *N*-propargylnitrendipine (MRS-1845) have proven to be effective against lung cancer cells and leukemia.^276,277,289^ 1-(5-Chloronaphthalen-1-yl)sulfonyl-1,4-diazepane (ML-9) has reached clinical development for the treatment of prostate cancer.^275,276,290−292^ The Cav3.1 to Cav3.3 inhibitor amlodipine is being developed for the treatment of epidermoid carcinoma.^287^ Interestingly, inhibitors against lysosomal calcium channels are also being developed, including the FDA-approved antifungal drug clotrimazole.^293^ Although imidazole-containing antifungal agents such as clotrimazole and econazole inhibit ergosterol biosynthesis in fungi leading to cell wall damage in these organisms, they can readily coordinate transition metals such as calcium.^294^ More research is required to study the mechanism of action (MoA) of ion channel inhibition by these compounds. TRPM8 inhibitors, including cannabigerol^295,296^ and M8-B^296,297^ are being studied for the treatment of lymphoma, lung cancer, breast cancer, and prostate cancer. In addition, the TRPV1 inhibitor capsaicin^298−302^ is in developement for the treatment of renal carcinoma, and the TRPV2 inhibitor probenecid^303^ has shown promising clinical effects against glioblastoma and bladder cancer. The synthetic peptides and TRPV6 inhibitors soricidin and SOR-C13^304^ have been shown to exhibit promising results in vitro for the potential treatments of ovarian, prostate, and brain cancers. Finally, the small molecule thapsigargin^305^ and the artificial peptide G202^306^ have been reported to inhibit SERCA for the treatment against several cancers, including prostate, colon, breast, and cervical cancers. Given the complexity of calcium transport and the plethora of calcium channels present in cells, with differential levels of expression in different tissues, targeting calcium transport selectively in tumors is a challenging endeavor and should be exploited taking into account tissue-specific features.^307^ The role of inflammation in tumorigenesis^308^ and the fact that calcium signaling plays a role in the control of cell plasticity in immunity and cancer raises the prospect of targeting calcium homeostasis for the clinical management of cancer.

**Table 4:**
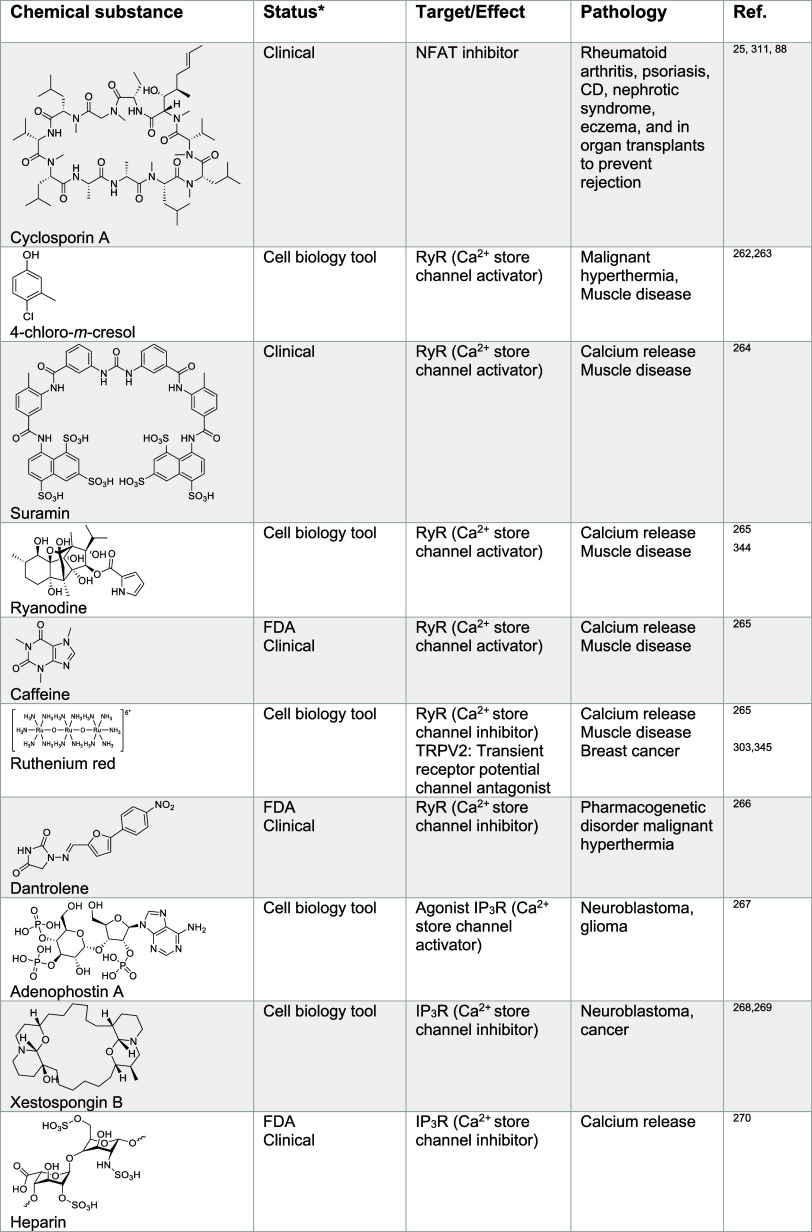

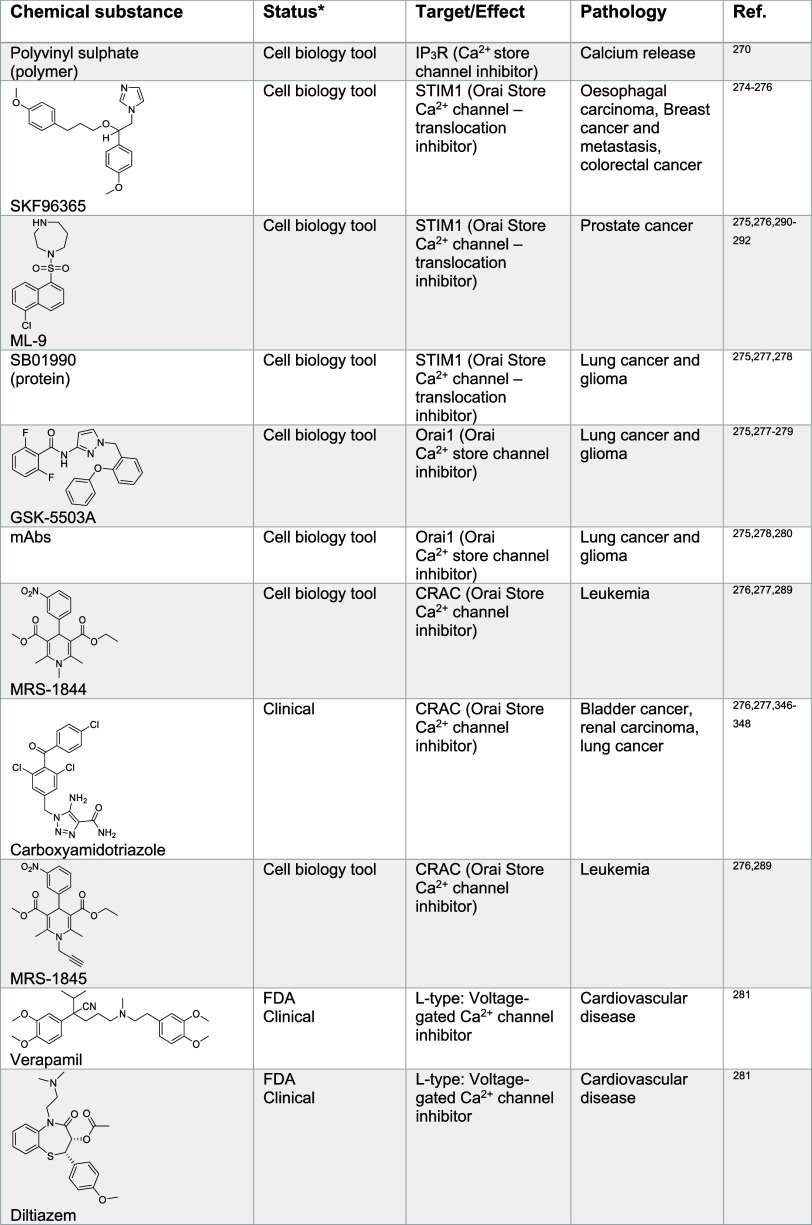

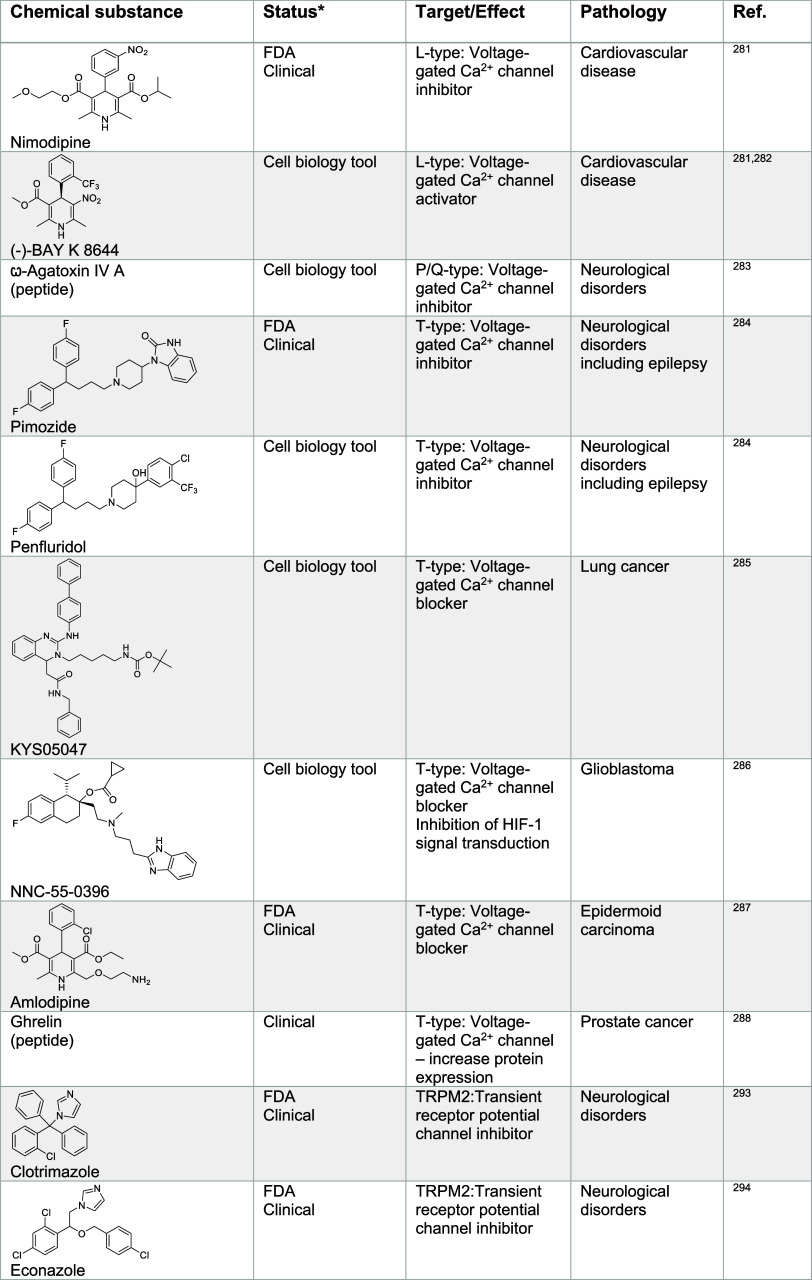

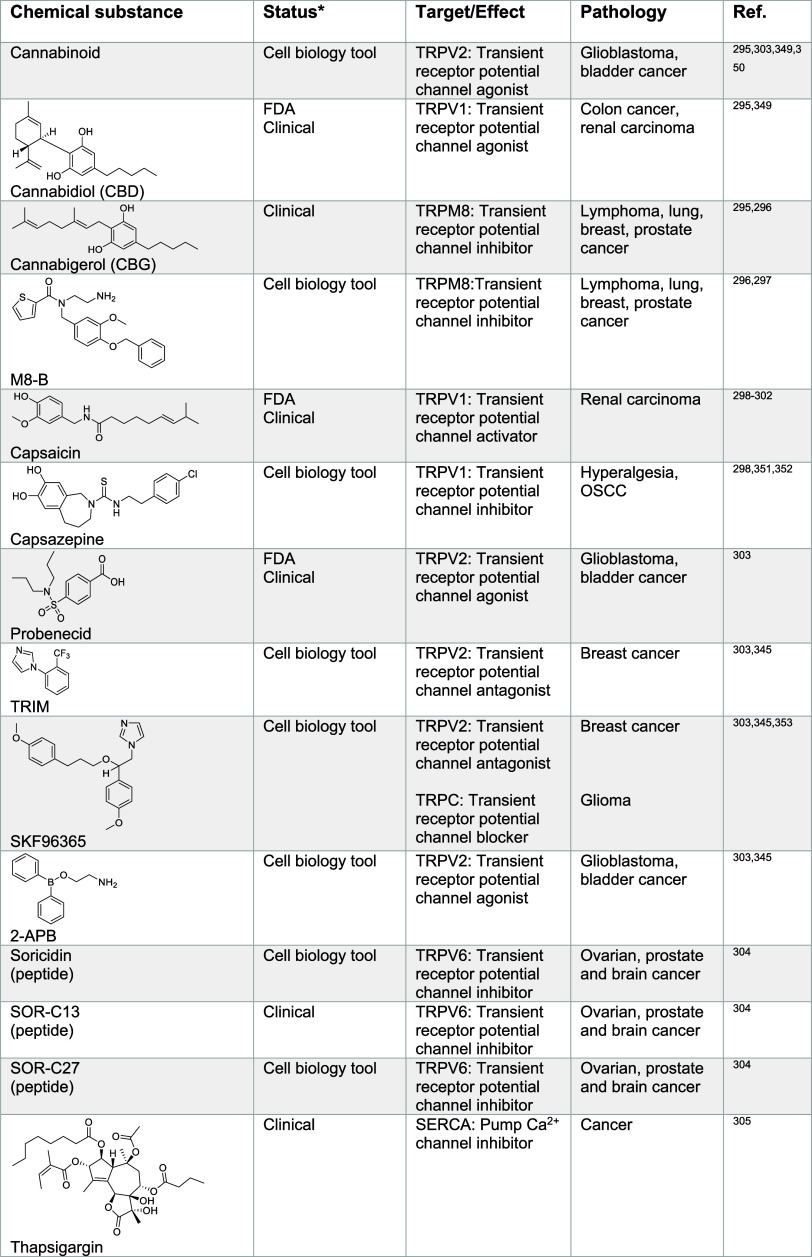

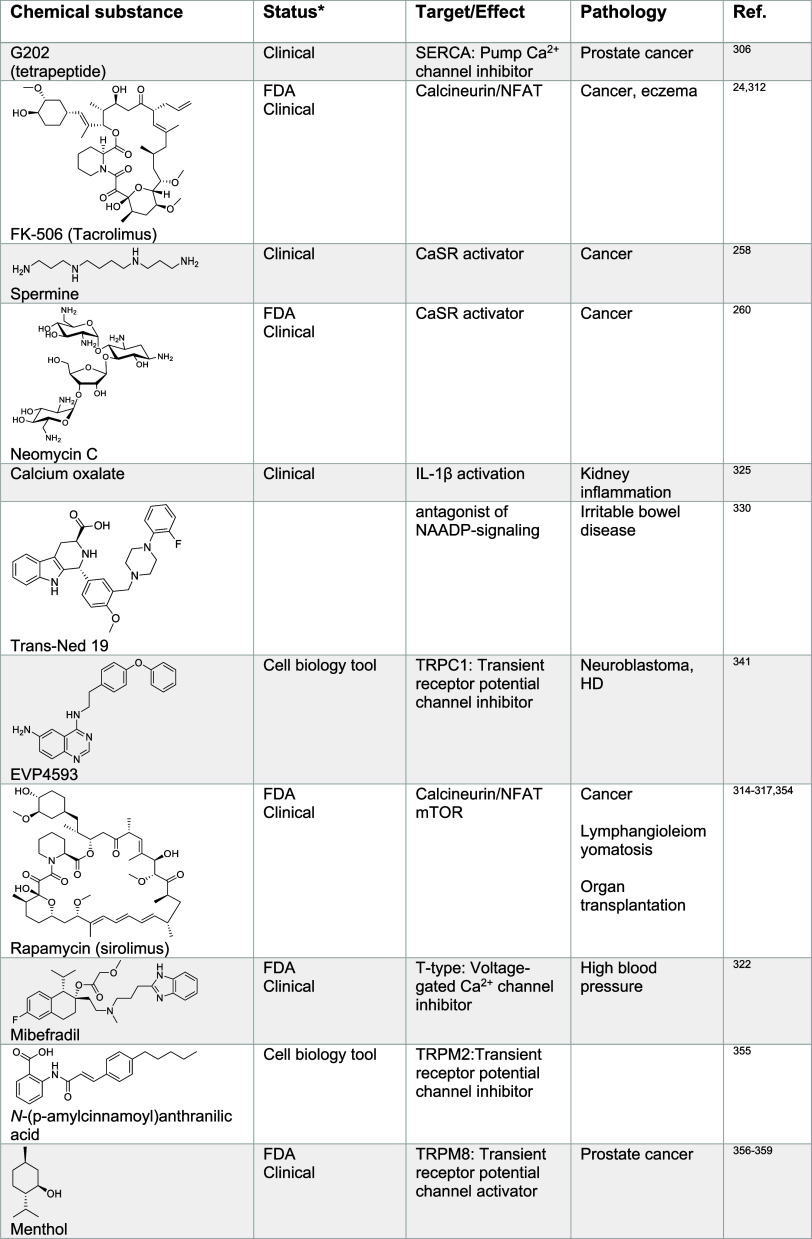

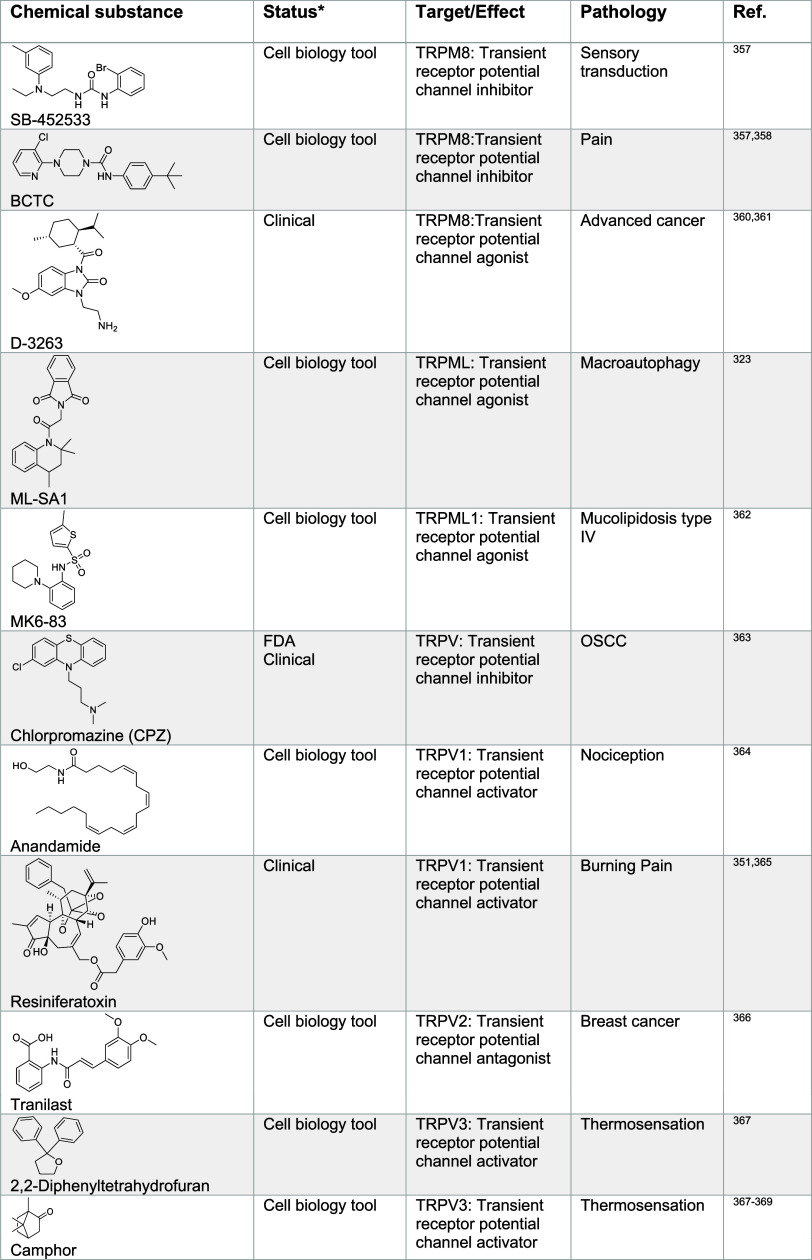

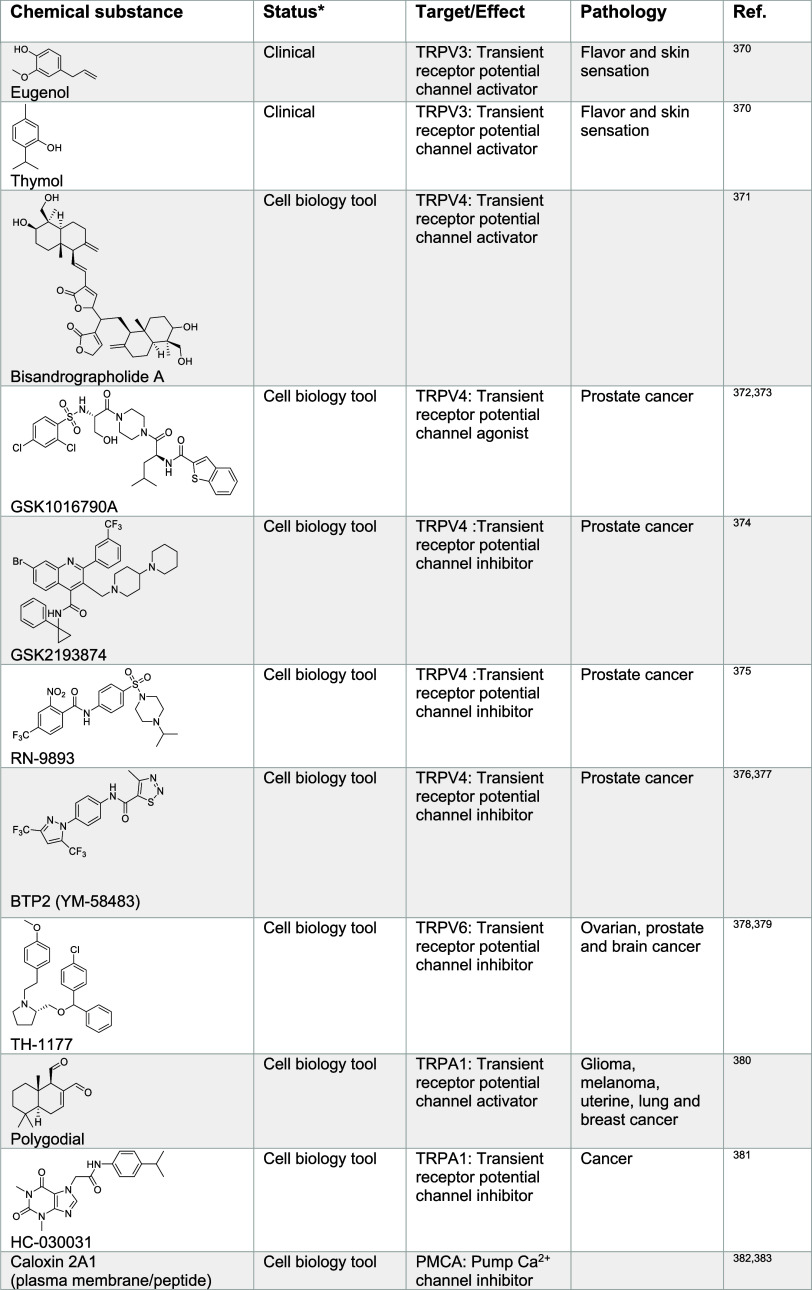

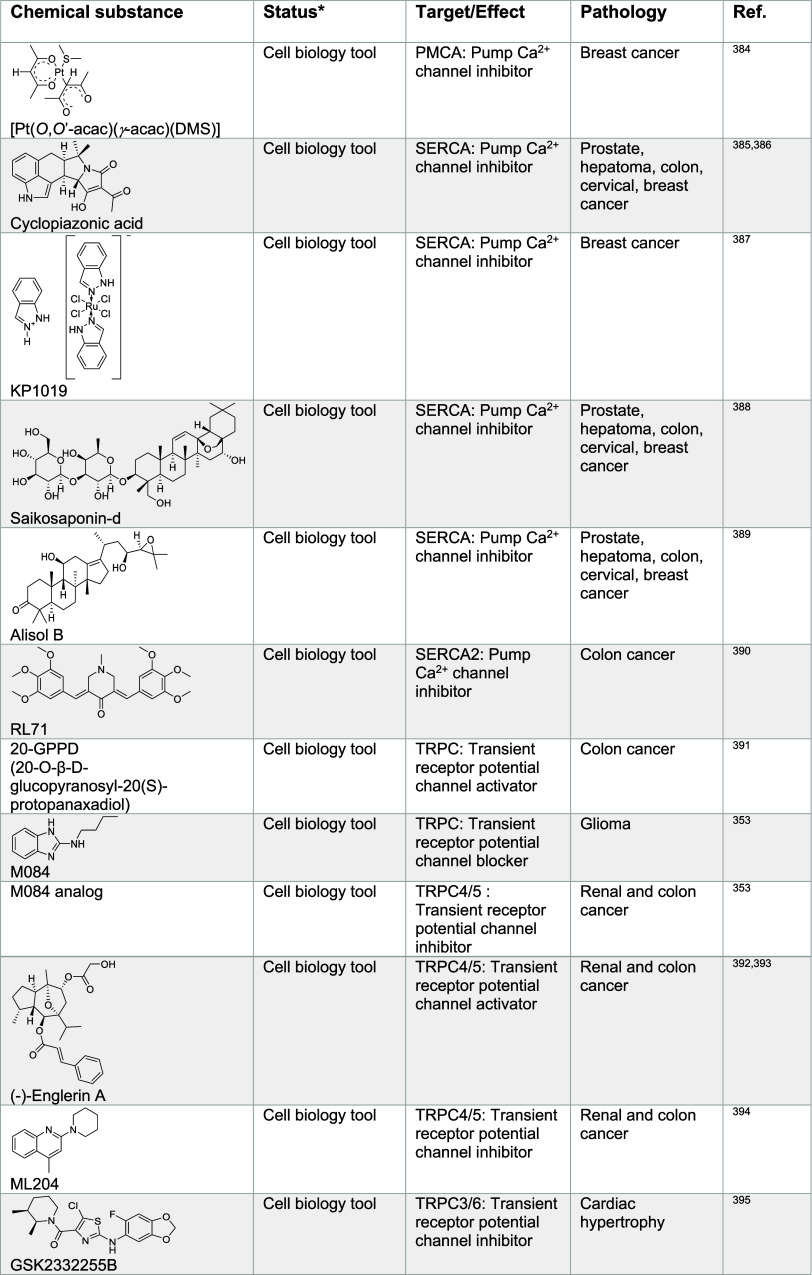

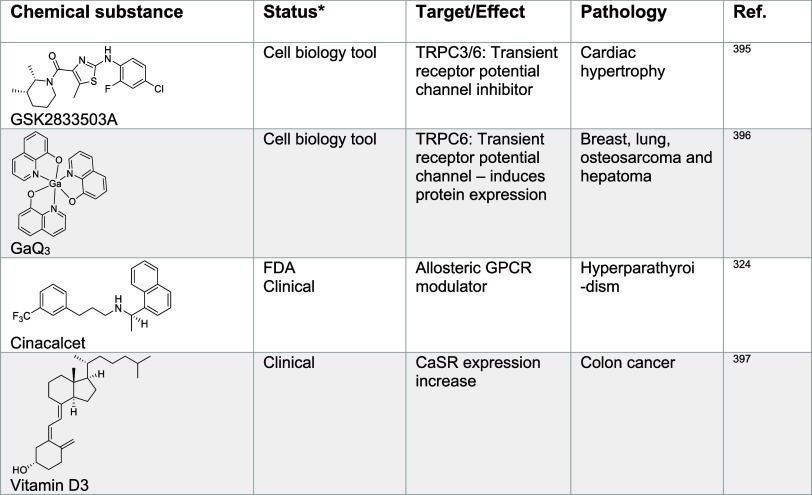
Regulators of Calcium Signaling^24,25,88,258,260−270,274−306,311,312,314−317,322−325,330,341,344−397^

* https://www.fda.gov, https://clinicaltrials.gov

##### 3.2.3.2 Calcium Signaling in Immunity and Inflammation

Calcium signaling regulates NFAT transcriptional programs and T cell activation.^25^ Additionally, calcium levels were found to be elevated in inflammatory macrophages^13^ and lysosomal calcium trafficking has been shown to regulate dendritic cell migration,^309,310^ further supporting the central role of calcium in immunity. The small molecules FK-506 and cyclosporin A (Table 4) form complexes with distinct immunophilins, including FK-506-binding protein and cyclosporin-binding cyclophilin, respectively, susceptible to inhibit the calcium- and calmodulin-dependent phosphatase calcineurin. This in turn prevents NFAT dephosphorylation and translocation to the nucleus inhibiting associated transcriptional programs underpinning activated T cells.^25,311−313^ Since NFAT signaling plays key roles in immune cell activation, the small molecule FK-506 has been exploited for the treatment of eczema and psoriasis, whereas the related natural macrolide rapamycin is used to treat lymphangioleiomyomatosis,^314−316^ a rare systemic disease that results in the destruction of lung tissues. These compounds are also used in the context of organ transplants to prevent immune responses and organ rejection.^317−320^ Calcineurin has been shown to play a role in macrophages.^321^ Since calcium levels directly control the NFAT phosphorylation status, manipulating calcium levels can provide control over cell plasticity in immune cells, with major implications in settings such as autoimmune diseases and acute inflammation. Small molecules that can interfere with calcium signaling, either acting through direct calcium binding or via the targeting of other calcium-signaling effectors, including verapamil,^281^ ghrelin,^288^ mibefradil,^322^ 2-[2-oxo-2-(2,2,4-trimethyl-3,4-dihydroquinolin-1-yl)ethyl]isoindole-1,3-dione (ML-SA1),^323^ cinacalcet,^324^ and others, are listed in Table 4.

Calcium oxalate can induce inflammation in the kidney via a signaling that triggers secretion of interleukin-1β (IL-1β).^325^ Studies on phagocytes showed that the calcium binding proteins S100 are markers of inflammation, potentially linking inflammatory genes and ultimately the expression of inflammatory proteins to calcium levels.^326^ RyR are also involved in T cell activation via hematological and neurological expressed 1-like protein (HN1L).^327^ The so-called calcium microdomains form at very early stages of T cell activation and in neuronal cell signaling. These domains enable the entry of calcium into the cytosol and are key elements of calcium signaling.^328^ In T cells, the two cation channels P2X4 and P2X7 are involved in calcium signaling, controlling calcium flux and T cell activation.^329^ Furthermore, nicotinic acid adenine dinucleotide phosphate (NAADP) is structurally related to IP_3_, which can bind to and open calcium channels inside the cell. The small molecule trans-Ned 19 (Table 4), an antagonist of NAADP-signaling, has been reported to influence T cell plasticity in a mouse model of intestinal inflammation, opening up potential therapeutic opportunities for the treatment of inflammation, including inflammatory bowel disease.^330^ Taken together, this body of work suggests that modulating calcium uptake and signaling in T cells and other immune cells can have a profound effect on cell plasticity and can be exploited therapeutically in various disease settings by targeting calcium homeostasis regulator proteins, downstream signaling effectors, or calcium itself.

##### 3.2.3.3 Calcium Signaling in Neurological Disorders

Calcium is an important signaling effector connecting neurons in synapses via voltage-gated calcium channels among others.^331^ It is involved in the transmission of the depolarizing signal in synapses and thus plays a key role in information exchange between neurons.^332−335^ In addition, calcium has been shown to regulate gene expression, including genes that encode for neurotransmitter receptors in neurons by stimulating CREB-mediated transcription.^336^ This indicates that calcium plays a dual role, influencing action potentials and controlling the underlying expression of neuronal signal transduction transmitters. Specific neurons, such as GABAergic neurons express high levels of the calcium-binding protein parvalbumin, which acts as a calcium buffer.^337^ Cells can modulate signaling via proteins that sequester calcium ions. Changes in calcium levels have been observed in aging nervous systems and correlations with neurodegenerative diseases have been documented.^338,339^ Variations of calcium channel expression levels have been observed at the cell membrane, mitochondria and the ER during aging and in neurological disorders, including Alzheimer’s disease (AD) and Huntington’s disease (HD).^340^ In a cellular model, it was shown that mutated versions of the protein Huntingtin can increase calcium flux in cells.^341^ ER-mediated calcium signaling is a key determinant during neural cell plasticity affecting their dendrites. In addition, changes of calcium homeostasis of the ER have also been observed in AD and a functional link remains to be elucidated.^342,343^ Penfluridol has been successfully used to block T-type voltage-gated calcium channels in academic research, providing new insights for the development of drugs for clinical applications.^284^ The related T-type voltage-gated calcium channel inhibitor pimozide (Table 4) has been developed for the treatment of neurological disorders including epilepsy.^284^

## 4 d-Block Metal Ion Signaling

### 4.1 Manganese Signaling

#### 4.1.1 Regulation of Manganese Homeostasis

**Figure 7:**
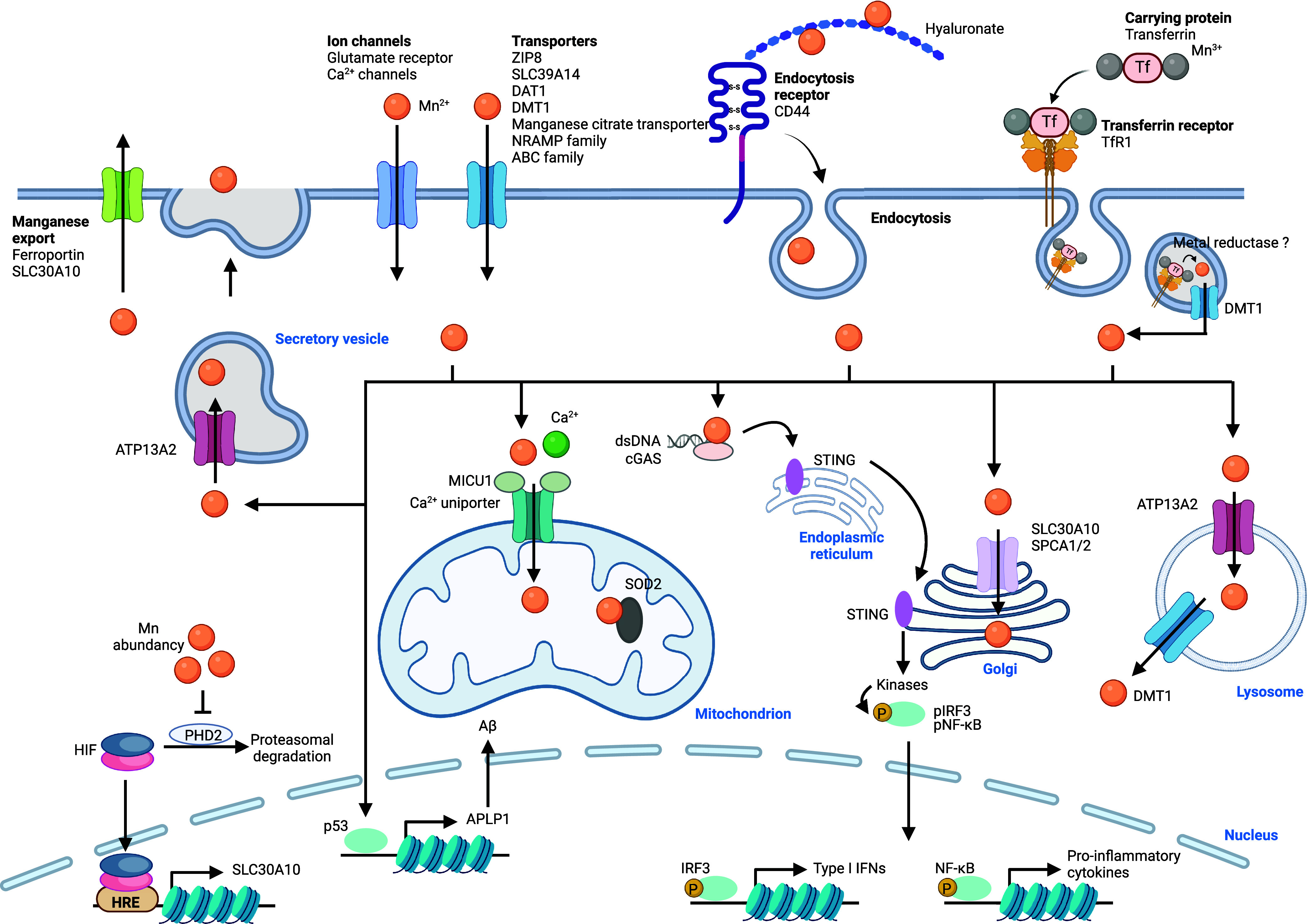
Manganese signaling. Manganese is taken up into the cell via ion channels, transporters, or by means of CD44/hyaluronan- or TfR1/Tf-mediated endocytosis. Intracellular manganese is trafficking to lysosomes, mitochondria, and the Golgi apparatus. The manganese-dependent superoxide dismutase SOD2 is located in mitochondria and consumes radical superoxide protecting cells against oxidative stress. Manganese has been shown to activate cGAS, which in turn activates STING in the ER, promoting STING migration to the Golgi apparatus. Activated STING recruits kinases, which phosphorylates NF-κB and IRF3, allowing transcriptional activation and expression of specific inflammatory genes. Manganese promotes the expression of p53 and its targeted genes. Manganese is exported outside of the cell by ferroportin, SLC30A10, or secretory vesicles. See Abbreviations. Figure generated with BioRender.com.

Manganese is found in the +2 and +3 oxidation states in the cell and can participate in redox reactions. Concentrations of manganese in cells are tightly regulated, and manganese overload can lead to a poisoning condition known as manganism.^398^ This metal exhibits neurotoxic effects upon excessive exposure, and it has been associated with neurodegenerative diseases such as AD and Parkinson’s disease (PD).^399−401^ Several cellular uptake mechanisms have been described (Figure 7). Ion channels enabling manganese transport through lipid membranes have been reported, including ZRT/IRT-like protein 8 (ZIP8), SLC39A14, calcium channels, and dopamine transporter 1 (DAT1) among others.^402−406^ Furthermore, it has been shown that manganese can be taken up by CD44/hyaluronan in macrophages,^13^ and also forms a complex with transferrin (Tf) that can be internalized by transferrin receptor (TfR1) via a mechanism akin to that involving iron and apotransferrin.^407−409^ Upon endocytosis and reduction of manganese(III), manganese(II) can be translocated from late endosomes into the cytosol by DMT1.^402^ Since DMT1 can transport manganese(II), it can also potentially mediate the import of manganese directly into the cell when localized at the plasma membrane, as reported for enterocytes. How manganese(III) is reduced in endosomes remains poorly understood. It is conceivable that metal reductases such as 6-transmembrane epithelial antigen of prostate (STEAP) mediate this reaction.^408^ Alternatively, since manganese(III) can easily capture electrons when subjected to the reducing environment of the cell,^410^ notably in the presence of glutathione (GSH), specific metal reductases may not be involved.

Manganese is transported into various organelles within the cell via specialized transporters including SLC30A10 and SPCA1/2 that drive manganese to the Golgi apparatus, and ATP13A2, which regulates manganese translocation into lysosomes and excretory vesicles^411^ (Figure 7). The calcium uniporter situated in mitochondrial membranes can transport both calcium and manganese into the mitochondria. Interestingly, mitochondrial calcium uptake 1 protein (MICU1) interacts with the calcium uniporter to control its ability to translocate divalent metals. MICU1 contains a metal binding site, and calcium binding has been shown to elicit a conformational change of MICU1, which enables transport of divalent metals by the calcium uniporter.^412^ Manganese, however, can also interact with the metal binding site of MICU1, but it does not cause the same conformational change, leading to an inactivation of the calcium uniporter. Thus, when excess calcium is present, both calcium and manganese can be transported into mitochondria via this transporter, whereas excess manganese inhibits metal transport.^413^ SLC30A10 can also localize at the cell membrane and mediate the export of manganese. Interestingly, the iron export protein ferroportin has also been shown to control the cellular export of manganese.^414^ The expression of SLC30A10 is controlled by hypoxia-inducible factors (HIFs), and its expression is increased upon elevated manganese levels to prevent poisoning by this metal.^415^ Recent work has documented that proline hydroxylase 2 (PHD2) can be inactivated by manganese replacing reactive iron(II) when manganese levels are high. Since PHD enzymes control proline hydroxylation of HIFs, which leads to HIF degradation, inactivation of these enzymes leads to elevated HIF and SLC30A10 levels. Thus, PHD2 could be defined as an intracellular manganese sensor.^416^ Manganese homeostasis has traditionally been difficult to dissect due to the lack of adequate biological or chemical tools. Recently, cell-permeable fluorescent manganese(II)-specific probes have been developed to dissect manganese homeostasis,^417^ identifying the Golgi apparatus as a manganese storage site.^418^

#### 4.1.2 Cellular Functions of Manganese

In mitochondria, manganese is part of the active site of the mitochondrial enzyme superoxide dismutase 2 (SOD2).^419^ Both the +2 and +3 oxidation states of the metal are involved in the disproportionation of superoxide radical.^420^ Manganese has been found to be upregulated in mitochondria of inflammatory macrophages.^13^ The production of hydrogen peroxide by SOD2 was found to be crucial for the interconversion of NADH into nicotinamide adenine dinucleotide (NAD^+^), enabling the production of metabolites required for the epigenetic regulation of transcriptional programs underlying acquisition of a pro-inflammatory phenotype. cGAS is a cytosolic DNA sensor that plays a crucial role in host defense against viral infections or genotoxic stress.^421^ Interestingly, manganese can activate monomeric cGAS, which positively couples with double-stranded (ds) DNA-dependent activation, as manganese(II) enhances the detection sensitivity of cGAS for dsDNA, thereby promoting its enzymatic activity.^422^ In addition, manganese(II) can activate cGAS independently of DNA.^423^ Activation of STING in turn recruits kinases, which phosphorylate the nuclear factor kappa-light-chain-enhancer of activated B cells (NF-κB) and interferon regulatory factor 3 (IRF3), regulating the expression of inflammatory genes in immune cells.

#### 4.1.3 Manganese Signaling and Diseases

##### 4.1.3.1 Manganese Signaling in Cancer

Acquisition of a pro-metastatic cancer cell state has been shown to require a metabolic reprogramming similar to that found in immune cells for activation,^13^ supporting a role of manganese in cancer metastasis.^424^ Interestingly, increases of manganese levels are associated with metastatic potential in tumors.^425^ SOD2 mimetics, such as the manganese complex-forming small molecule M40403 (Table 5), have been shown to exhibit anticancer activity as well as effects on immune responses.^426^ SOD2-dependent increase of hydrogen peroxide has been shown to promote ERK1/2 signaling and transcriptional changes impacting metalloproteinase expression.^427^ Interestingly, iron can substitute manganese in SOD2 under iron-high and manganese-low conditions. This was shown to alter SOD2 activity, breaking down hydrogen peroxide and causing the production of reactive oxygen species (ROS) instead.^428^ Thus, alterations in the manganese/iron balance can profoundly impact the biology of the cell. cGAS-STING is also involved in immune sensing in the context of cancer.^429^ For instance, it was reported that manganese enhances cancer detection by the innate immune system.^430^ Indeed, manganese-loaded nanoparticles enhance cGAS-STING activation, which holds great potential for cancer immunotherapy.^431^ The cytokine interleukin-2 (IL-2) is used to treat malignant melanoma and metastatic renal cell carcinoma. However, IL-2 treatment can also cause hypertension, which limits its use. The SOD mimetic M40403 can reduce hypotension caused by IL-2, allowing for an increase of IL-2 administration in mice and enhancing its anticancer activity.^426^

##### 4.1.3.2 Manganese Signaling in Immunity and Inflammation

Manganese increases the innate immune response in lipopolysaccharide-induced sepsis.^432^ Since manganese levels can affect the efficacy of the cGAS-STING pathway, manganese plays an integral role in host defense against viral infections.^433^ Targeting manganese signaling represents a potential therapeutic strategy in inflammatory diseases, aging-related inflammation, and neurodegeneration.^421,434^ Manipulating manganese homeostasis is a tractable strategy to control the innate immune response in infectious diseases and cancer.^421,429^ In line with these studies, in vitro experiments have revealed that manganese treatment leads to an increase of the expression of genes involved in the innate immune response, which is consistent with the role of manganese in the maintenance of NAD^+^ and the epigenetic control of transcriptional programs underlying inflammation.^13,435^

##### 4.1.3.3 Manganese Signaling in Neurological Disorders

It has been shown that mutations of the cellular manganese importer SLC39A14 can lead to impairment of manganese transport and childhood-onset parkinsonism-dystonia,^436^ highlighting the role of manganese in brain development and neuronal plasticity. Upon increased exposure to manganese during development, manganese can accumulate in the basal ganglia and cause manganism, conferring a PD-like syndrome.^437^ Manganese chelators, such as triethylenetetramine hexaacetic acid (TTHA), DTPA and *N*,*N*′-di(2-hydroxybenzyl)ethylenediamine-*N*,*N*′-diacetic acid monohydrochloride hydrate (HBED)^438,439^ have been studied to treat manganism and manganese-induced neurotoxicity (Table 5). However, these small molecules can also chelate other metal ions and cause side effects.

**Table 5:**
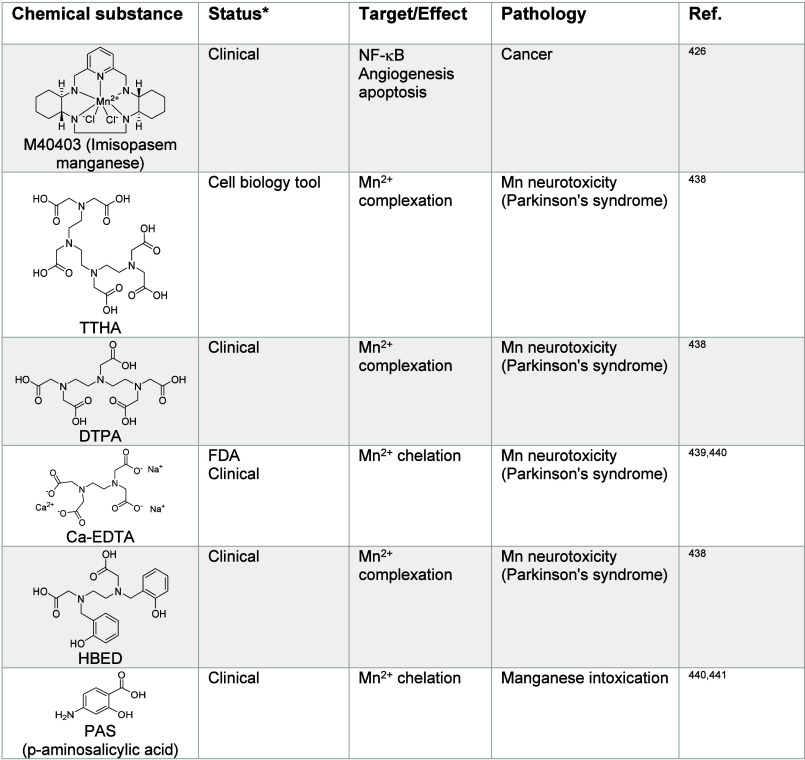
Regulators of Manganese Signaling^426,438−441^

* https://www.fda.gov, https://clinicaltrials.gov

### 4.2 Iron Signaling

#### 4.2.1 Regulation of Iron Homeostasis

**Figure 8:**
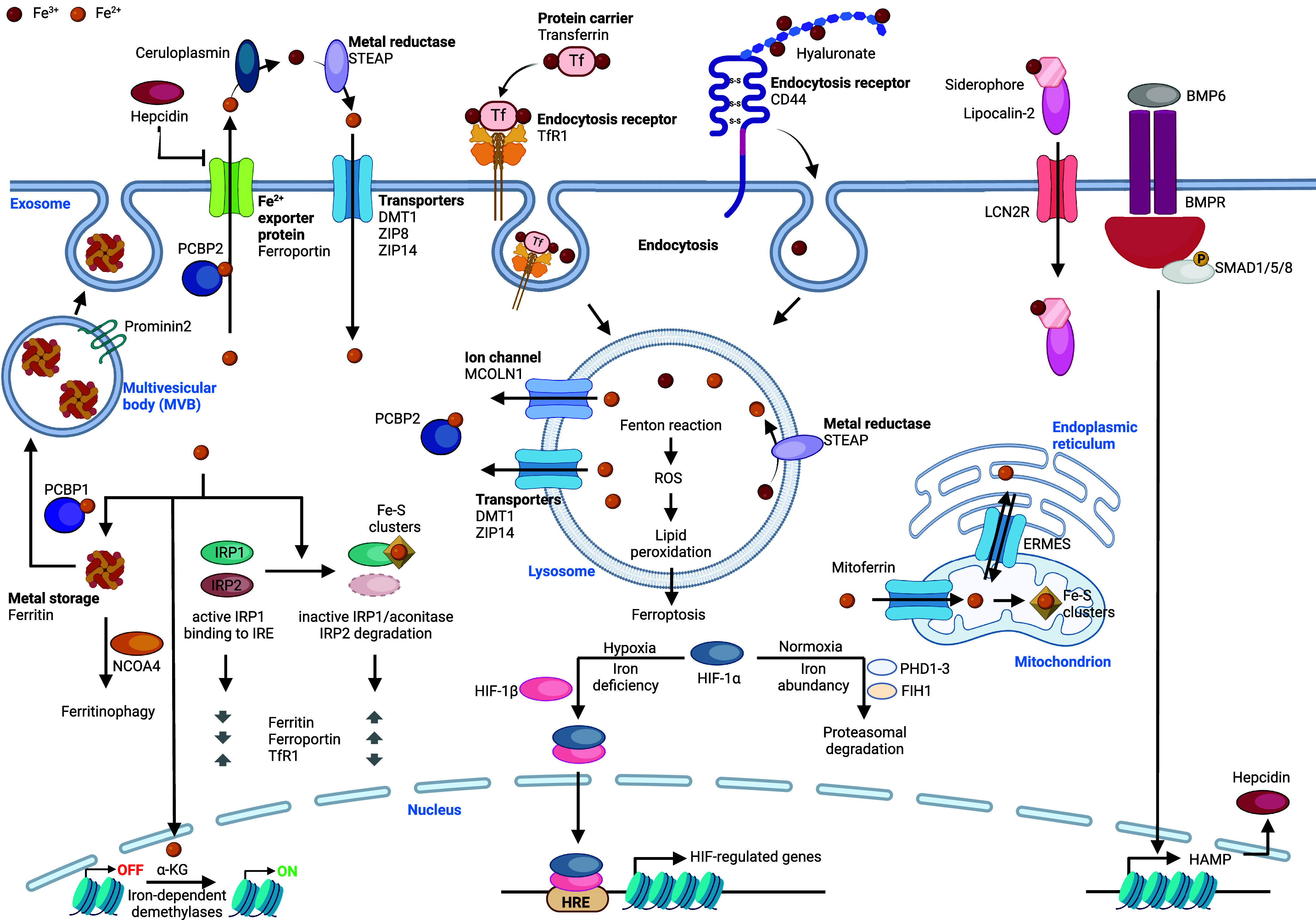
Iron signaling. Iron(II) is taken up into the cell via transporters and iron(III) by means of CD44/hyaluronan- and TfR1/Tf-mediated endocytosis. Specific chaperones (PCBP1 and PCBP2) bind to cytosolic iron and contribute to the trafficking of iron to specific organelles. In lysosomes, iron promotes Fenton-type chemistry, producing ROS, lipid peroxidation, and triggering ferroptosis. Mitoferrin transfers cytosolic iron to the mitochondrial matrix. In mitochondria, iron is used for the formation of iron–sulfur clusters in the ETC. In the nucleus, iron is the catalyst of α-KG-dependent demethylases, controlling the epigenetic landscape and transcriptional programs. Under iron-deficient conditions/hypoxia, HIF-1α and HIF-1β are stable, form a heterodimer, bind to HRE, and function as a TF. In the presence of iron and under normoxia, HIF-1α is hydroxylated by PHD1–3 and FIH1, leading to proteasomal degradation of HIF-1α. Iron is stored via intracellular protein complex ferritin. The cargo receptor NCOA4 mediates autophagic ferritin degradation, which contributes to the control of levels of available iron within the cell. Iron regulates the binding of IRP1 and IRP2 to IRE in UTRs. IRP1 can incorporate an iron–sulfur cluster, leading to an inactive form that cannot bind to IREs. In the presence of high iron, the IRE/IRP regulatory system promotes increase of ferritin and ferroportin and decrease of TfR1. Iron trafficking to the ER requires ERMES, directly linking the ER with the mitochondria for iron exchange. Iron is exported outside the cell via ferroportin, a mechanism that can be blocked by the hormone hepcidin. The BMP/SMAD pathway regulates hepcidin expression in an iron-dependent manner. Lipocalin-2 binds to siderophore 2,5-DHBA, reducing free iron levels. Prominin2 can promote the formation of ferritin-containing MVBs and exosomes, which can export cellular iron. See Abbreviations. Figure generated with BioRender.com.

The regulation of cellular iron homeostasis is complex.^442^ In the cell, iron is mainly found as +2 and +3 oxidation states as well as a crystalline form of iron oxide. Unlike alkali and alkaline earth metal ions, iron is redox active under physiological conditions. It can form complexes with proteins, such as Tf and ferritin, glycans, such as hyaluronan, or is found as a labile iron pool, i.e., free species. Cellular iron homeostasis has been thoroughly documented with the discoveries of import, traffic, storage, and export mechanisms, where iron is involved in the regulation of many biological processes^442,443^ (Figure 8). Cellular iron uptake can be mediated by several mechanisms, including the Tf/TfR1 mechanism. Tf binds to two iron(III) ions (holo-transferrin) and this complex becomes a competent binding partner of TfR1, which is internalized via a clathrin-dependent endocytosis mechanism.^444^ TfR1 then recycles back to the cell surface. Iron can also be imported into cells via transporters, such as ZIP14, ZIP8, or DMT1. For example, DMT1 is found on the cell membrane of enterocytes, where it mediates transport of iron into the cell. Tf-mediated cellular iron import has long been considered to be the main mechanism of iron endocytosis. The literature, however, refers to two distinct pools of iron in the blood: a pool that is Tf bound and another that is not transferrin-bound. In cancer and immune cells acquiring a distinct identity, i.e., cell-state transition,^445^ CD44 mediates endocytosis of iron(III)-bound hyaluronan,^446^ via a clathrin-independent endocytosis mechanism.^447^ There is a prevalence of metal binders in blood, plasma, and the extracellular space. The chemical composition of diseased tissues has been shown to be distinct from that of healthy tissues, for example, with the abundance of hyaluronan in the stroma of pancreatic tumors and in the lung of severe COVID-19 patients,^448,449^ which argues for the prevalence of CD44-mediated iron uptake over other mechanisms, and this plays a role in the control of cell identity by iron in cancer and inflammation.^446,450,451^

Importantly, intracellular iron regulates the function of iron regulatory protein 1 (IRP1) and IRP2, which are RNA-binding proteins.^452^ IRP1 is the cytosolic isoform of the mitochondrial aconitase. Iron regulates the binding of IRPs to specific RNA stem-loop structures, called iron-responsive element (IRE), to the 5′- and 3′-untranslated regions (UTRs) of various mRNAs including those encoding TfR1 and ferritin. Interestingly, IRP1 can form an iron–sulfur cluster that prevents its binding to IREs impacting translation. For example, when iron levels are low, IRP1 can bind to multiple IREs in the 3′-UTR of TfR1, stabilizing the mRNA transcript and promoting protein biosynthesis. In contrast, binding of IRP1 to the 5′-UTR of ferritin inhibits translation.^453^ Thus, while expression of TfR1 is reduced upon increase of cellular iron, expression of the iron storage protein ferritin is increased upon iron uptake.^454,455^ This iron signaling mechanism allows the cell to maintain low basal levels of iron, reducing endocytosis and increasing storage to avoid oxidative stress when the iron demand in the cell is moderate. In contrast, CD44 expression is unlocked by iron at the epigenetic and transcriptional levels, allowing cells to transiently upregulate the iron content when the demand is higher, for instance to enable rapid acquisition of a distinct cell state independently of genetic alterations, such as epithelial-to-mesenchymal transition (EMT) in cancer^446^ (Figure 8).

Upon endocytosis, iron(III) is reduced by specific metal reductases, including the STEAP family of proteins.^456^ Iron(II) is then exported from the lumen to the cytosol via DMT1, where it is transferred to the iron chaperone PCBP1 and PCBP2.^457^ Iron is stored in the cell, forming a complex supramolecular structure with ferritin. Ferritin can form a multimeric complex composed of 24 subunits of ferritin heavy chain (FTH1) and ferritin light chain (FTL).^458^ FTH1 exhibits intrinsic ferroxidase activity, allowing oxidation and storage of iron as a crystalline redox-inactive form in the inner core of the ferritin multimer. Iron can be released from ferritin through ferritinophagy, a mechanism that targets ferritin for degradation.^459^ In this process, ferritin shuttles to autophagosomes via nuclear receptor coactivator 4 (NCOA4),^460^ where it is targeted for proteolysis in autolysosomes. NCOA4 is a key regulator of the available iron in the cell. Thus, interfering with ferritinophagy can impact DNA replication and induce cell proliferation defects as well as other processes reliant on iron chemistry.^461^

Regulation of the subcellular distribution of iron in the cell is critical and remains incompletely understood. For example, mitoferrins have been shown to mediate transport of iron to the mitochondria.^462^ Iron trafficking to the ER is more complex, as it requires the endoplasmic reticulum mitochondria encounter structure (ERMES), directly linking the ER with the mitochondria for iron exchange.^463^ Transport and regulation to and from other organelles are less documented. For instance, it is not yet clear how iron shuttles to the cell nucleus, a cellular compartment where iron controls key processes including epigenetic regulation and transcription.

Iron export from the cell is mediated via ferroportin,^464^ which receives iron from PCBP2.^465^ Interestingly, ferroportin can be blocked extracellularly by the hormone hepcidin,^466,467^ providing another level of control of cellular iron levels. Hepcidin itself is regulated by bone morphogenetic protein 6 (BMP6), which is also regulated by iron levels via the TF nuclear factor erythroid 2-related factor 2 (NRF2).^468^ Furthermore, iron-containing ferritin can be exported from cells.^469^ In line with this, Prominin2 has been implicated in the regulation of lipid dynamics and can promote the formation of ferritin-containing multivesicular bodies (MVBs) and exosomes to export cellular iron from mammary epithelial and breast carcinoma cells. This adaptative mechanism has been shown to protect cells against ferroptosis.^470^

#### 4.2.2 Cellular Functions of Iron

Iron is a major functional component of hemoglobin, the metalloprotein in erythrocytes that enables oxygen transport in most animals.^471^ Heme comprises a complex of iron(II) tetracoordinated within a tetrapyrrole ring. Hemoglobin can reversibly bind to molecular oxygen and carbon dioxide in a pH-dependent manner. Thus, with the appropriate chemical environment, iron can fix and transport oxygen from the lungs to other tissues, enabling cellular respiration and transporting carbon dioxide back from cells for clearance.

Hypoxia-inducible transcription factors (HIFs) are TFs that respond to cellular oxygen levels.^472,473^ Interestingly, many gene targets of HIF are involved in the regulation of iron homeostasis.^474^ HIF is regulated by iron-dependent proline dioxygenase proteins that use iron and molecular oxygen to promote HIF hydroxylation.^475,476^ This enables binding of von Hippel–Lindau tumor suppressor (pVHL), which exhibits E3 ubiquitin ligase activity, leading to ubiquitination and proteasomal degradation of HIF. Interestingly, iron chelation and treatment with cobalt(II) ions inhibit pVHL binding and mimic hypoxia. Under hypoxic conditions, HIF is stable and regulates a specific transcriptional program.^477,478^ Thus, iron can act as a transcriptional repressor. Interestingly, besides its function in oxygen transport, heme itself can act as a cell signaling molecule,^479^ impacting on the activity of TFs^480−482^ and kinases.^483^ Cellular import and export of heme are regulated by a complex protein network.^484^

Iron is required for many other cellular and physiological processes, including erythropoiesis, immune function, mitochondrial respiration, nucleotide biosynthesis, DNA repair, DNA replication, and telomere maintenance.^485,486^ For instance, iron is a key component of iron–sulfur clusters found in proteins of the ETC and can act as a cofactor of specific enzymes in the cell. In mitochondria, iron controls cell metabolism, notably with the production of key metabolites required for the control of the epigenome and cell identity.^446,487^ This includes α-ketoglutarate (α-KG), a metabolite produced downstream of the iron–sulfur cluster-containing aconitase, an enzyme of the Krebs cycle that uses iron as a cofactor to mediate its activity. Specific demethylases of histones and nucleic acids exploit α-KG and molecular oxygen as cosubstrates of demethylation. Iron acts as a rate-limiting catalyst of these demethylases, which include histone demethylases of the Jumonji family, ten-11 translocation (TET) enzymes, fat mass and obesity-associated protein (FTO)^488^ and AlkB homologue 5^489^ that target methylcytosine and *N*^6^-methyladenosine, respectively.^490−493^ Thus, in contrast to its role as suppressor of HIF-related transcriptional programs, iron also mediates cell signaling by unlocking other transcriptional programs underlying epigenetic and epitranscriptomic control of cell identity in cancer and immunity.^446^

#### 4.2.3 Iron Signaling and Diseases

Imbalances in iron homeostasis can cause diseases.^494^ Iron-overload diseases, such as hereditary hemochromatosis, are characterized by tissue damage and fibrosis, which can lead to organ failure.^495^ In contrast, iron-deficiencies, also called anemia, can lead to poor oxygenation of tissues.^496^ Several strategies have been established to treat anemia-related diseases including supplementation with iron salts. Moreover, thalassemias make up a group of genetic diseases characterized by the production of abnormal hemoglobin. The most frequent is β-thalassemia,^497−500^ where mutations of β-globin have been reported. Standard-of-care treatments include regular blood transfusions. Alternative therapeutic strategies that can restore or improve hemoglobin function have been explored.^501−503^ Luspatercept is a recombinant fusion protein derived from activin receptor type II B that can bind to transforming growth factor (TGF) and reduce the suppressor of mothers against decapentaplegic (SMAD) signaling. This in turn leads to improved erythroid cell maturation.^500^ Accumulation of pulmonary iron has been associated with lung dysfunction. In contrast, HIF dysregulation has been observed in anemia caused by chronic kidney disease, which can be treated with HIF proline dioxygenase inhibitors such as daprodustat, vadadustat, molidustat, and desidustat (Table 6).^504^

**Table 6:**
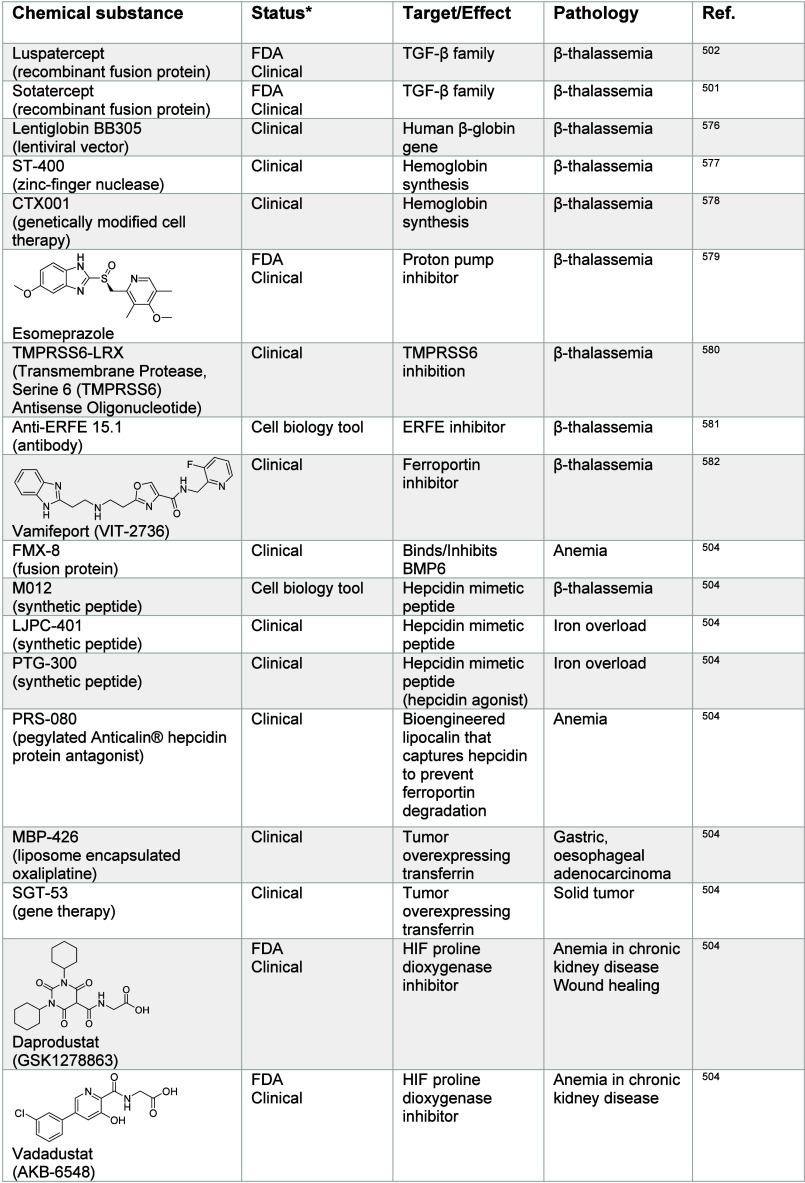

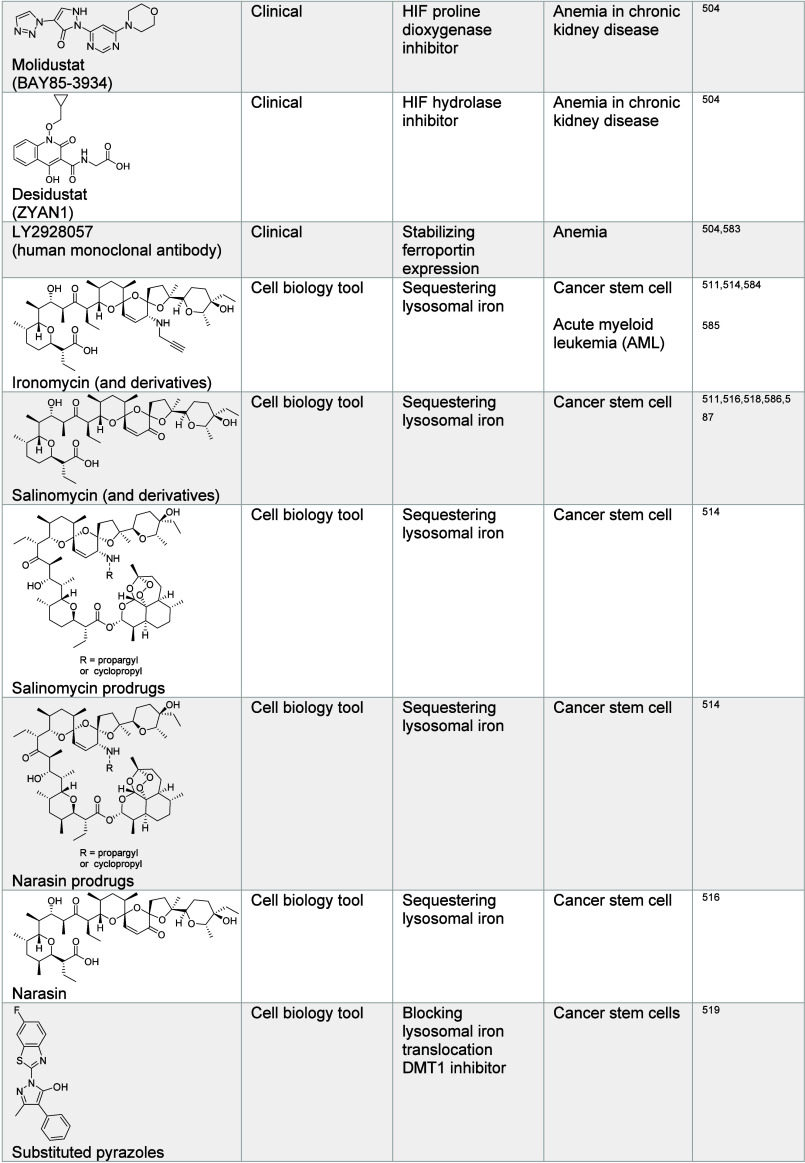

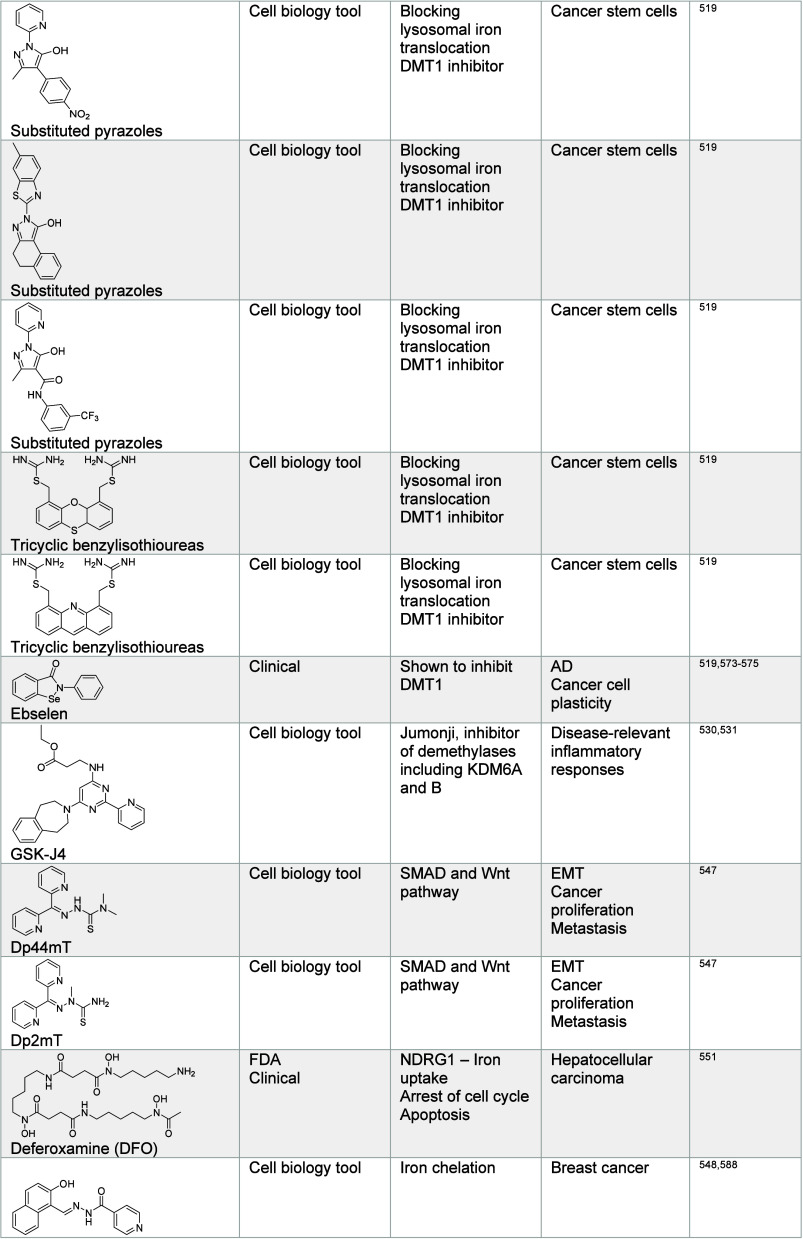

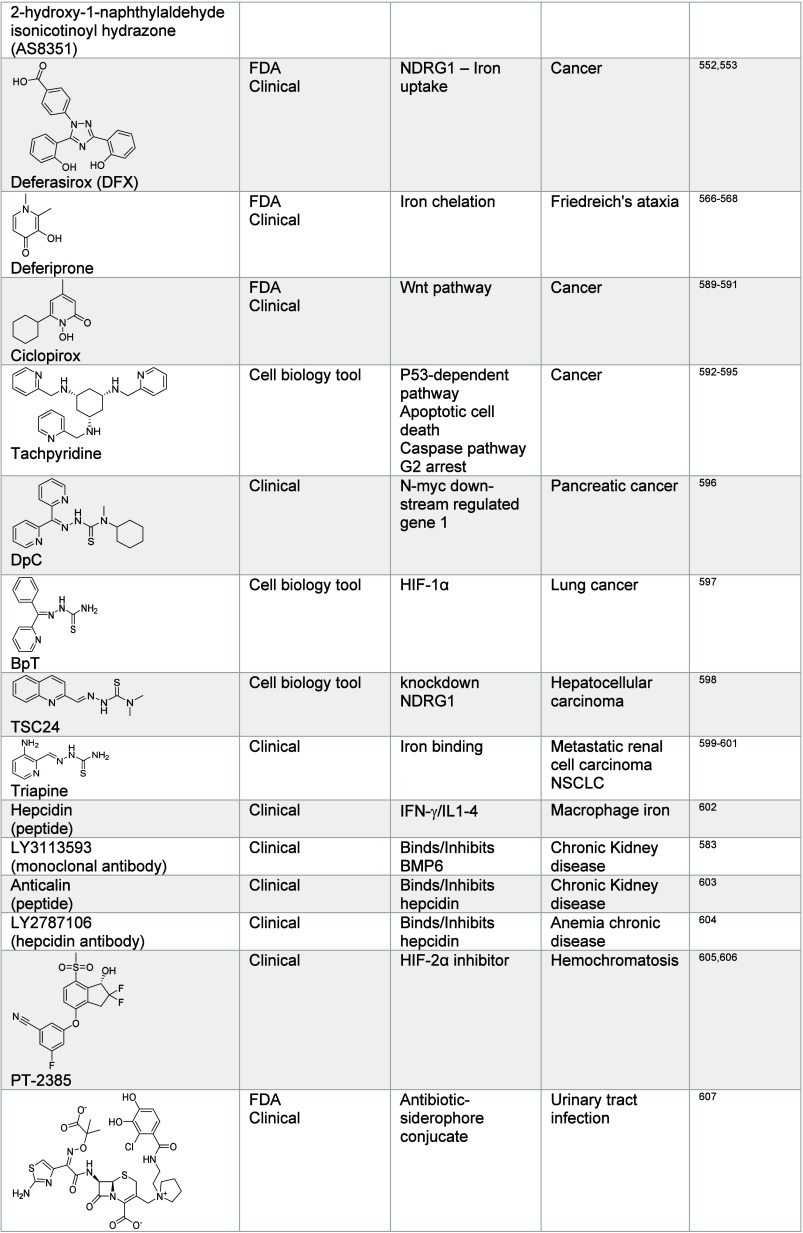

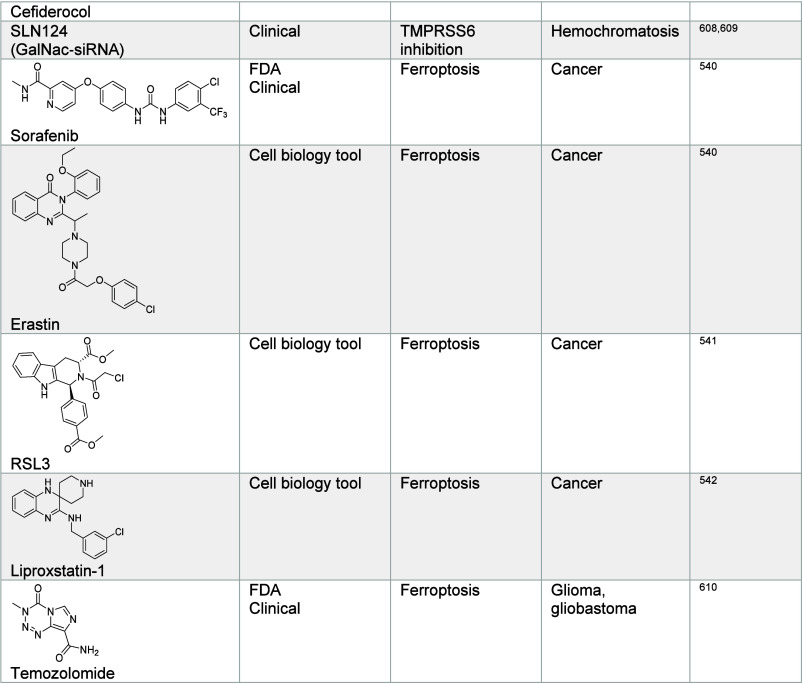
Regulators of Iron Signaling^501,502,504,511,514,516,518,519,530,531,540−542,547,548,551−553,566−568,573−610^

* https://www.fda.gov, https://clinicaltrials.gov

The tight regulation of free iron in the blood also plays a role in bacterial infection. Indeed, bacteria can secrete siderophores to sequester iron, which is essential for their growth. These include the plague-causing pathogen *Yersinia pestis*, among others.^505^ In humans, macrophages secrete lipocalin-2 (LCN2) upon stimulation with Toll-like receptors (TLR), which sequesters bacterial siderophores, limiting iron supply to bacteria.^506^

##### 4.2.3.1 Iron Signaling in Cancer

Changes in iron metabolism fuel cancer progression on several levels, including cell proliferation, cancer cell invasion, and metastasis formation, as well as cancer recurrence and drug resistance.^507,508^ Iron is required for fundamental cellular processes by healthy cells, cancer cells, and importantly also by other cells comprising the tumor environment, such as cancer-associated fibroblasts, immune cells, endothelial cells, pericytes, and others. Thus, different cell types may compete for iron uptake. For instance, in renal cell carcinoma, it has been shown that macrophages of the tumor microenvironment secrete and supply iron to cancer cells.^509^ This is reminiscent of metabolic competition in the tumor microenvironment that drives cancer progression.^510^ Thus, the iron supply to the tumor impacts cancer progression.

Increase of cellular iron characterizes cancer cells in the drug-tolerant persister (DTP) state, which has been linked to cancer metastasis, relapse, and poor clinical outcomes.^511−513^ This feature has previously been exploited in preclinical models of cancer refractory to standard-of-care treatments, where small molecule-induced iron retention in lysosomes leads to a buildup of ROS in lysosomes, causing membrane lipid oxidation and ferroptosis.^511,514^ These studies highlight a potential therapeutic strategy for the clinical management of cancer.^515^ The natural product salinomycin has been identified as a potent eradicator of breast cancer stem cells (CSCs).^516^ Since salinomycin can act as a ionophore capable of transporting alkali metals across lipid bilayers,^517^ a MoA involving sodium/potassium transport had been evoked. However, the more potent synthetic derivative ironomycin was found to be effective against CSCs at concentrations that did not substantially alter alkali metal levels, indicating that other mechanisms may be at play in this context.^511^ Notably, it was found that the MoA of this class of compounds involves direct binding to iron(II) in lysosomes, interfering with iron translocation from the lumen to the cytosol and causing oxidative damage to lipid membranes in this organelle.^511,518^ In support to this MoA, DMT1 inhibitors, such as substituted pyrazoles and benzylisothioureas (Table 6), can block iron transport from lysosomes, leading to an increase of lysosomal iron and inducing death in cancer cells that had undergone an EMT program, a cell transformation that can confer stem-like properties and tolerance to standard-of-care treatments.^519^ The endolysosomal compartment plays a central role in the regulation of cellular iron homeostasis.^520^ Thus, lysosomal iron can be exploited for small molecule intervention. In line with this, iron-activatable prodrugs with a propensity to accumulate in lysosomes were designed. It was shown that chimeras of salinomycin or the related natural product narasin and the organic endoperoxide artesunate (Table 6), which can be readily cleaved upon exposure to iron in lysosomes, induces oxidative stress and ferroptosis.^514^ Increased iron trafficking was also reported in glioblastoma cancer stem-like cells^512^ and CSCs of ovarian cancer,^513^ thus putting forward the idea that other cancer types that are notoriously difficult to treat may be eradicated by directly manipulating cellular iron homeostasis.^521^ Melanoma cell differentiation is also linked to increased ferroptosis susceptibility,^522^ further supporting the key notion that cell plasticity requires iron, which confers vulnerability to oxidative stress in cancer.

Cancer and immune cells can undergo cell-state transitions via epigenetic reprogramming. To promote the activity of iron-dependent demethylases, it was found that cells upregulate mechanisms of iron import.^13,446^ In these settings, CD44-mediated iron endocytosis prevails over the canonical TfR1 pathway, which enables an increase of iron supply, catalyzing epigenetic changes underlying the acquisition of another cell state. Importantly, CD44 expression is not negatively regulated by iron, unlike TfR1, making it possible for cells to transiently increase the iron load. Following Le Chatelier’s principle,^523^ cells must increase the reagents available at specific chromatin loci to compete against the activity of methyltransferases capable of promoting hypermethylation of key histone and nucleic acid residues, thereby switching off the expression of specific genes and preventing cell state transitions. Changes of cell states underpin many biological processes, such as EMT^11,445^ and noradrenergic-to-mesenchymal transition in cancers,^524^ contributing to cancer metastases and the acquisition of a DTP cancer cell state. Other processes involving cell-state transitions include the activation of immune cells,^13^ including macrophage polarization.^525^ CD44 has been associated with these processes and is a reliable marker of cell plasticity, playing a central role in metal import and thus cell signaling. Targeting CD44 for therapeutic purposes may represent an interesting strategy in this context.^526^ CD44 expression is transcriptionally repressed at the epigenetic level by the histone 3 lysine 9 dimethyl (H3K9me2) mark.^446^ It was shown in triple negative breast cancer cells acquiring a mesenchymal cell state that the expression of CD44 is unlocked by the iron-dependent demethylase plant homeodomain finger protein 8 (PHF8), preferentially targeting H3K9me2.^446^ These studies show that the cell has evolved complementary iron endocytosis regulatory mechanisms enabling maintenance of functional iron levels, which are lower at the basal cell state, as well as an alternative mechanism required to increase the iron load to promote acquisition of a distinct identity. Thus, iron signaling allows for rapid and reversible cell adaptation that is best exemplified by the capacity of cancer cells to acquire drug-resistant states upon treatment with the standard-of-care and macrophages that rapidly adopt specific properties upon exposure to pathogens for clearance. The regulation of epigenomes defines the cell identity. It relies on complex mechanisms, including the well-orchestrated activities of demethylases^527^ and methyltransferases among others that target specific histone residues.^528^ In addition, nonhistone substrates are also targets for iron-dependent demethylases, including DNA and RNA, which can impact the transcriptome, proteome, and cell identity.^491,529^ Inhibitors of iron-dependent histone demethylases, such as GSK-J4 (Table 6), have been developed to inhibit specific demethylases that catalyze the demethylation of key histone marks.^530,531^ The iron-dependent histone lysine demethylase 3B (KDM3B) has been described as a sensor for intracellular iron levels that regulate mammalian target of rapamycin complex 1 (mTORC1) via H3K9me2, thus iron also impacts mTOR signaling.^532^ Manipulating RNA methylation has not yet been exploited with proven therapeutic benefits in clinical settings. Nevertheless, delineating how cellular iron homeostasis globally impacts cell signaling and the acquisition of distinct cell properties holds great promise for therapeutic intervention, despite its complexity. Intriguingly, since cells undergoing cell state transitions require increased levels of iron. The increased amount of this redox-active metal confers vulnerability to oxidative stress and thus to ferroptosis.^511,533,534^ Cells have developed systems to circumvent undesired chemistry mediated by redox-active iron, including glutathione peroxidase 4 (GPX4) and ferroptosis suppressor protein 1 (FSP1) as well as other lipid membrane repair mechanisms.^535−539^ Small molecule activators (for instance erastin^540^ or RSL3^541^ listed in Table 6) or inhibitors (for instance, liproxstatin-1^542^) of ferroptosis represent promising therapeutic strategies in diseases where iron has been implicated, including cancer and neurodegenerative diseases among others.^543,544^ NRF2 is upregulated under conditions of oxidative stress, which regulates the expression of genes that encode proteins involved in the management of oxidative stress.^545^ Interestingly, inhibition of NRF2 has been shown to lead to increased vulnerability to ferroptosis in ovarian cancer.^546^ Manipulating NRF2 activity may thus be exploited to sensitize cancer cells to other ferroptosis-inducing small molecules.

Previous work has shown that the iron chelators deferoxamine (DFO) and Dp44 mT can inhibit EMT.^547^ N-myc downstream regulated 1 (NDRG1) was specifically upregulated in breast cancer cells treated with DFO and 2-hydroxy-1-naphthylaldehyde isonicotinoyl hydrazone (AS8351) (Table 6), and high expression of this gene is associated with reduced tumor growth and metastatic suppression.^548^ It has been shown that TGF-β/SMAD and wingless/integrated (Wnt) signaling pathways were involved in the regulation of EMT proteins. Taken together, this suggests that targeting iron homeostasis or proteins of signaling pathways involved could be exploited in cancer. Other signaling pathways activated in cancer have been shown to be influenced by iron, for instance the MAPK/ERK pathway in neuroblastoma,^549^ where iron-dependent production of ROS impacts calcium signaling by activating a calcium channel.

Interestingly, LCN2 is expressed by cancer cells in the cerebrospinal fluid upon stimulation by macrophages.^550^ It was argued that these cancer cells can outcompete macrophages for iron uptake, promoting cancer growth. Iron chelation therapy with DFO (Table 6) was shown to inhibit cancer cell proliferation. Thus, targeting iron or LCN2 represent promising therapeutic strategies in this context. Indeed, DFO is used in anticancer therapy, for instance in hepatocellular carcinoma.^551^ Other iron chelators are also used as anticancer treatments, including deferasirox (DFX)^552,553^ (Table 6). However, DFX has been shown to localize in mitochondria, whereas DFO has been detected in lysosomes^554^ and the cell nucleus.^446,554^ Thus, therapeutic and off-target effects resulting from iron chelation are not expected to be identical. Additional investigation is needed to delineate the activity of iron chelators in disease-relevant settings. Importantly, about 40% of cancer patients are undergoing anemia, and clinical management can involve iron supplementation.^555^ Little is known about the adverse effect of iron supply on cancer progression, which requires further investigation.

Since HIF-1 has been linked to the regulation of glucose metabolism, angiogenesis, and cancer cell invasion, HIF signaling, which depends on iron homeostasis and levels of molecular oxygen, has been considered a potential target for cancer therapy.^556^ Indeed, many cancers show dysregulated HIF activity. Targeting HIF or HIF-signaling regulators, such as HIF proline dioxygenase,^504^ represents an interesting axis for the development of anticancer strategies.^557^

##### 4.2.3.2 Iron Signaling in Immunity and Inflammation

Increase of iron is associated with macrophage activation in inflammatory settings.^13^ Notably, the iron homeostasis regulators IRP1 and IRP2 play a critical role in neutrophil development, enabling neutrophil differentiation into fully mature neutrophils.^558^ Similarly, iron controls the fate of hematopoietic stem cells acting at the chromatin level, which is molecularly reminiscent of mechanisms underlying cell-state transitions in cancer.^559^ COVID-19 patients are characterized by inflammation of lung tissues, anemia, altered expression of iron-homeostasis genes, low serum iron, and inefficient oxygen transport.^560^ Activation of macrophages in settings of acute inflammation has been shown to involve CD44-mediated iron uptake and iron-dependent epigenetic programming of the inflammatory cell state.^13^ Like in cancer cells, immune cells including dendritic cells and macrophages, upregulate iron uptake to promote the activity of iron-dependent demethylases, and unlock the expression of pro-inflammatory genes.^13,446^ The role of iron in the control of cell states in immunity is also supported by a study showing that a specific histone 3 lysine 27 trimethyl (H3K27me3)-specific demethylase is involved in macrophage activation.^530^ Increased iron was shown to be associated with increased Wnt signaling in cell culture.^561^ The protein pirin has been shown to control NF-κB signaling upon binding to ferric iron.^562^ Iron(III) is susceptible to changing the conformation of pirin, enabling binding to NF-κB. The therapeutic benefits gained by interfering with these processes require further examination. DFO and Dp44 mT (Table 6) treatments also influence c-Jun N-terminal kinase (JNK) and p38 MAPK signaling pathways, leading to cell cycle arrest and apoptosis.^563^ Iron has also been shown to accumulate in senescent cells, which contribute to the fibrotic disease.^564^ In addition, nonalcoholic fatty liver disease and steatohepatitis are both characterized by iron overload in hepatic stellate cells and iron-deficient hepatocytes. Both conditions can lead to liver fibrosis.^565^ Taken together, these studies suggest that a therapy targeting iron or rebalancing iron distribution between cell types could prevent fibrosis or mitigate its effect.

##### 4.2.3.3 Iron Signaling in Neurological Disorders

Friedreich’s ataxia is a neurological disorder, where a deficiency of frataxin protein leads to aberrant iron–sulfur cluster biosynthesis, thereby impacting other cellular processes. Iron chelation using deferiprone (Table 6) has shown some clinical success, yet it remains currently unknown where in the cell this small molecule exerts its activity and mechanistic insights remain limited.^566−568^ Finally, iron imbalances have been reported extensively in neurological diseases, including in AD and PD as well as brain aging.^569−572^ However, the exact etiology of these diseases and the defects induced by iron in this context are not fully understood. Interestingly, the organoselenium small molecule ebselen can inhibit DMT1^573^ (Table 6) and has been selected for preclinical trials in the context of AD.^519,574,575^

### 4.3 Copper Signaling

#### 4.3.1 Regulation of Copper Homeostasis

**Figure 9:**
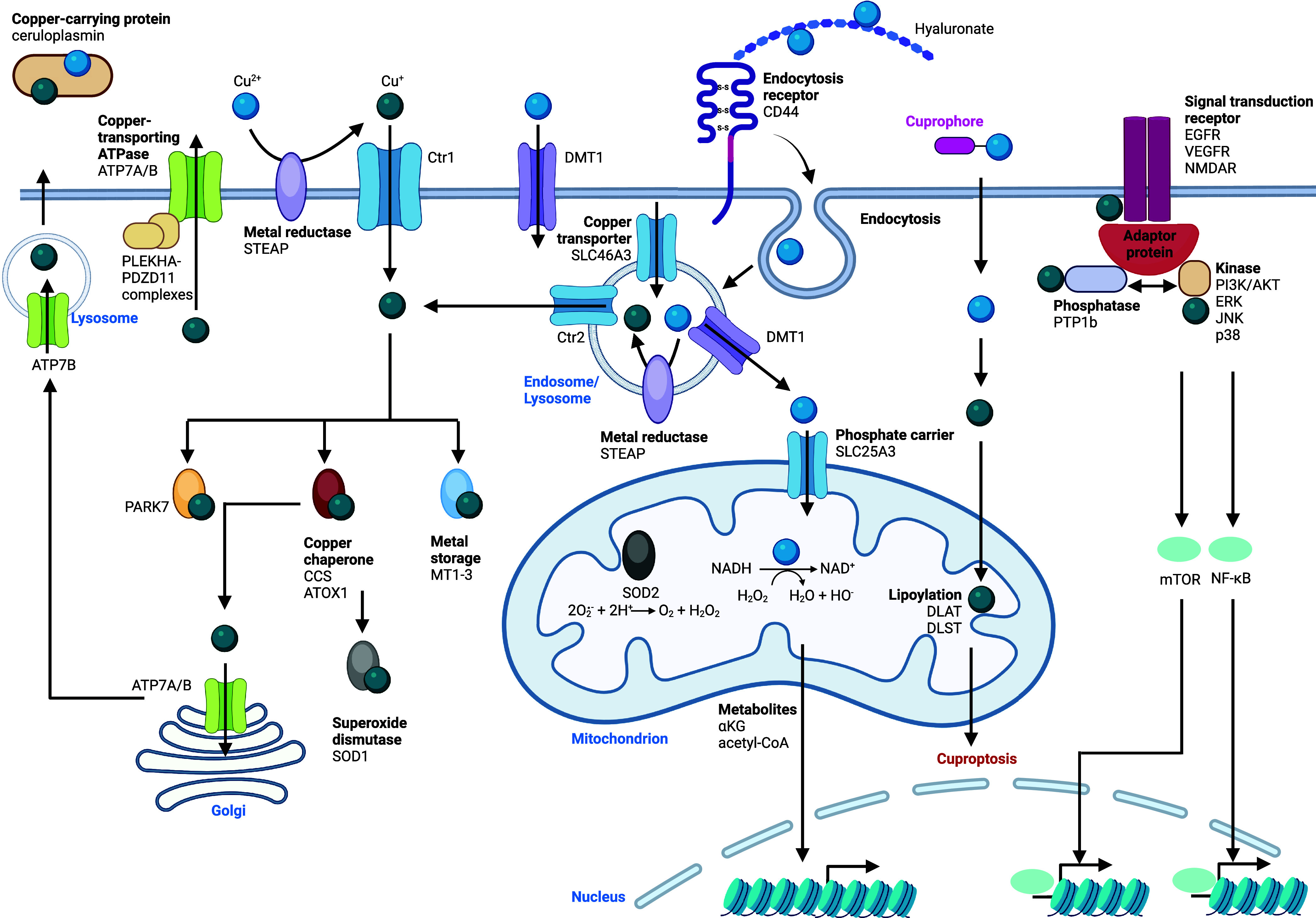
Copper signaling. Copper is taken up into the cell via Ctr1 and DMT1. Copper(II) is also internalized by means of CD44/hyaluronan-mediated endocytosis. Copper(II) is reduced into copper(I) by the metal reductases STEAP. Intracellular copper is transported to mitochondria and the Golgi apparatus. Copper can bind to chaperone proteins, such as CCS and ATOX1. CCS is responsible for the delivery of copper to SOD1, and ATOX1 to the delivery of copper to transporters ATP7A and ATP7B. Known copper storage proteins include MT1, MT2, and MT3. The cuprophore elesclomol enables the trafficking of copper to the mitochondria. Binding of copper to lipoylated components, such as DLAT and DLST, triggers cuproptosis. Mitochondrial copper allows the reduction of hydrogen peroxide by NADH to produce NAD+, which is required for the activity of several mitochondrial enzymes and the production of metabolites such as α-KG and acetyl-CoA, controlling epigenetic and transcriptional programs of inflammation and cancer metastasis. Copper can modulate signal transduction pathways by binding to growth factors, kinases, or phosphatases. Copper is exported outside of the cell by ATP7A and ATP7B. In the presence of high copper level, PLEKHA proteins bind to PDZD11, and then PLEKHA–PDZD11 complexes target ATP7A to the cell membrane. In the presence of a high copper level, ATP7B shuttles from the Golgi apparatus to lysosomes and imports copper. Ceruloplasmin is a copper-carrying protein in the blood. See Abbreviations. Figure generated with BioRender.com.

Copper is found as +1 and +2 oxidation states in the cell. Like iron, copper can participate in redox reactions under physiological conditions. In comparison with iron, cellular copper levels are about 100-fold^13^ lower in cells. In humans, heme, which is composed of an iron(II) core center, is the major component of hemoglobin, allowing for oxygen fixation and transport to cells. However, some invertebrates, including horseshoe crabs, have developed copper-based hemocyanins that play a similar role.^611^ Several mechanisms of cellular copper uptake have been documented^612^ (Figure 9). The copper transporter 1 (Ctr1)^613^ has been described as a selective copper uptake protein, which takes up copper(I) ions. This is made possible by the reduction of copper(II) by metal reductases, such as those belonging to the STEAP family at the cell surface.^456^ Additionally, in endothelial cells, DMT1 localizes at the cell surface and enables the uptake of copper(II).^614^ In macrophages acquiring a pro-inflammatory phenotype, it has been shown that increase of copper(II) results from the up-regulation of CD44, which enables endocytosis of hyaluronan-bound metals.^13^ Export of copper(II) from endolysosomes to the cytosol was proposed to involve Ctr2^615^ and potentially DMT1. Although DMT1 translocates copper(II), the copper species that is transported by Ctr2 has not been well documented. STEAP enzymes are present in lysosomes, and it is conceivable that STEAP4 mediates the reduction of copper as it is emerging as an important regulator for copper homeostasis in the cell.^616,617^ In addition, SLC46A3 was identified as a lysosomal copper transporter.^618^ Copper transport and export mechanisms involve copper-transporting ATPase 1/2 (ATP7A/B). These ATPases tightly control cytosolic copper levels,^619,620^ which is supported by structural data.^621^ Cellular export of copper by ATP7B has been described to occur by exocytosis.^622^ In addition, these ATPases can transport copper to and from the Golgi apparatus. Other proteins have been implicated in the regulation of the ATP7A/B localization in the cell. For instance, pleckstrin homology domain containing family A 5 (PLEKHA5), PLEKHA6, and PLEKHA7 recruit PDZ domain-containing protein 11 (PDZD11) to distinct parts of the plasma membrane to promote the anterograde transport of copper by ATP7A to the cell periphery.^623^ Furthermore, SLC25A3 is a phosphate transporter found in the inner mitochondrial membrane that transports copper via the mitochondrial membrane.^624^ Copper can be sequestered and stored in the cell by metallothioneins (MTs), such as MT1, MT2, and MT3.^625^ MTs can also coordinate other metals, such as zinc, with some metal preferences. For example, MT3 has been shown to exhibit the highest affinity for copper within this class of proteins.^626^ Thus, balancing the expression of different MTs provides cells with the ability to control the levels of copper and zinc in the cytosol. Interestingly, copper(II) can catalyze the oxidation of GSH to glutathione disulfide (GSSG).^627^ The rate of copper-catalyzed oxidation of GSH to GSSG has been reported to be much lower than that of copper(II) complex formation with GSH, meaning that complexes between copper(II) and GSH can potentially exist in the cell before oxidation to GSSG occurs. In addition, GSH can form complexes with copper(I) to form tetranuclear [Cu_4_(GS)_6_] complexes, limiting free aquacopper(I) in the cell.^628^ Taken together, GSH can limit the concentrations of free copper(I) and copper(II) in the cell. Importantly, copper(I)/copper(II) redox cycling and ROS formation are influenced mainly by GSH and cysteine levels in the presence of hydrogen peroxide, suggesting that GSH and cysteine are important for buffering copper toxicity in the cell.^629^ Copper ions may also act as a catalyst for other chemical reactions in the cell in the presence of GSH, notably in mitochondria.^630^ Since copper(II) can participate and promote redox reactions in the cell, specific chaperones have evolved to regulate the pool of available chemically reactive copper. For example, specific chaperones including antioxidant 1 copper chaperone (ATOX1), also called Hah1,^631^ regulate copper(I) distribution in the cell, deliver the metal to copper-dependent enzymes and transfer it to ATP7A/B.^632−634^ The copper chaperone for superoxide dismutase (CCS)^635^ transfers the metal to SOD1, which unlike SOD2 predominantly localizes in the cytosol and contains a copper–zinc binuclear catalytic site responsible for the disproportionation of superoxide radicals. However, the role of zinc was not found to be critical for the enzymatic activity of superoxide dismutase under acidic conditions.^636^

#### 4.3.2 Cellular Functions of Copper

In mitochondria, copper is essential for several processes. It is involved in cuproproteins, enabling electrons to shuttle through the ETC. It plays an integral role in cytochrome *c* oxidase (also termed complex IV or COX), where three copper ions are coordinated inside the protein complex.^637,638^ This complex, which resides in the inner mitochondrial membrane, is the last enzyme of the ETC,^639^ which produces an electrochemical proton gradient that drives the synthesis of ATP. Mechanistically, copper is delivered to cytochrome *c* oxidase assembly protein (Sco1) by the copper chaperone COX17.^640,641^ Then, the copper chaperones Sco1 and Sco2 deliver copper to cytochrome *c* oxidase.^642,643^ Copper is also required for the activity of the Parkinson disease protein 7 (PARK7), which detects oxidative stress and resides in mitochondria, the nucleus, the ER, and the cytosol.^644^

In mitochondria, copper(II) has been detected in inflammatory macrophages and found to act as a Lewis acid, activating hydrogen peroxide and catalyzing the oxidation of NADH into NAD+ (see Figure 2IIIc). This chemical reaction has been shown to drive metabolic and epigenetic programming of inflammation in macrophages.^13^ Interestingly, activation of other immune cells including dendritic cells, and acquisition of a pro-metastatic DTP cancer cell state, have been shown to be driven by a similar copper-dependent chain of event.^13^ Whether this reaction is assisted by a protein or solely relies on free copper(II) remains unknown. Given that imidazole further increases the rate of copper-catalyzed NAD(H) redox cycling, it is conceivable that this reaction is assisted by a mitochondrial protein. In addition, copper is involved in several cell signaling pathways, acting as a component of several proteins, including kinases, phosphatases, and adapter proteins,^645^ impacting JNK,^646^ NF-κB,^646−648^ mTOR, and MAPK signaling pathways.^649,650^ In these cases, copper acts by means of metalloallostery promoting functional enzyme folding.^651^ For instance, copper can activate MAPK signaling, whereas disruption of the cellular copper import was found to reduce kinase activity. The MAPK kinase MEK1 binds two copper ions with high affinity, enhancing its catalytic activity.^652^ The copper chaperone CCS facilitates transfer of copper to MEK1 and MEK2, enhancing kinase activity.^653^ In addition, the copper chaperone ATOX1 is required in MAPK signaling in melanoma cells.^654^ Another study showed that casein kinase 2 (CK2) also requires copper for its catalytic activity.^655^ UNC51-like kinase-1 (ULK1) and ULK2 act downstream of mTOR and are activated by copper, with implications in adenocarcinoma.^656^ Copper can also directly activate PI3K/AKT signaling,^657^ however, explicit mechanisms at work remain incompletely understood. These studies support a functional role for copper in signaling pathways activated in cancer and inflammation. Designing selective inhibitors of these proteins or hijacking copper represents an appealing therapeutic angle. Copper overload in the cell has been linked to increased aggregate formation between lipoylated proteins via disulfide bond formation catalyzed by copper, predominantly occurring in mitochondria. Elesclomol (Table 7) has been shown to act as a cuprophore increasing intracellular copper levels, promoting this phenotype and leading to a form of cell death called cuproptosis.^658^ Finally, copper has also been reported to play a role in lipolysis by inhibiting the activity of the cyclic adenosine monophosphate (cAMP)-degrading phosphodiesterase 3B (PDE3B) via direct binding to the top cysteine residues of the enzyme in a mouse model of dysregulated copper homeostasis.^659^ Manipulating copper homeostasis may therefore be exploited for therapeutic intervention.

#### 4.3.3 Copper Signaling and Diseases

Copper overload diseases with genetic background have been documented.^660^ MD^661^ is an X-chromosome linked recessive disorder with mutations in ATP7A. WD^662^ is an autosomal recessive disorder linked to mutations in ATP7B.^663−666^ MD is characterized by copper deficiency in specific tissues, leading to growth and developmental defects, whereas copper buildup is found in WD primarily in brain and liver, impacting behavior and causing digestive disorders. These diseases highlight the central role of cellular copper homeostasis in human physiology. MD is clinically managed by copper supplementation, including subcutaneous copper injections,^667^ elesclomol and L-DOPS (Droxidopa)^668^ (Table 7) treatment has been explored to rebalance copper distribution. In addition, metal chelators can be used to alter copper availability,^669^ and WD is treated using copper chelators, such as d-penicillamine, tetrathiomolybdate (TTM), trientine,^649^ and ATN-224,^670^ among others^647,649^ (Table 7).

**Table 7:**
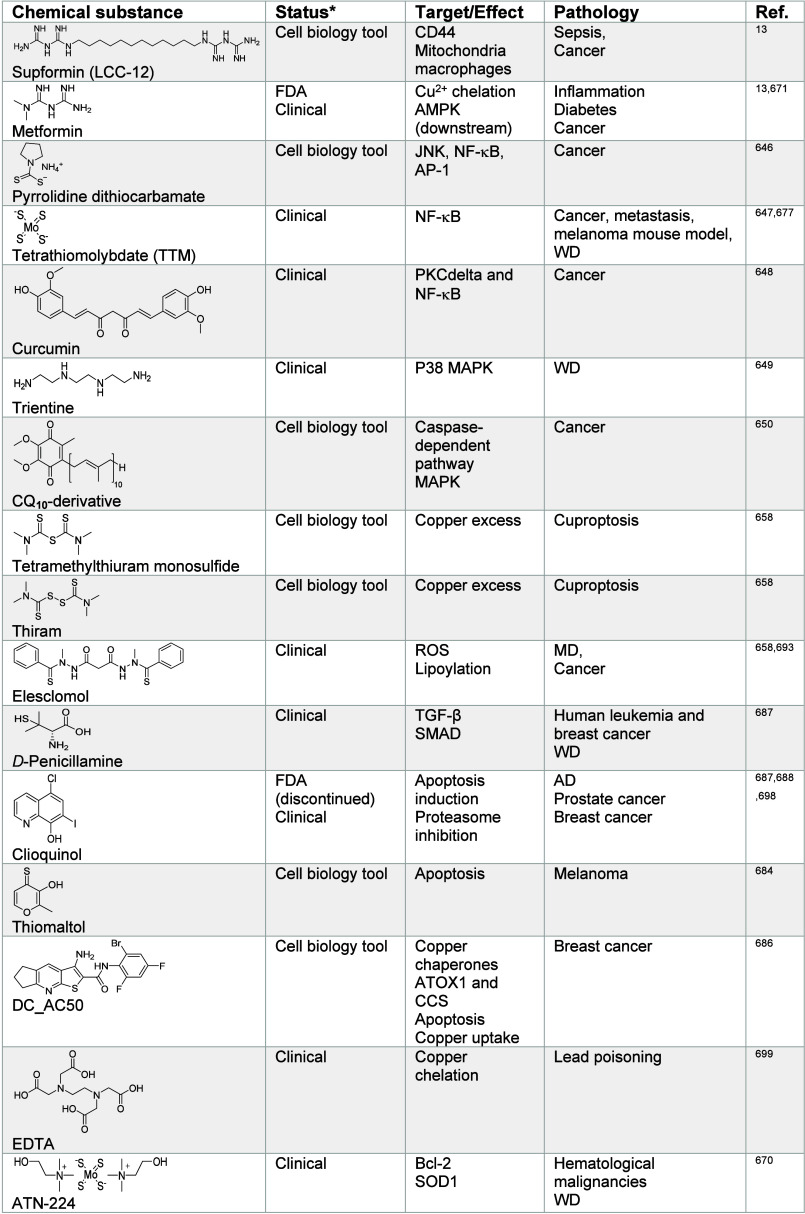

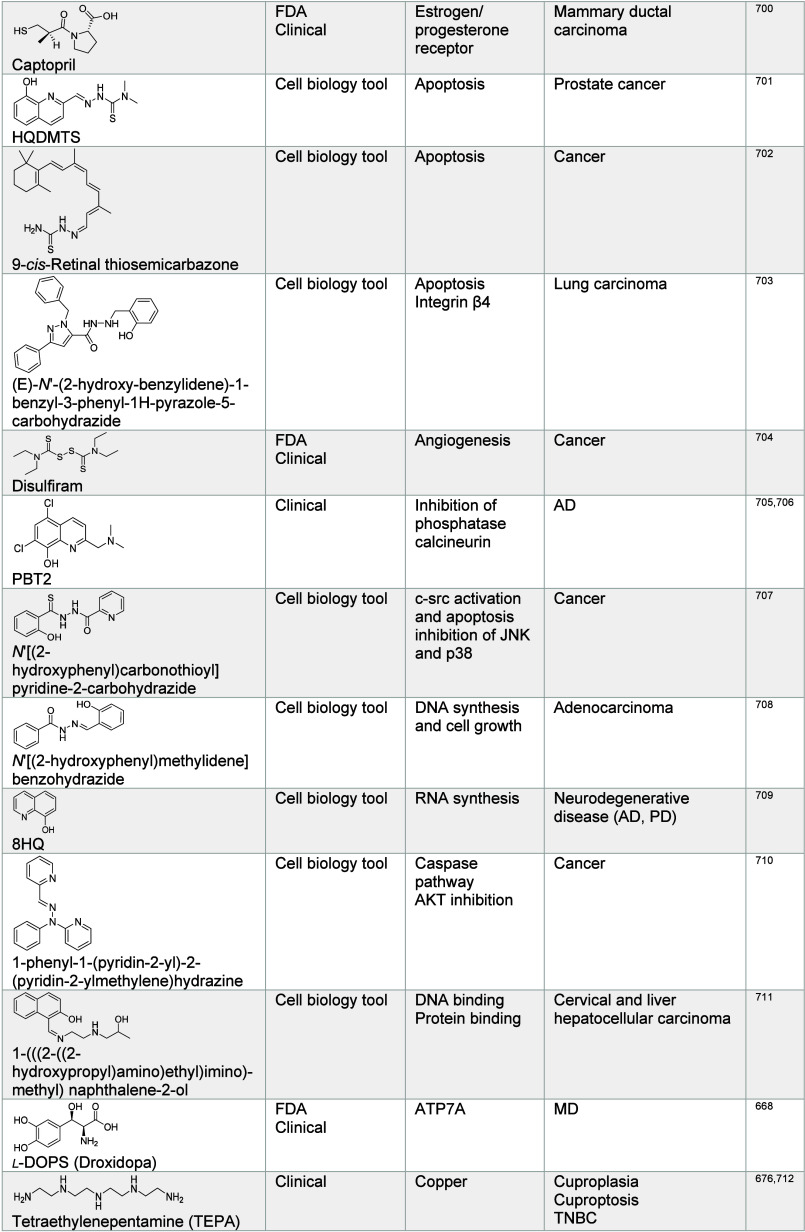

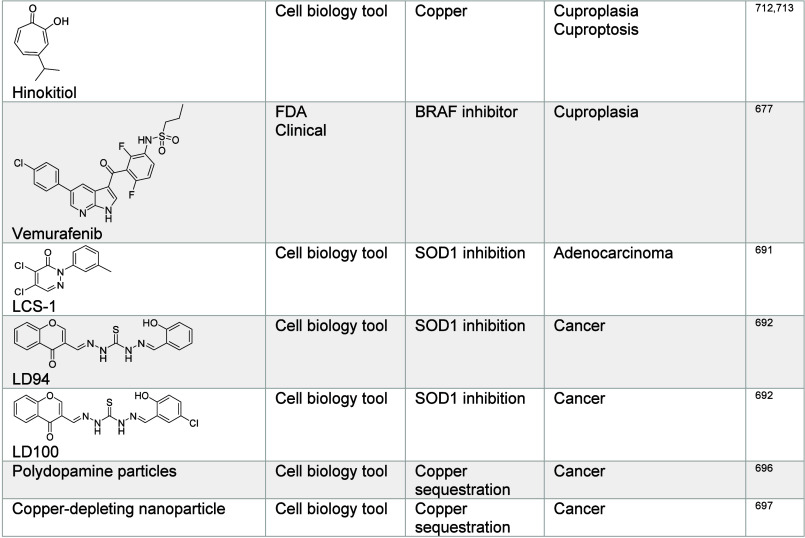
Regulators of Copper Signaling^13,592,646−650,658,668,670,671,676,677,684,686−688,691,693,696−702,703,704,705−713,793,794^

* https://www.fda.gov, https://clinicaltrials.gov

##### 4.3.3.1 Copper Signaling in Immunity and Inflammation

Copper levels have been reported to be increased in inflamed malignant tissues and a STEAP4-IL17-dependent signaling axis has been documented in this context.^616^ Pro-inflammatory macrophages and cancer cells acquiring a pro-metastatic drug tolerant mesenchymal state are characterized by upregulation of CD44, increase of copper uptake, and a higher load of mitochondrial copper(II). Selective inactivation of mitochondrial copper(II) with small molecules such as metformin, identified by phenotypic screen, and the rationally designed dimer of biguanides supformin (LCC-12) (Table 7), were found to interfere with NAD(H) redox cycling and to antagonize specific epigenetic and transcriptional programs, notably those underlying inflammation.^13,487^ Interestingly, the basic pH of the mitochondrial matrix (i.e., proton gradient), but not mitochondrial copper(II), was found to drive the accumulation of biguanides in mitochondria, indicating that it is the organelle physiology that defines the subcellular localization of these biologically active compounds as opposed to the mechanistic target (i.e., copper(II)), emphasizing the value of phenotypic screen and target identification studies. Remarkably, copper(I) chelators and the copper chelator trientine, which accumulates in the nucleus, were found to be biologically inactive against pro-inflammatory macrophages. Pharmacological inactivation of mitochondrial copper(II) blocked immune cell activation, reducing acute inflammation in disease-relevant settings, and interfered with the acquisition of a DTP cancer cell state.^13,671^ Targeting mitochondrial copper(II) represents a powerful strategy to control the fate of immune cells for the clinical management of acute inflammation. It is not yet clear whether chronic forms of inflammation and autoimmune diseases are reliant on similar mechanisms and whether such a therapy can be exploited in these contexts. In COVID-19 patients, cytokine release syndrome characterizes the most severe cases.^672^ Remarkably, patients with diabetes undergoing metformin (Table 7) treatment exhibited less severe symptoms of COVID-19.^673^ Furthermore, COVID-19 patients were found to upregulate hyaluronan biosynthesis in the lung,^448,449^ supporting the contention that CD44/hyaluronan-mediated copper uptake drives immune cell activation in the context of acute inflammation. Thus, mitochondrial copper(II) inactivation with small molecules, such as supformin (Table 7) holds great promise for the clinical management of acute inflammation and sepsis.

##### 4.3.3.2 Copper Signaling in Cancer

Copper has been implicated in cancer progression^674^ and copper chelators are being developed to specifically target cancer cells.^675^ Although such approaches are appealing, additional mechanistic rationales are needed to explain why specific copper chelators can target a cell niche in cancer. Mitochondrial copper(II) has been shown to regulate EMT and acquisition of a DTP cancer cell state.^13^ It is not clear whether controlling cancer cell identity through mitochondrial copper(II) inactivation can prevent metastasis in vivo or resensitize cancer cells to standard-of-care antiproliferative drugs. Nevertheless, compounds such as supformin (Table 7) represent valuable tools to perturb and dissect the biology of cancer cells that may be exploited for therapeutic purposes. An interesting study showed that the copper(II) chelator tetraethylenepentamine (TEPA) (Table 7) reduced the EMT signature in triple-negative breast cancers in mice and lead to a reduction of lung metastasis in this model.^676^ Whether TEPA chelates copper outside or inside of the cell and its subcellular distribution remain to be elucidated. The role of copper in MAPK signaling has direct implications in cancer, particularly in melanoma and thyroid cancers, where V600E mutations of v-raf murine sarcoma viral oncogene homologue B1 (BRAF) have been observed. BRAF(V600E) phosphorylates MEK1/2, which can then phosphorylate the ERK1/2 kinases, thereby activating MAPK signaling. The copper chelator tetrathiomolybdate (TTM) (Table 7), which is used for the treatment of WD, has been shown to reduce melanoma growth in mouse models.^677,678^ BRAF can be directly targeted and inhibited by vemurafenib (Table 7), and the cytostatic activities of copper chelators have been shown to contribute to their antineoplastic properties.^677^ Interestingly, pharmacological inhibition of Bcl-2 showed synergistic effects with TTM in BRAF(V600E) melanoma, inducing apoptosis.^679^ It will be important to dissect which copper-dependent pathway is impaired in these settings. In another study, TTM administration was found to reduce ceruloplasmin levels and the number of VEGFR2^+^ endothelial progenitor cells in the tumor microenvironment in triple-negative breast cancer patients with favorable survival enhancement.^680^ In addition, the same study showed reduced lung metastasis in preclinical mouse models of triple-negative breast cancer. Taken together, this work illustrates the value of copper chelation therapy and how it can be used in combination with other anticancer drugs for therapeutic benefits. Disulfiram (Table 7) has been reported to effectively kill human glioblastoma cell lines.^681^ In combination with copper, disulfiram has been shown to be effective against CSCs. It caused increased ROS production, subsequent inhibition of aldehyde dehydrogenase (ALDH) and NF-κB signaling.^681^ Interestingly, docetaxel-resistant prostate cancer cells were shown to express increased levels of ATP7B. Interestingly, these cells were resensitized to docetaxel upon treatment with disulfiram and copper.^682^ These promising findings warrant further examination.^683^ The ionophore thiomaltol (Table 7) has also been shown to exhibit promising cytotoxic effects in melanoma cells in combination with copper supplementation.^684^ The small molecule pyrrolidine dithiocarbamate^646^ (Table 7) is a copper chelator that was shown to affect NF-κB, JNK, and activating protein-1 (AP-1) signaling in various cancer cell lines, providing the basis for new therapeutic strategies. In addition, the copper chaperone ATOX1 has been identified as a potential prognosis marker in estrogen receptor-positive subtypes and also for early stage breast cancers.^685^ Additional work is needed to understand the functional role of copper homeostasis in this context to enable the development of copper-based therapeutic strategies. To this end inhibitors of copper-trafficking proteins such as 3-amino-*N*-(2-bromo-4,6-difluorophenyl)-6,7-dihydro-5*H*-cyclopenta[*b*]thieno[3,2-*e*]pyridine-2-carboxamide (DC_AC50) and its derivatives, which inhibit ATOX1 and CCS, were found to inhibit cancer cell proliferation^686^ (Table 7). The ionophore clioquinol^687,688^ (Table 7) has been shown to exhibit promising activity against prostate cancer in mice.^689^ However, this compound can form complexes with zinc and copper, making it challenging to assign an explicit MoA in this context.^690^ A screen for small molecule inhibitors of adenocarcinoma cell proliferation identified SOD1 as a promising target with the discovery of the SOD1 inhibitor lung cancer screen 1 (LCS-1)^691^ (Table 7). Furthermore, specific inhibitors against SOD1, such as LD94^692^ and LD100^692^ (Table 7), have also been reported to directly bind to copper in the active site of the enzyme.^692^ In addition, elesclomol has been selected to undergo clinical trials for the treatment of melanoma and may hold potential as a new anticancer therapeutic.^658,693^ Although elesclomol has been reported to induce cuproptosis, causing aggregation of lipoylated proteins via disulfide bond formation, it is not clear how elesclomol exerts its activity under these conditions.^658^ Investigating copper levels and oxidation states in specific cell organelles, as well as quantifying GSH levels is warranted to illuminate how manipulating copper homeostasis could be exploited for therapeutic benefits.^694,695^ Recent work has shown that copper-depleting nanoparticles can sequester copper, reducing tumor growth in mice.^696,697^ Such nanoparticles thus provide an interesting starting point to develop anticancer therapies based on copper depletion.

### 4.4 Zinc Signaling

#### 4.4.1 Regulation of Zinc Homeostasis

**Figure 10:**
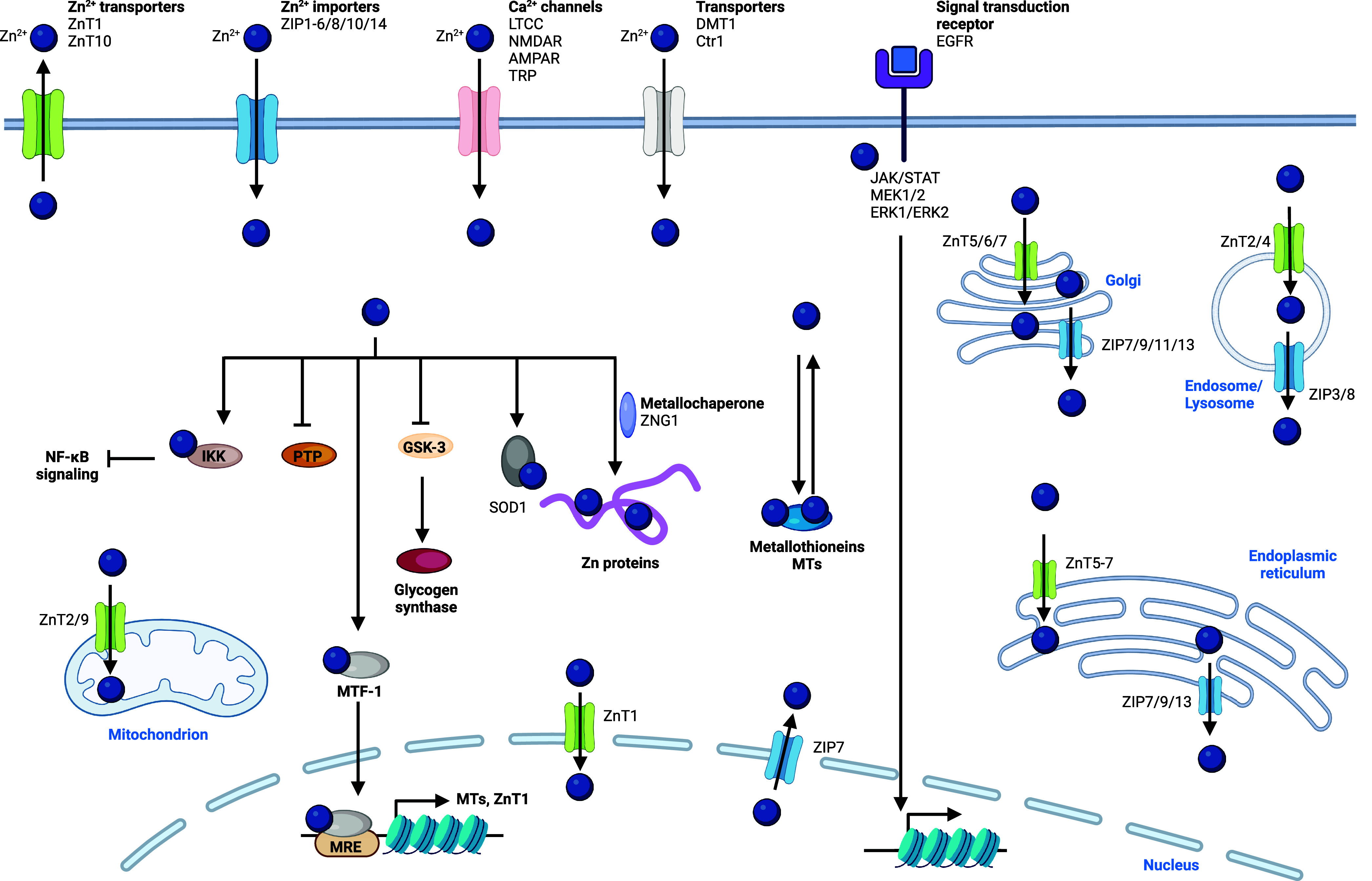
Zinc signaling. Zinc is taken up into the cell via ion importers/transporters and calcium channels. Zinc is trafficking to various organelles including the Golgi apparatus, the ER, endolysosomes, mitochondria, and the cell nucleus. Metallothioneins bind to zinc and regulate the ion distribution within the cell. Zinc can activate MTF-1 in the cytosol; MTF-1 translocates to the nucleus, where it binds to MRE on the promoter regions of zinc-dependent genes, inducing the transcription of MTs. In the cytosol, zinc inhibits the kinase GSK-3 by promoting phosphorylation, thereby enhancing the glycogen synthase activity. Zinc inhibits protein tyrosine phosphatases (PTP), and the activation of IKK, which antagonizes NF-κB signaling. The metallochaperone ZNG1 delivers zinc to specific metalloproteins. Zinc is exported outside the cell by specific transporters, such as ZnT1 and ZnT10. See Abbreviations. Figure generated with BioRender.com.

Zinc is found as a +2 oxidation state in the cell, where it is generally considered a redox inactive metal. Yet, interactions with cysteines can confer a redox activity to zinc ions in the cell.^714,715^ Zinc is taken up into the cell by specific importers such as ZIP1–6, ZIP8, ZIP10, ZIP14, and TRPM7^716−718^ (Figure 10). Other ion channels that have been characterized for calcium import as well as DMT1 and Ctr1 have also been reported to regulate the uptake of zinc ions.^719,720^ This illustrates the lack of selectivity of these channels and transporters for a given metal. Several organelle-specific zinc transporters have been documented for the import of zinc into mitochondria, the Golgi apparatus, the ER, the endolysosomal compartment and the nucleus, all belonging to the zinc transporter (ZnT) family of proteins^717,718^ (Figure 10). Importantly, ZnT1 and ZnT10 have been described to reside in the cell membrane, where they can mediate the export of zinc from the cell. Interestingly, ZnT1 has been described as a zinc/calcium antiporter, supporting the notion that zinc can directly contribute to the control of calcium levels in the cell.^721^ Intracellular zinc levels can also be controlled by storage proteins including MTs,^625,722,723^ from which the metal can be released in a manner that is dependent on the reducing environment of the cell.^715^ It has also been reported that zinc ions can bind to and induce a conformational change of ZIP4, leading to endocytosis of the complex.^724^ Transporters for zinc into and from various cell organelles have been identified, including ZnT2/4 for zinc import into the endolysosomal compartment and ZIP3/8 for zinc translocation from these organelles (Figure 10). ZnT5/6/7 transport zinc into the Golgi apparatus and ZIP7/9/11/13 were reported to export zinc from the Golgi apparatus. For the ER, ZnT5/6/7 have been characterized as zinc importers and ZIP7/9/13 as exporters. ZnT2/9 have been shown to mediate zinc uptake into mitochondria, whereas ZnT1 can import zinc into the nucleus and ZIP7 translocates the metal from the nucleus to the cytosol. Zinc is a key component of metal response element transcription factor-1 (MTF-1), which controls the transcription of ZnT1 and MTs, forming a positive feedback loop that regulates zinc levels in cells.^725^ Little is known about cellular zinc chaperones. A family of COG0523 zinc chaperone proteins conserved across vertebrates, which was named zinc-regulated GTPase metalloprotein activator (ZNG1) family, has been reported.^726^ ZNG1 proteins transfer zinc to methionine aminopeptidase 1 (METAP1) and ZNG1 knockdown resulted in the impairment of cellular zinc homeostasis and mitochondrial dysfunction.

#### 4.4.2 Cellular Functions of Zinc

The general high affinity for zinc binding to its biological substrates confers this metal cell signaling capacity by competing out other metals from biomolecules. For example, zinc has been shown to influence calcium homeostasis.^727,728^ Zinc is involved in cell proliferation and growth, being an important cofactor of TFs, potentially also acting through hormonal regulation of cell proliferation.^729^ Zinc is an important cofactor of zinc-finger proteins (ZNFs), constituting a large group of proteins.^730^ Roles of these proteins are widespread, including transcriptional regulation via ZNFs that are TFs, DNA repair, and signal transduction among others. Zinc is thus a crucial component of proteins that interact with nucleic acids. Zinc is important for the activity of many TFs, notably those involved in TGF-β and JAK/STAT signaling.^730−732^ The importance of ZIP6 in STAT signaling, which involves zinc, has been documented.^733,734^ Zinc and vanadium can also activate EGFR signaling and the Ras-dependent activation of MEK1/2 and ERK1/2 signaling by affecting the phosphorylation status of several components of the signaling cascade,^735^ although the exact mechanism orchestrating this is yet to be elucidated. Evidence for a role of zinc signaling in disease has been reported in cancer and inflammation.^736^ Interestingly, zinc has been shown to directly inhibit IκB kinase β (IKKβ) activity upon binding to a specific site located in the kinase domain of the protein,^737^ leading to a downregulation of NF-κB signaling. Similar effects of zinc were observed for protein kinase Cδ,^738^ indicating that zinc controls the activity of various protein kinases in the cell, affecting a multitude of pathways. Zinc has also been shown to impact stem cell pluripotency, shaping cell fate.^739^ A study showed that zinc is required for human embryonal development during egg activation, where a so-called zinc spark has been observed.^740^ Interestingly, intracellular zinc chelation led to a switch from a meiotic cell into a mitotic cell, suggesting that zinc is required for epigenetic control. Importantly, zinc is found in the active site of HDACs.^741,742^ Histone acetylation plays an important role in the epigenetic control of gene expression and can determine cell fate decisions. Taken together, these results indicate that zinc is involved in the control of cell identity. Zinc is also a component of SOD1, which is mainly found in the cytosol, and thus may play a role as a regulator of radical superoxide levels and hydrogen peroxide levels in the cell, potentially impacting oxidative stress and other ROS-dependent cell signaling pathways.

#### 4.4.3 Zinc Signaling and Diseases

##### 4.4.3.1 Zinc Signaling in Cancer

Zinc is widely implicated in transcriptional activation via ZNFs^743^ and in various signaling pathways in cancer.^744^ For example, the zinc finger protein p66β is a coactivator of the zinc finger transcription factor Snail, which plays crucial roles in the regulation of cell plasticity during development and in cancer.^745^ Cancer cell plasticity can give rise to a cell state refractory to standard-of-care treatments, constituting a drug-tolerant persister state. Interestingly, a study showed that KS10076 (Table 8), which can chelate copper, zinc, iron, and manganese, can eradicate ALDH1^+^ CSC subpopulations, characterized by a drug-tolerant cell state.^746^ The lack of metal specificity, however, limits mechanistic insights and raises the question of whether a specific role of zinc dominates this phenotype or whether altering homeostasis of other metals impairs specific cancer cell subpopulations. The putative role of zinc in cancer has prompted interest for the targeting of ZIP and ZnT family of transporters.^717^ ZIP7 plays a role in zinc transport from the ER, the Golgi apparatus, and the nucleus to the cytosol. Interestingly, protein kinase CK2 can trigger cytosolic zinc signaling by phosphorylation of ZIP7. Since tyrosine kinase signaling is dysregulated in various cancers, targeting ZIP7 selectively in cancer may confer therapeutic benefits.^747^ Small molecules such as 2-dimethylamino-4,5,6,7-tetrabromo-1*H*-benzimidazole (DMAT) and 4,5,6,7-tetrabromobenzotriazole (TBB)^748^ (Table 8) have been shown to reduce the proliferation of breast cancer cells, whereas the ZIP7 inhibitor NVS-ZP7-4 was found to be effective against T cell acute lymphoblastic leukemia.^749^ In addition, CK2 inhibitors like CX-4945^748,750^ (Table 8) can interfere with ZIP7 phosphorylation and have been developed for the treatment of estrogen receptor-positive breast cancer.^748,750^ Other anticancer strategies include targeting ZIP5,^751^ targeting ZIP6 with antibody conjugates,^752−754^ as well as other small molecules interfering with signaling pathways implicating ZIP9 and ZIP10 (Table 8). For instance, dutasteride^755^ (Table 8) has been developed for the treatment of bladder cancer, whereas bicalutamide^756^ is used for the treatment of melanoma and GSK690693^757^ (Table 8) for the treatment of osteosarcoma. The zinc chelator *N*,*N*,*N*′,*N*′-tetrakis(2-pyridinylmethyl)-1,2-ethanediamine (TPEN) (Table 8) has been shown to reduce the cell proliferation of breast and pancreatic cancer cells, suggesting that zinc is required for the maintenance of a highly proliferating cell state. Although not a suitable drug candidate, this study further validates zinc chelation as a possible anticancer therapy to target highly proliferating cells.^758−761^ Zinc chelation has also been investigated for therapeutic applications in other cancers, including small molecules (Table 8) such as clioquinol^762−764^ for prostate cancer, diethylenetriaminepentaacetic acid (DTPA) for breast cancer,^758^ and disulfiram for melanoma and liver cancer.^765,766^ Furthermore, zinc is required for the activity of several DNA repair/checkpoint proteins, for instance, apyrimidinic endonuclease, aprataxin PNK-like factor, and checkpoint protein with FHA and RING domains,^767,768^ protecting cells against DNA damage. Thus, imbalances in zinc homeostasis can impair these protective mechanisms and lead to genome instability with a potential impact on tumorigenesis.^767^ Since zinc is a crucial catalytic component of HDACs, changes in zinc homeostasis can potentially impact histone acetylation. Many HDAC inhibitors targeting the metal in the active site have been developed for therapeutic use, including vorinostat for cutaneous T cell lymphoma, panobinostat for multiple myeloma, and belinostat (Table 8) for the treatment of peripheral T cell lymphoma.^769−772^ However, HDAC inhibitors have not been used successfully against solid tumors. It has been reported that HDAC inhibitors can promote metastasis of solid tumors such as breast cancer.^773,774^ This can be explained by the fact that cell state transitions underlying the metastatic phenotypes require alterations of histone acetylation.^11,445,775^ Thus, HDAC inhibitors may adversely perturb epigenetic programs, regulating cancer cell proliferation and metastasis.

**Table 8:**
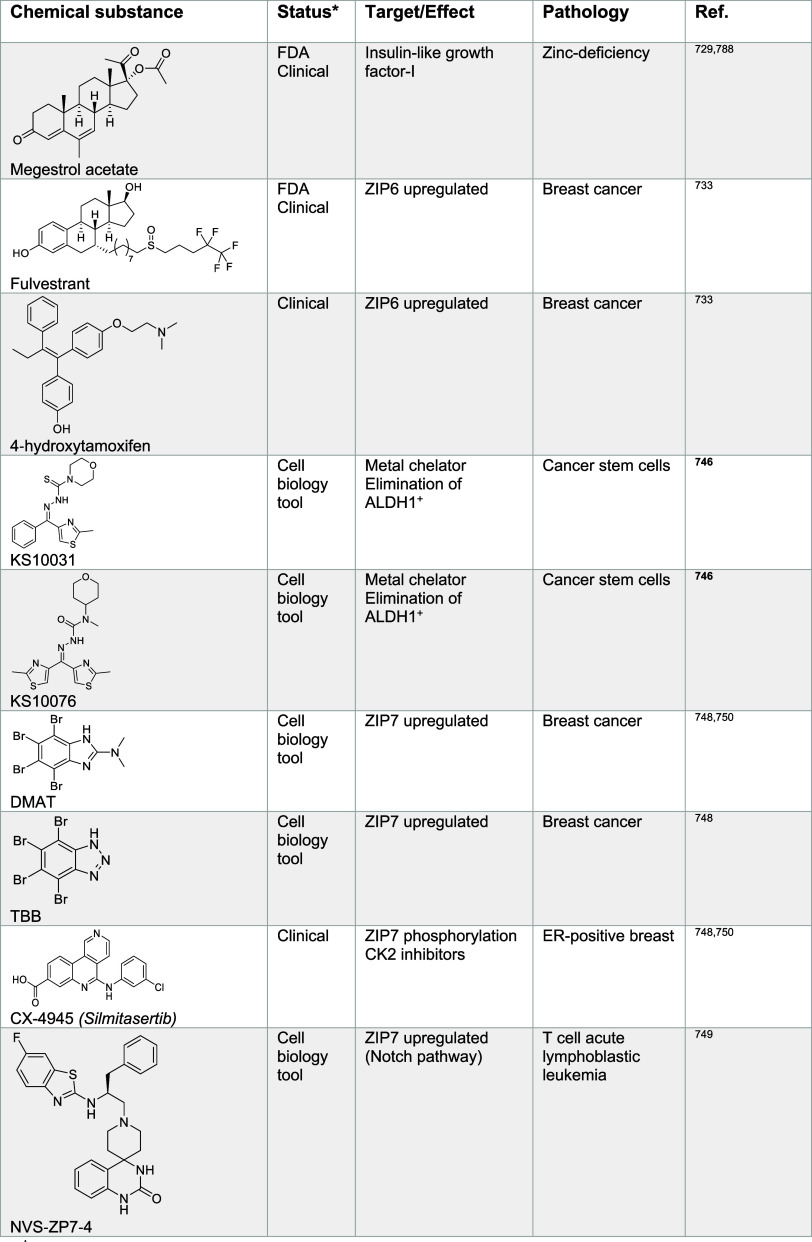

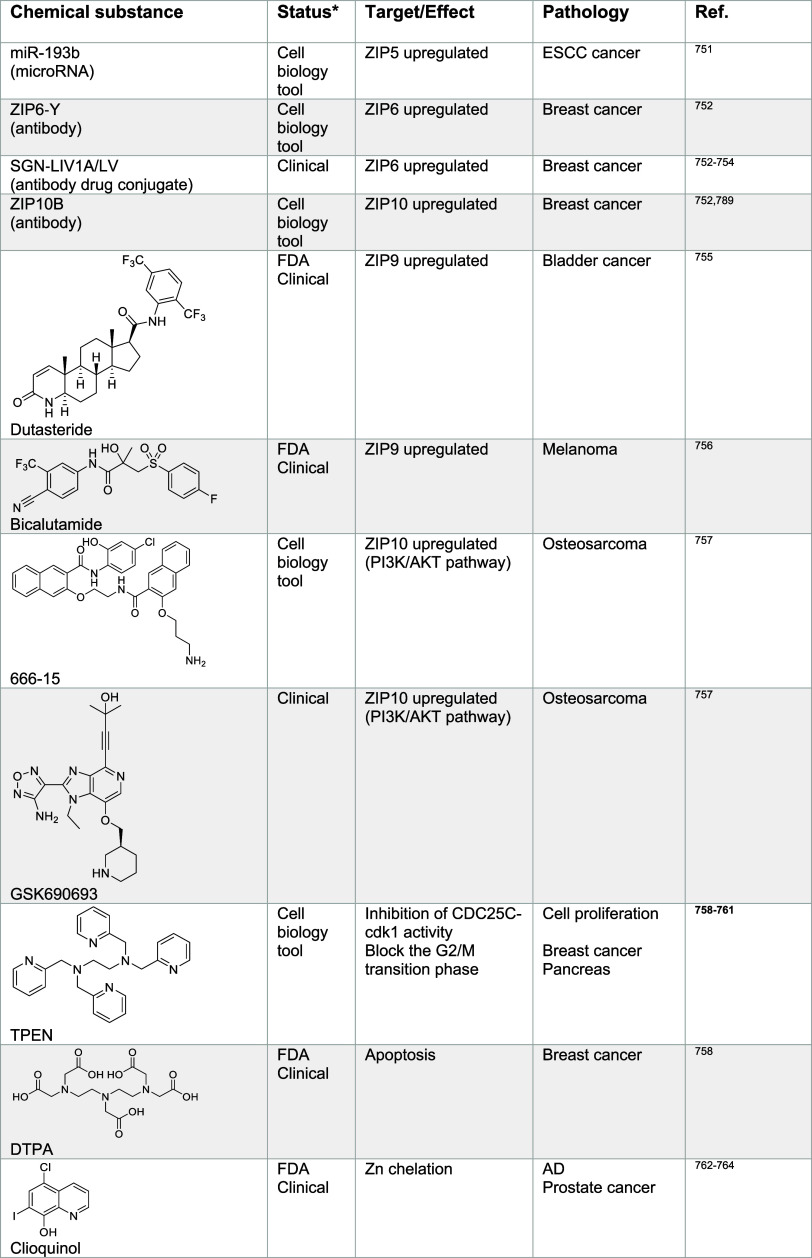

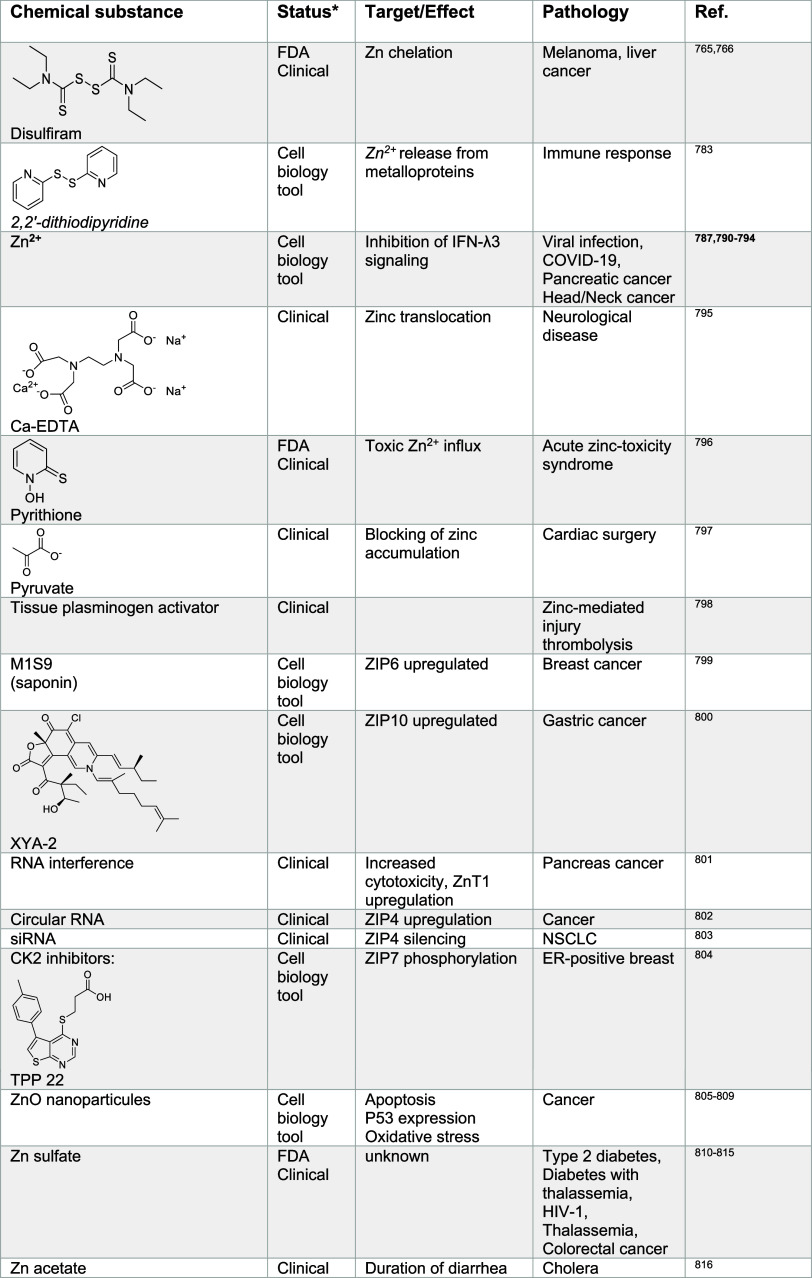

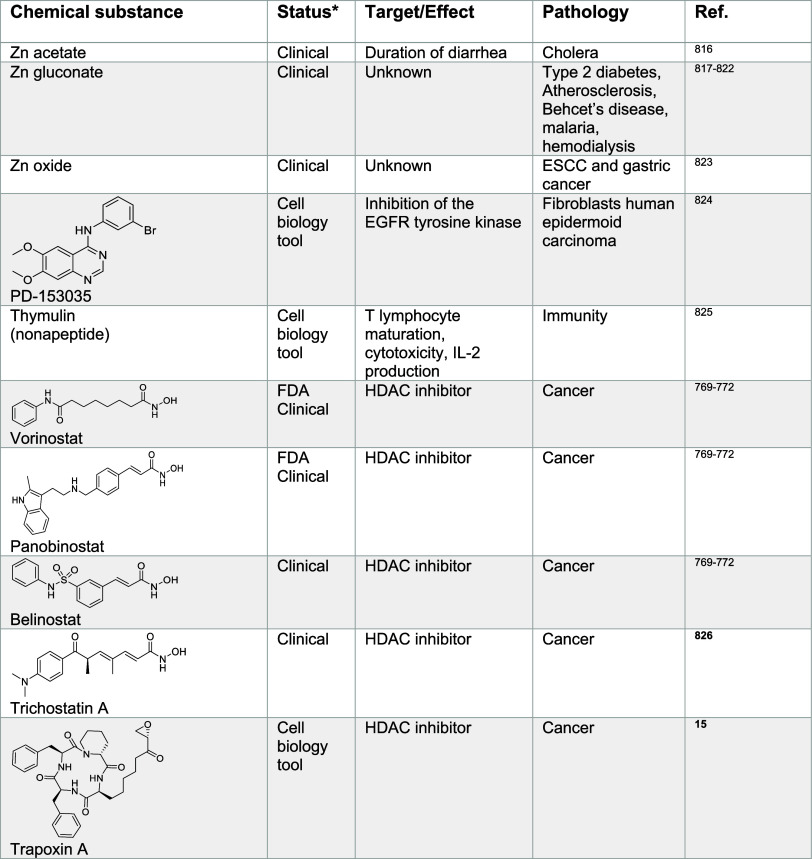
Regulators of Zinc Signaling^15,729,733,746,748−766,769−772,783,787−826^

* https://www.fda.gov, https://clinicaltrials.gov

##### 4.4.3.2 Zinc Signaling in Immunity and Inflammation

Zinc plays a crucial role in immunity.^776^ It is involved in ZNFs, which are important for immune cell function, and this includes several TFs.^777−780^ Furthermore, zinc transporters have been shown to be involved in innate immunity.^719^ Zinc generally represses NF-κB signaling, which plays important roles in immune cell activation.^781^ The inhibitory effect of zinc on NF-κB can potentially impact the activation of several types of immune cells. Thus, zinc supplementation or chelation could be exploited in specific pathological settings to modulate immune responses.^737^ Importantly, zinc signaling has been shown to be crucial for T and B cell activation.^782^ Interestingly, it has been shown that zinc can be supplied not only from extracellular sources, but also intracellular zinc is released from storage sites in immune cells during immune cell activation. For instance, MTs can stimulate zinc(II) release and activate CD4+ differentiation, which suggests that intracellular zinc reservoirs can be exploited to regulate cell state transitions.^783^ Interestingly, the small molecule 2,2′-dithiodipyridine (Table 8) can induce the release of zinc from MT, leading to increased CD4+ activation.^783^ Indeed, regulation of zinc homeostasis by MTs has been proposed to play a critical role in immune cell plasticity.^722,784^ Furthermore, free intracellular zinc has been described as a key determinant of immune cell plasticity in virtually all types of immune cells.^785^ Interestingly, upon T cell stimulation by cytokines, increases of zinc and calcium levels have been reported.^786^ Further investigation is needed to delineate the precise effect of zinc on cell signaling and cell plasticity in this context to be able to establish selective small molecule regulators of these processes. Zinc has been shown to exacerbate inflammation in viral liver disease. In this context, zinc has been shown to interfere with cytokine IFN-λ3 binding to interferon lambda receptor 1 (IFNLR1), which decreased antiviral activity and leads to increased viral replication in cells infected with influenza or hepatitis C virus.^787^ Thus, zinc chelation represents an interesting strategy to target chronic diseases linked to viral infection.

### 4.5 Cell Signaling of Other d-Block Metals (Cr, V, Mo, Co, Ni)

Other d-block metals including chromium, vanadium, cobalt, nickel, and molybdenum have been reported to play a role in the biology of the cell. In comparison to other metals, little is known about cellular homeostasis, roles in cell signaling, and implications in diseases. In this section, we document the biology of these metals and how this knowledge may be exploited for basic research and medical applications.

#### 4.5.1 Regulation of Cellular Homeostasis

**Figure 11:**
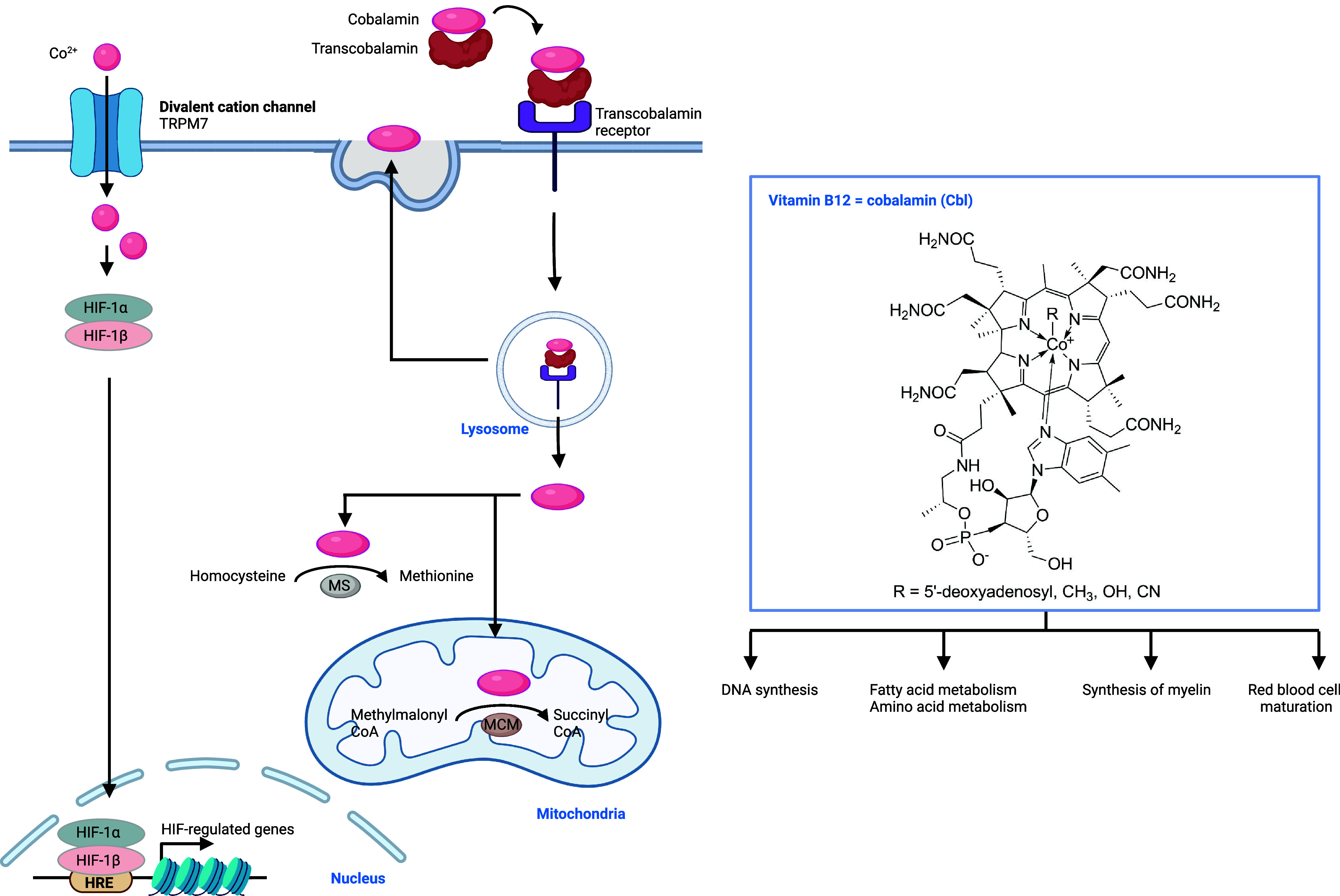
Cobalt signaling. Cobalt is taken up into the cell by a divalent cation channel and has been shown to activate the HIF-1α signaling pathway. Cobalt is a key component of vitamin B12 (cobalamin), which is necessary for the activity of enzymes implicated in DNA synthesis, fatty acid metabolism, amino acid metabolism, synthesis of myelin, and red blood cell maturation. Cobalamin binds to transcobalamin, and this complex goes to lysosomes by transcobalamin receptor-mediated endocytosis. Then, cobalamin allows for the generation of methionine in the cytosol and succinyl-CoA in mitochondria. See Abbreviations. Figure generated with BioRender.com.

**Figure 12:**
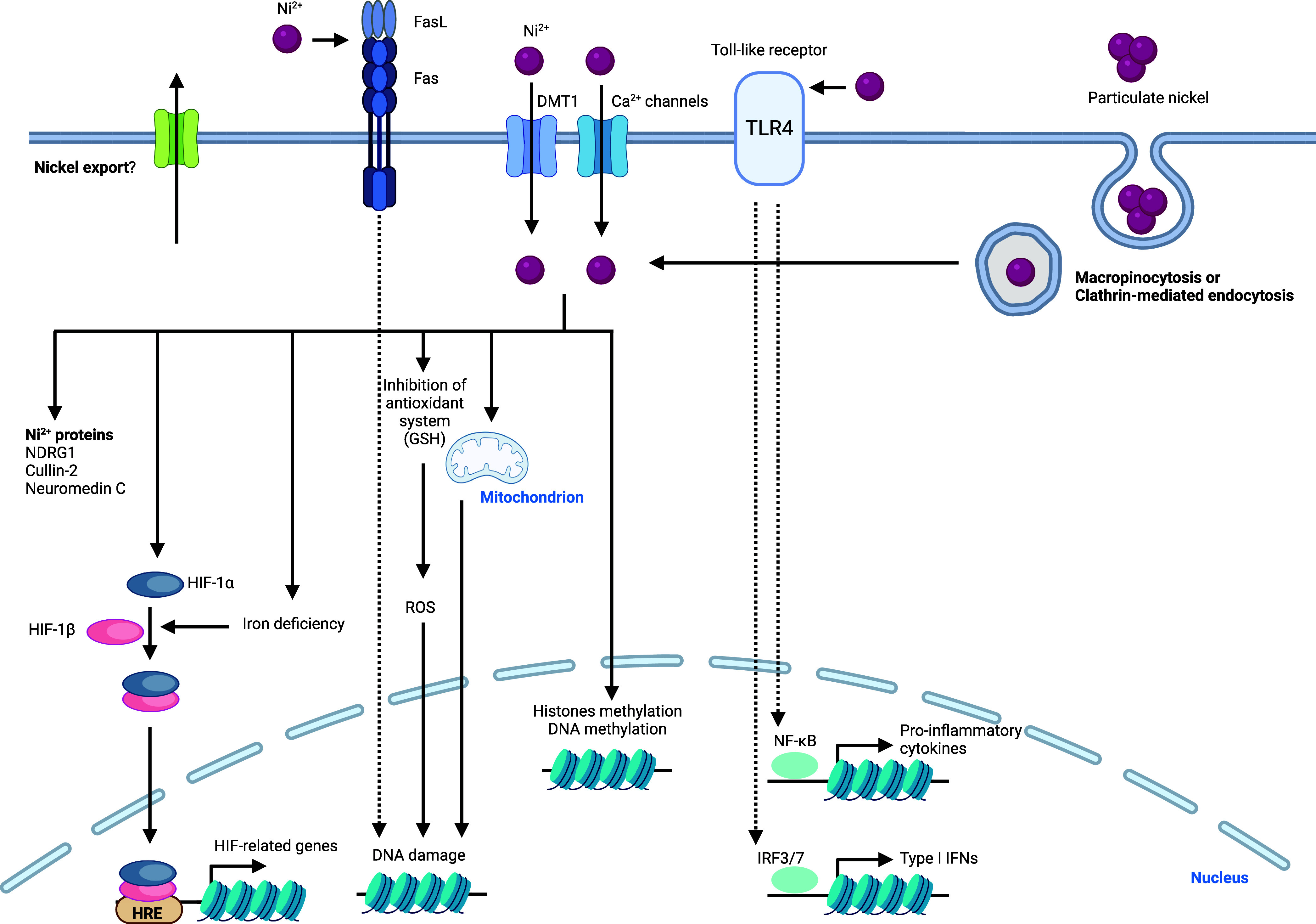
Nickel signaling. Nickel is taken up into the cell via DMT1 and calcium channels. Nickel particles are taken up by macropinocytosis or clathrin-mediated endocytosis. Nickel can induce DNA damage by inhibiting the antioxidant system (decreased activity of catalase and SOD) and by promoting mitochondrial damage (increased mitochondrial 8-hydroxyguanin). Nickel can alter gene expression by enhancing DNA and histone methylation. Nickel stabilizes the TF HIF-1α and decreases cellular iron levels; consequently, HIF-1α and HIF-1β are translocated to the nucleus, where they form a heterodimer, bind to HRE and function as a TF. Nickel is incorporated into several nickel-dependent proteins, such as NDRG1 and cullin-2. Nickel can induce Fas-mediated apoptosis. Nickel promotes inflammatory responses by activating TLR4. Dedicated nickel export mechanisms have not been reported. See Abbreviations. Figure generated with BioRender.com.

Mechanisms enabling the cellular uptake of chromium and vanadium have not been thoroughly documented. It may involve transporters previously described to regulate ion transport.^827,828^ Chromium has been reported to be taken up into the cell by Tf and TfR1-mediated endocytosis.^829^ In the cell, chromium and vanadium can coexist in various oxidation states. In bacteria, cobalt uptake, storage, and efflux have been documented in detail, but little is known in eukaryotic cells.^830^ Cobalt has been suggested to be taken up via divalent cation channels, which are not selective for a specific metal. In neurons, the same channels can mediate the uptake of cobalt, manganese, and calcium^831^ (Figure 11). Cobalt is also taken up as vitamin B12 (also known as cobalamin, a coordination complex of cobalt) via the transcobalamin receptor.^832,833^ How cobalt is exported from cells is unknown. In plants, ferroportin has been reported to play that role,^834^ suggesting that this may also take place in human cells. Nickel is found in the cells as a +2 oxidation state, and regulation of its cellular uptake has not been documented in great detail. Nickel can be internalized via DMT1 and calcium channels^835−838^ (Figure 12). In addition, artificial nickel particles can be taken up by macropinocytosis.^839^ How nickel is exported from the cell remains poorly understood. Molybdenum is taken up into the cell via ATP-dependent active transporters.^840^ Once in the cell, it can form a complex with pterin, termed molybdenum cofactor (Moco).^841,842^ Importantly, the biosynthesis of Moco requires iron, copper, and ATP. As a result, molybdenum homeostasis and its impact on cellular function are inherently linked to iron and copper metabolism.^843^

#### 4.5.2 Cellular Functions of Other d-Block Metals

**Figure 13:**
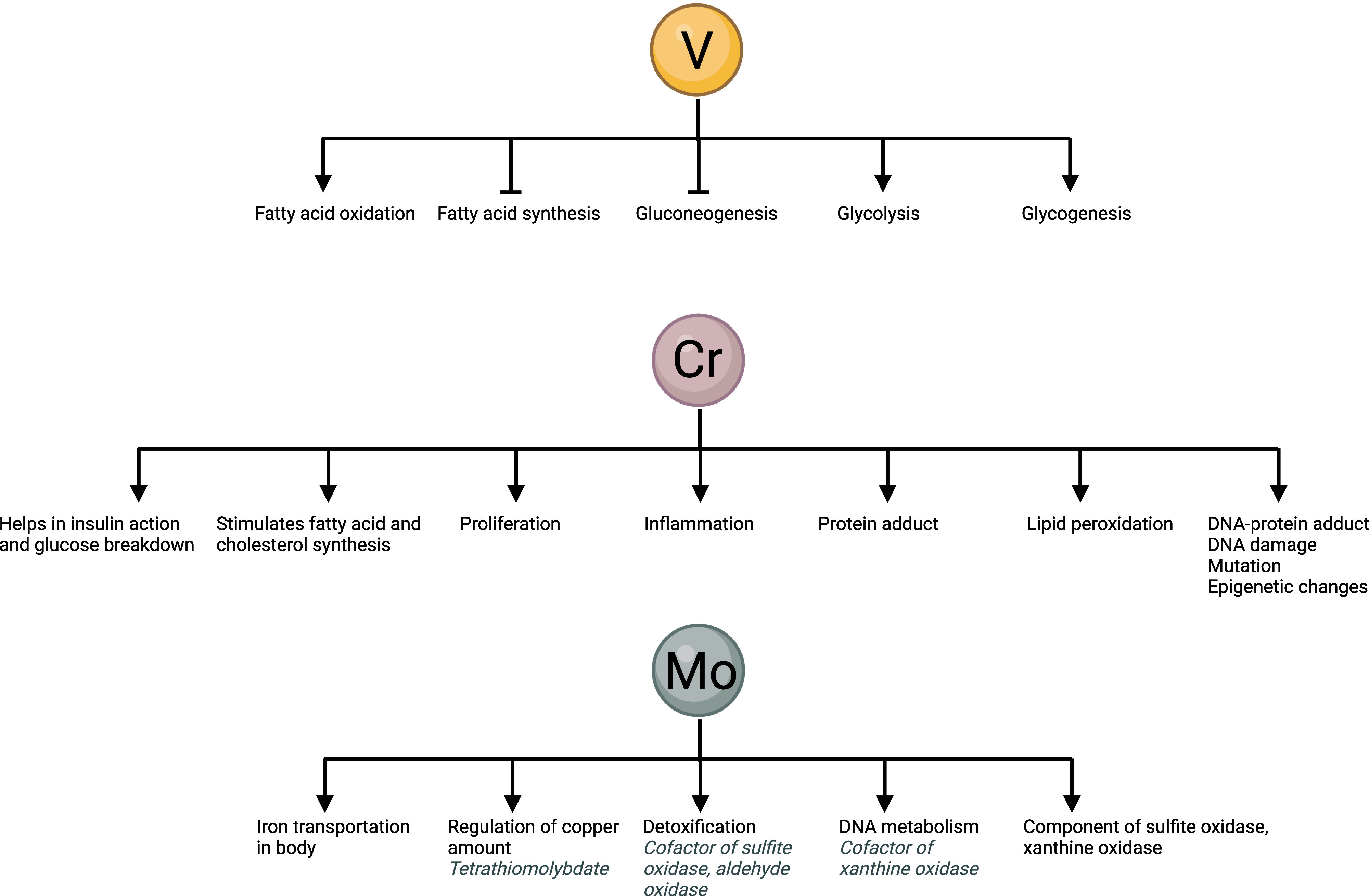
Vanadium, chromium, and molybdenum functions. Vanadium affects fatty acid metabolism, glycogen synthesis, and blood sugar levels. Chromium plays a role in carbohydrate and lipid metabolism, as well as in protein metabolism. Molybdenum impacts DNA metabolism and plays a crucial role in detoxification. Figure generated with BioRender.com.

Vanadium can affect fatty acid metabolism, glycogen biosynthesis, and blood sugar levels.^828,844^ Although vanadium-dependent enzymes have been identified in bacteria, fungi, and plants, such as haloperoxidases and nitrogenases, vanadium-dependent enzymes have not been identified in higher eukaryotes to date.^828,845^ Chromium plays a role in carbohydrate, lipid, and protein biosynthesis^846^ (Figure 13). This has been proposed to occur via chromodulin, a small polypeptide that can coordinate four chromium ions.^847^ Insulin-binding to the insulin receptor has been suggested to stimulate TfR1-mediated uptake of chromium, leading to internalization of chromium, allowing for sufficient amount of this metal to be imported such that four chromium ions can be coordinated by apochromodulin to form active holochromodulin.^848,849^ Holochromodulin then binds to the insulin receptor β subunit, which results in the activation of receptor tyrosine kinase and amplification of the insulin signal. Cobalt is part of the structure of vitamin B12, which is required for the activity of enzymes implicated in DNA biosynthesis, fatty acid metabolism, amino acid metabolism, synthesis of myelin, and maturation of red blood cells. Vitamin B12 is synthesized by some archaea and bacteria via a complex biosynthesis pathway that includes several cobalt chaperones.^850^ Cobalt is also required for the function of other noncorrin-cobalt-containing enzymes, including glucose isomerase, methylmalonyl-CoA carboxytransferase, aldehyde decarbonylase, and others.^851^ Thus, cobalt is involved in many metabolic processes. Similarly, nickel is incorporated into several nickel-dependent proteins, such as NDRG1, cullin-2, and neuromedin C. Nickel can induce DNA damage by inhibiting the antioxidant system, leading to decreased activity of catalase and SOD, and by promoting mitochondrial damage, causing an increase in mitochondrial 8-hydroxyguanin. Nickel stabilizes transcription factor HIF-1α and decreases cellular iron levels. Consequently HIF-1α and HIF-1β are translocated into the nucleus where they form a heterodimer, bind to HRE, and function as a TF.^472,473^ Since iron regulates HIF degradation,^852^ it is conceivable that nickel competes out iron from proline dioxygenases and consequently impacts HIF-1 regulation. Molybdenum-containing proteins have been identified, including mitochondrial amidoxime reductase, sulfite oxidase, xanthine oxidoreductase, and aldehyde oxidase.^841,853^ Two different types of cofactors that contain molybdenum have been reported, those containing iron and molybdenum on one hand and pterin-based molybdenum cofactors on the other hand.^843^

#### 4.5.3 Other d-Block Metal Ion Signaling and Diseases

##### 4.5.3.1 Other d-Block Metal Ion Signaling in Cancer

Vanadium has been shown to impact diabetes. Owing to the well-established increased glucose uptake that fuel cancer cell proliferation, it is conceivable that vanadium may also impact cancer.^854^ Thus, small molecules regulators of vanadium homeostasis and function may be exploited in this context.^855^ Vanadium, chromium, and nickel have also been described to be carcinogenic metals, and some evidence has emerged that these metals stimulate NF-κB signaling and cell cycle progression in cancer cells, although mechanisms at work remain poorly understood.^856^ High levels of vanadium and chromium have also been reported to increase the risk of colorectal cancer, lacking a mechanistic rationale.^857^ For example, it is not clear whether normal homeostasis and cell signaling involving these or other metals are altered upon overload with vanadium and chromium or whether this overload directly promotes undesired reactions contributing to the disease such as oxidative stress, altered mitochondrial metabolism or mutations in genomic DNA. In addition, higher levels of vanadium and chromium as well as other heavy metals have been identified in nonsmall cell lung cancer, suggesting that these metals could play a role in the etiology of the disease and/or contribute to cancer progression.^858^ Vanadium-containing compounds can inhibit several signaling pathways in cancer cells that include phosphorylation reactions in the signaling cascade, in particular MAPK signaling and PI3K/AKT signaling.^859^ Since these pathways can be upregulated in cancer cells, vanadium-containing compounds (Table 11) might find therapeutic applications. Chromium can cause DNA damage with interesting cytotoxic properties in cancer cells.^860^ This could potentially be exploited in cancer research and requires further studies. However, chromium itself has been reported to be carcinogenic, especially at higher exposures.^861^ Importantly, high concentrations of chromium and nickel in electronic nicotine delivery systems have been associated with increased lung cancer risk.^862^ Cobalt has been reported to stimulate PI3K/AKT signaling in oral squamous carcinoma cells.^863^ Nickel has been shown to have many effects on signaling in cancer cells.^864^ It can poison iron-dependent demethylases by replacing the redox-active iron catalyst, inactivating specific iron-dependent enzymes.^865^ Since these enzymes are involved in epigenetic regulation, nickel directly impacts the acquisition of distinct states of cancer cells. Nickel itself has also been shown to be carcinogenic and a driver of cancer progression.^836^

##### 4.5.3.2 Other d-Block Metal Ion Signaling in Immunity and Inflammation

Vanadium-containing drugs, so-called vanodrugs,^866^ have been shown to exhibit an effect on inflammation by affecting NF-κB and TLR signaling. Although, whether these drugs impact vanadium homeostasis and cellular functions remain unclear and explicit MoA are yet to be elucidated.^867^ Treatment of macrophages with vanadate resulted in stimulation of NF-κB and JNK signaling, suggesting a pro-inflammatory effect of vanadate and highlighting an effect on the acquisition of a pro-inflammatory cell states of macrophages.^868^ In another study, the pro-inflammatory effect of vanadate has been demonstrated in bronchoalveolar lavage cells in clinical settings.^869^ Kinases, such as cytosolic protein tyrosine kinase and others,^870^ were shown to be inhibited by vanadium and vanadium-containing compounds, in particular vanadate- and polyoxovanadate-containing compounds (Table 11), which can thus broadly affect cell signaling pathways involving phosphorylation.^871^ Because of the effect of vanadium on phosphatases, vanadate has a positive effect on T cell activation via changes in tyrosine phosphorylation.^872^ Thus, interference with signaling in immune cells by vanadium can provide control over cell states. Chromium can affect T and B lymphocyte function, macrophage function, and alter cytokine production, impacting immune responses.^873^ Chromium can inhibit the phagocytotic function of macrophages.^874^ Whether specific signaling pathways impact cell states requires further investigation. Cobalt ions can activate TLR4 generating an inflammatory response.^875^ This can be attenuated in vitro by the small molecule CLI-095, which can have an effect on soft tissue necrosis and osteolysis^875^ (Table 9). A pro-inflammatory response upon exposure to cobalt ions has also been observed in other cell types, including neutrophils, monocytes, and epithelial cells of the gut and kidney.^876^ Indeed, cobalt has been reported to induce an inflammatory state in macrophages, but underlying mechanisms driving cell state transitions require further investigation.^877^ Nickel has been shown to act as a pro-inflammatory signaling element. Interestingly, zinc can inhibit nickel uptake and attenuate nickel-induced inflammation in THP-1 cells.^835^ Moreover, nickel promotes inflammatory responses by activating TLR4. The allergic and inflammatory reaction to nickel in some individuals has been explained with differential activation of specialized CD8+ cells.^878,879^ It is conceivable that nickel contributes to signaling pathways activating these cells, which require further studies. Nickel chelation has been successfully employed to treat chronic dermatosis, including compounds such as disulfiram, EDTA, and clioquinol^880^ (Table 10).

**Table 9:**
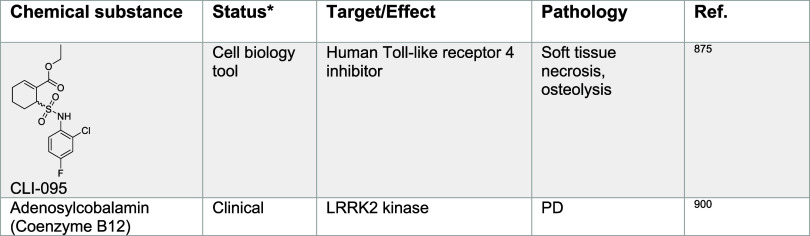
Regulators of Cobalt Signaling^875,890^

* https://www.fda.gov, https://clinicaltrials.gov

**Table 10:**
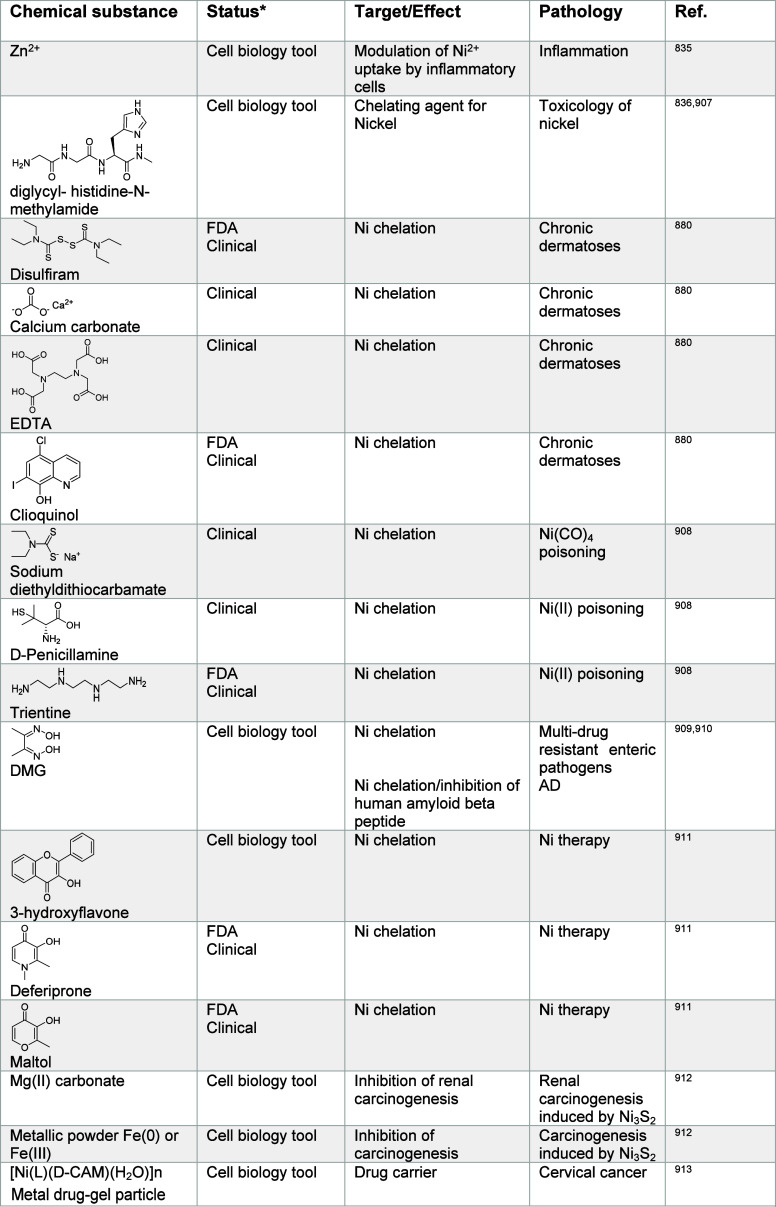
Regulators of Nickel Signaling^835,836,880,907−913^

* https://www.fda.gov, https://clinicaltrials.gov

**Table 11:**
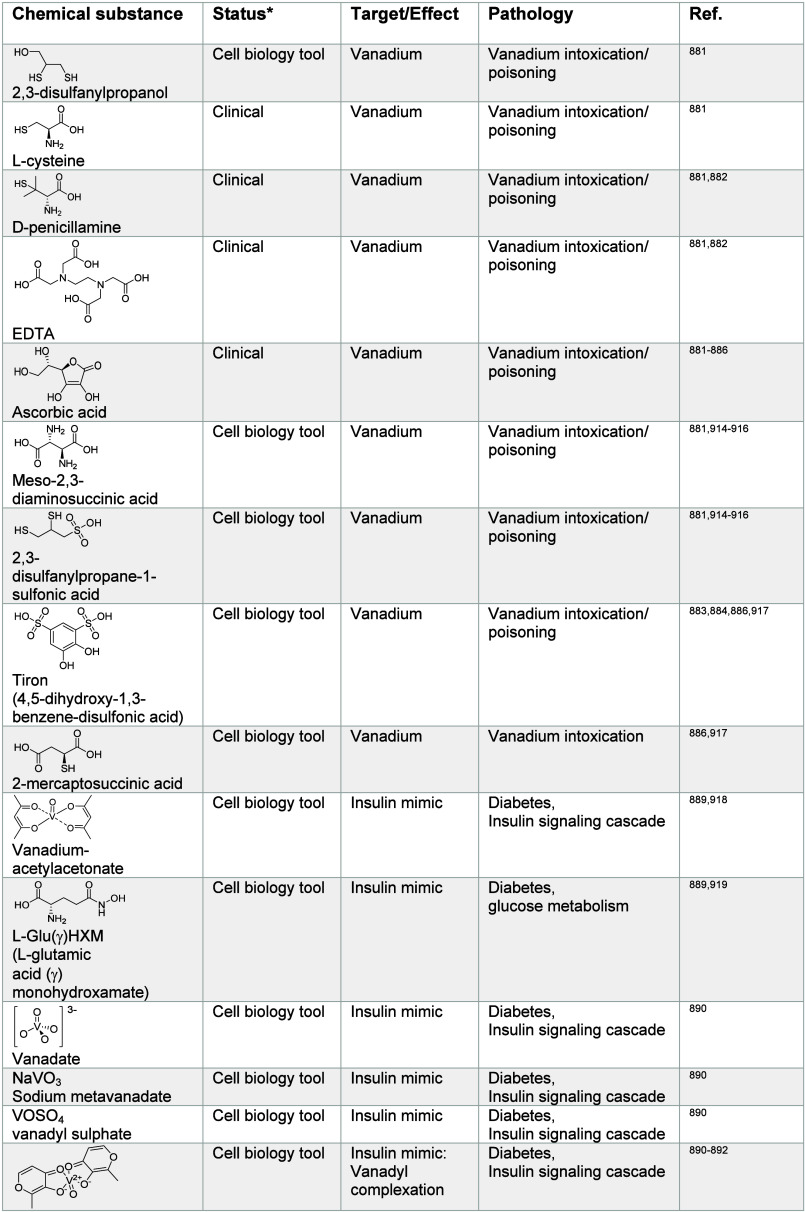

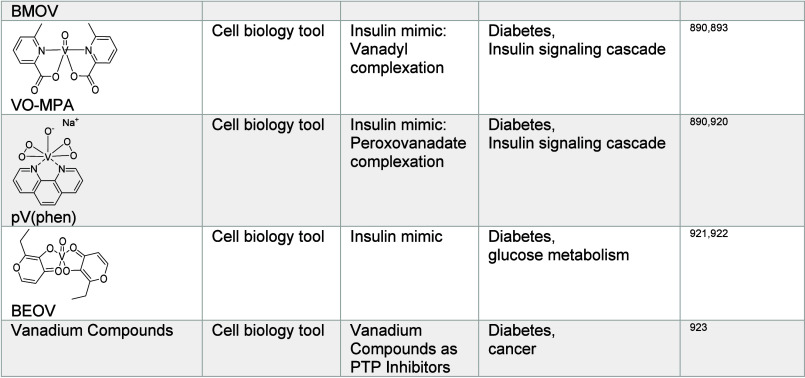
Regulators of Vanadium Signaling^881−886,890−893,914−916,917,918,919−923,^

* https://www.fda.gov, https://clinicaltrials.gov

##### 4.5.3.3 Other d-Block Metal Ion Signaling in Other Diseases

Small molecules including EDTA, 2,3-disulfanylpropanol, and the clinically approved copper chelator d-penicillamine have been shown to reduce cellular vanadium levels^881−886^ (Table 11). Vanadate can also replace phosphate in cells, and the effect of vanadium on insulin metabolism has encouraged research in this area.^887^ Vanadium-containing compounds can also mimic insulin^888,889^ and therefore exert a positive effect in diabetes. Such compounds include vanadyl sulfate (VOSO_4_), bis(maltolato)oxovanadium(IV) (BMOV), and vanadyl-methylpicolinate complex (VO-MPA) among others^890−893^ (Table 11). These effects might also be translated to other clinical settings including cardiovascular diseases.^894^ The effect of chromium on amplifying insulin signaling also represents a promising line of research for the treatment of diabetes.^895−897^ Other small molecules that affect insulin-resistance in vitro including chromium complexes have been documented^897−899^ (Table 12) and represent an interesting starting point for the development of therapeutic strategies. Mutations in leucine-rich repeat kinase 2 (LRRK2) have been associated with the progression of PD. The cobalt-containing vitamin B12 is an allosteric inhibitor of LRRK2 and has been shown to reduce neurotoxicity in rodent models of PD^900^ (Table 9). In contrast, cobalt ions have been shown to be neurotoxic^901^ and to promote PD, presumably involving the production of ROS. However, explicit mechanisms are not fully understood.^902^ Nickel has been reported to drive cardiovascular and kidney diseases and lung fibrosis.^903^ The exact mechanisms underlying these effects remain to be characterized. Deficiency of the molybdenum cofactor Moco is found in a rare genetic disease that leads to early development defects and contributes to neurodegeneration.^904^ A treatment based on precursor Z/cPMP/fosdenopterin (Table 13) has been used for therapeutic intervention.^842,905,906^

**Table 12:**
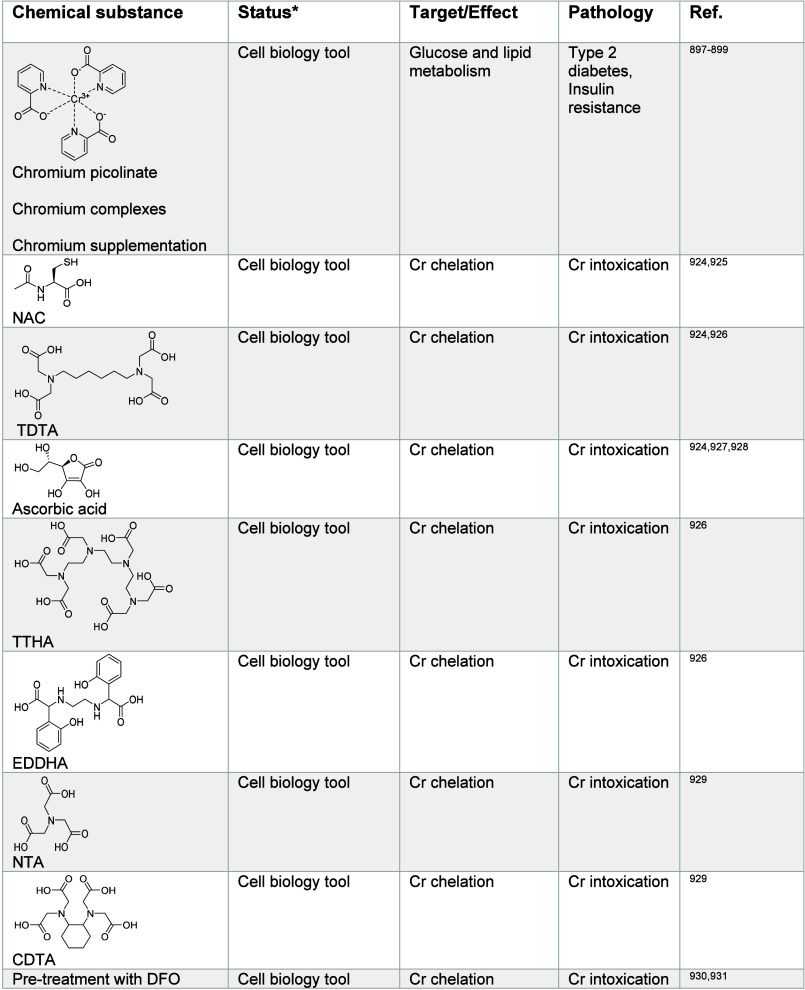
Regulators of Chromium Signaling^924−931^

* https://www.fda.gov, https://clinicaltrials.gov

**Table 13:**
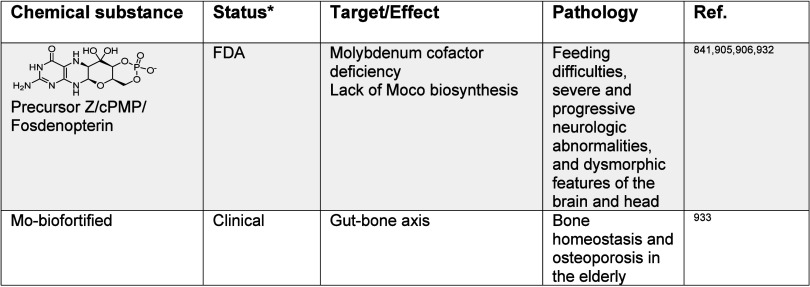
Regulators of Molybdenum Signaling^841,905,906,932,933^

* https://www.fda.gov, https://clinicaltrials.gov

## 5 Remarks

Metals exhibit a diverse range of chemistries including Lewis acid catalysis, redox, and supramolecular properties. The cell has evolved around these chemistries to mediate signal transduction while dealing with potentially detrimental reactions inherent to the reactivity of metals. This notion is best exemplified by the higher iron load required for cells to promote epigenetic reprogramming via oxidative demethylation of histone proteins, which concomitantly drives oxidation of lipids and confers vulnerability to ferroptosis.^515^ Thus, cells have developed machineries to repair oxidized membranes, enabling them to exploit iron to adapt to their environment.

Here, we have discussed the essential roles of metals in the biology of the cell. We have documented current knowledge on mechanisms regulating cellular metal homeostasis, including how metals are internalized by, distributed and stored within, and exported outside of the cell. We have described the multifaceted chemical roles of metals in transducing information inside the cell in normal physiology and how this regulation can contribute to diseases, in particular by promoting the acquisition of distinct cell properties and states. Based on their electron configuration, which defines their position in the periodic table, metals are found in the cell as crystalline forms or positively charged ions, having a propensity to act as counterions to balance charges in the cell, to promote conformational changes of biomolecules acting as cofactors that modulate function, or to mediate otherwise inefficient chemical reactions operating as catalysts.

Unlike organic biomolecules, such as proteins, nucleic acids, lipids, and glycans, metal ions are not synthesized in the cell but acquired from external sources such as nutrients. These species are smaller in size, conferring faster diffusion rates within the cell compared to biopolymers and exist in various oxidation states, being found either as free or bound species,^216^ making these chemical entities difficult to track within and outside of the cell. The literature clearly shows that multiple import and export mechanisms exist for metal ions and that these mechanisms are often not specific for a given metal. Furthermore, distinct metals can exhibit redundant properties. Thus, manipulating a given metal selectively in the cell to investigate its role in disease-relevant settings remains a challenging endeavor. Nature itself, which relies on fundamental principles of physical chemistry, has not been able to achieve complete selectivity for specific metals, which raises the question of whether we, the scientists, can invent new strategies and develop smarter chemistries to achieve selective control over metals in the cell. In other words, can we chemically target a given metal without affecting the biology of others? Can we manipulate a single function of a given metal selectively?

Our knowledge of the biology of metals is still in its infancy. For example, iron has been shown to fuel the activity of enzymes in the nucleus to promote epigenetic reprogramming and cell adaptation,^446^ yet it remains unknown how iron traffics to the nucleus where chromatin is localized. Copper(II) has been shown to catalyze NAD(H) redox cycling in mitochondria, enabling activation of immune cells in acute inflammation settings and to regulate the acquisition of a pro-metastatic state of cancer cells,^13^ but whether this is assisted by a protein stabilizing the transition state of this copper-catalyzed reaction remains unknown. Future efforts should be dedicated to identifying missing pieces in how cells control metal homeostasis. To this end, developing new probes and technologies to map out sites of action of metals in the cell, in an oxidation-state-specific manner, will be essential to our understanding of their roles in cell signaling. This may reveal new features that can be exploited for the development of small molecule modulators of cell signaling, either through direct metal targeting or alternatively acting upstream or downstream on associated signaling effectors. Another layer of complexity for the development of drugs involves our capacity to target specific tissues and specific cell types, in particular, the cells exhibiting a diseased state or more generally cells contributing to the disease or a pathological condition. How metal homeostasis is regulated at the level of specific tissues and between distinct cell types within a given tissue is complex and not fully understood, raising again the challenge of how to selectively target a metal ion signaling axis in a given cell type or tissue.

Discovery-driven approaches have been instrumental in delineating metal ion signaling pathways and the identification of related druggable targets. For example, Schreiber and Crabtree have illuminated calcium signaling partly by elucidating the MoA of complex biologically active small molecules, namely FK-506 and cyclosporin A,^22−24,26^ which has prompted interest into the development of small molecule-proximity inducers and given rise to the field of molecular glues.^934^ Notably, these compounds (Table 4) inhibit T cell activation by forming complexes with distinct immunophilins that bind to and inactivate the calcium- and calmodulin-dependent phosphatase calcineurin.^22−24,26^ These classes of compounds are potent immunosuppressors that have been used for the clinical management of organ transplants and other immune diseases. Importantly, calcium signaling is inhibited independently of direct calcium ion targeting. Similarly, studying the MoA of trichostatin A and trapoxin A (Table 8), Schreiber and co-workers have identified mammalian HDACs, contributing to the rise of epigenetics.^15^ Directly targeting zinc in the active site of HDACs using hydroxamic acid-containing small molecules has been shown to alter chromatin, impacting transcriptional programs and tumor progression. This pioneering work promoted the development of new anticancer therapeutics, demonstrating the druggable nature of this class of enzymes and raising the question of whether a given HDAC can be targeted selectively and at specific genomic loci.^826,935^ A similar rationale has been applied for the design of iron-dependent demethylase inhibitors, which can inactivate iron in the enzyme active site, with little specificity toward a given demethylase.^531,936^ Nevertheless, these studies emphasize the druggable nature of metalloenzymes targeting the metal active site. While targeting of free metal ions has been documented, notably with the use of the deferoxamine (Table 6) for the treatment of β-thalassemia and myelodysplastic syndrome, little is known about the sites of action of iron chelators in the cell and how, mechanistically, depleting iron confers therapeutic benefits with manageable off-target effects. In contrast, a small molecule screen coupled to molecular editing yielded small molecules, such as ironomycin, that directly target iron(II) in endolysosomes of DTP cancer cells and trigger ferroptosis in preclinical models of metastatic cancers.^511,514,516^ This work identified lysosomal iron as a druggable target in difficult-to-treat cancers. In another study, a phenotypic screen informed the design of the metformin dimer supformin capable of selective mitochondrial copper(II) targeting, antagonizing macrophage activation and preventing sepsis in preclinical models of acute inflammation.^13^ This work identified mitochondrial copper(II) as a driver of inflammation and a druggable target.

These studies have provided solid evidence that direct targeting of specific metals with small molecules, whose increased uptake is causal to the disease, can be achieved and confer therapeutic benefits. Hypothesis-driven approaches together with the rise of artificial intelligence and machine learning may represent an effective route forward, potentially enabling faster and more systematic production of biologically active small molecules and metal ion signaling drugs.^937,938^ It is not clear, however, whether these strategies can surpass discovery-based phenotypic approaches.^36^ Small molecules have illuminated cell signaling pathways involving metals and demonstrated that some of these processes can be targeted with therapeutic value. While no general rule can be established at this point and much remains to be learned in this area, it is fair to say that metal ion signaling regulators are undruggable until someone drugs them.

## Abbreviations

α-KG: α-ketoglutarate
Aβ: amyloid beta
ABC: ATP-binding cassette family
AD: Alzheimer’s disease
ADP: adenosine diphosphate
AKT: serine/threonine protein kinase
ALDH: aldehyde dehydrogenase
ALDH1: aldehyde dehydrogenase 1
aMDM: activated monocyte-derived macrophages
AMPAR: α-amino-3-hydroxy-5-methyl-4-isoxazolepropionic acid receptor
AMP: adenosine monophosphate
AMPK: AMP-activated protein kinase
AP-1: activating protein-1
APLP1: amyloid beta precursor like protein 1
ASIC: acid-sensing ion channel
ATOX1: antioxidant 1 copper chaperone
ATP: adenosine triphosphate
ATP13A2: ATPase cation transporting 13A2
ATP7A: copper-transporting ATPase 1
ATP7B: copper-transporting ATPase 2
Bax: Bcl-2-associated protein x
Bcl-2: B-cell lymphoma 2
BMP6: bone morphogenetic protein 6
BMPR: bone morphogenetic protein receptor
BRAF: v-raf murine sarcoma viral oncogene homologue B1
CaM: calmodulin
cAMP: cyclic adenosine monophosphate
CaSR: calcium-sensing receptor
Cas: CRISPR associated protein
Cav3.1/3.2/3.3: T-type voltage-gated calcium channel 3.1/3.2/3.3
CAX: cation/proton exchanger
Cbl: cobalamin
CCS: copper chaperone for superoxide dismutase
CD44: cluster of differentiation 44
CDC25C: cell division cycle 25C
Cdk1: cyclin-dependent kinase 1
cGAS: protein cyclic GMP-AMP synthase
CK2: casein kinase 2
CLDN16/19: claudin-16/19
CNNM3/4: cyclin and CBS domain divalent metal cation transport mediator 3/4
CoA: coenzyme A
COX: cytochrome c oxidase
CRAC: calcium release-activated calcium channel
CREB: cyclic-AMP responsive element-binding protein
CRISPR: clustered regularly interspaced short palindromic repeats
CSCs: cancer stem cells
c-src: cellular src
Ctr1/2: copper transporter 1/2
Cyt c: cytochrome c
DAG: diacylglycerol
DAT1: dopamine transporter 1
DC_AC50: 3-amino-*N*-(2-bromo-4,6-difluorophenyl)-6,7-dihydro-5*H*-cyclopenta[*b*]thieno[3,2-*e*]pyridine-2-carboxamide
DFO: deferoxamine
DFX: deferasirox
DHBA: dihydroxybenzoic acid
DLAT: dihydrolipoyl acetyltransferase
DLST: dihydrolipoyl succinyltransferase
DMT1: divalent metal transporter 1
DNA: deoxyribonucleic acid
dsDNA: double-stranded DNA
DTP: drug-tolerant persister
DTPA: diethylenetriaminepentaacetic acid
ECM: extracellular matrix
EGFR: epithelial growth factor receptor
EMT: epithelial-to-mesenchymal transition
ENaC: epithelial sodium channel
ER: endoplasmic reticulum
ERFE: erythroferrone
ERK: extracellular-signal-regulated kinase
ERK1/2: extracellular-signal-regulated kinase 1/2
ERMES: endoplasmic reticulum mitochondria encounter structure
ESCC: esophageal squamous cell carcinoma
ETC: electron transport chain
FasL: Fas ligand
FHA: forkhead-associated domain
FIH1: factor inhibiting HIF-1
FSP1: ferroptosis suppressor protein 1
FTH1: ferritin heavy chain
FTL: ferritin light chain
FTO: fat mass and obesity-associated protein
G4: G-quadruplex
GABA: γ-aminobutyric acid
GABA_A_: γ-aminobutyric acid type A
GLUTs: glucose transporters
GPCR: G protein-coupled receptor
GPX4: glutathione peroxidase 4
GSH: glutathione
GSK-3: glycogen synthase kinase 3
GSSG: glutathione disulfide
H3K9me2: histone 3 lysine 9 dimethyl
H3K27me3: histone 3 lysine 27 trimethyl
HAMP: hepcidin antimicrobial peptide
HAT: histone acetyltransferase
HBED: *N*,*N*′-di(2-hydroxybenzyl)ethylenediamine-*N*,*N*′-diacetic acid
HCC: hepatocellular carcinoma
HCN: hyperpolarization-activated cyclic nucleotide-gated channel
HD: Huntington’s disease
HDAC: histone deacetylase
HIF: hypoxia-inducible transcription factor
HIF-1: hypoxia-inducible transcription factor-1
HIF-1α/1β/2α: hypoxia-inducible transcription factor-1alpha/1beta/2alpha
HIV-1: human immunodeficiency virus type 1
HN1L: hematological and neurological expressed 1-like protein
HRE: hypoxia-response element
ICP-MS: inductively coupled plasma-mass spectrometry
IFN: interferon
IFN-λ3/γ: interferon lambda3/gamma
IFNLR1: interferon lambda receptor 1
IKK: IκB kinase
IKKβ: IκB kinase β
IL-1β/1–4/2/17: interleukin-1β/1–4/2/17
IP_3_: inositol triphosphate
IP_3_R: IP3 receptor
IRE: iron-responsive element
IRF3/7: interferon regulatory factor 3/7
IRP: iron regulatory protein
IRP1/2: iron regulatory protein 1/2
JAK: Janus kinase
JNK: c-Jun N-terminal kinase
K.Ca3.1: calcium-activated potassium channel 3.1
KDM3B/6A/6B: Histone lysine demethylase 3B/6A/6B
KHE: potassium/proton exchanger
Kir: inward-rectifier potassium channel
K-ras: Kirsten rat sarcoma virus
Kv: potassium voltage-gated channel
LCC-12: lipophilic copper clamp 12
LCN2: lipocalin-2
LCN2R: lipocalin-2 receptor
LCS-1: lung cancer screen 1
LFA-1: lymphocyte function-associated antigen 1
LRRK2: leucine-rich repeat kinase 2
LTCC: L-type calcium channel
MagT1: magnesium transporter 1
MAPK: mitogen-activated protein kinase
MCM: methyl malonyl CoA mutase
MCOLN1: mucolipin-1
MCU: mitochondrial calcium uniporter
MD: Menkes disease
MEK: mitogen-activated protein kinase kinase
MEK1/2: mitogen-activated protein kinase kinase 1/2
METAP1: methionine aminopeptidase 1
MICU1: mitochondrial calcium uptake 1
mitoBK_Ca_: mitochondrial large-conductance calcium-regulated potassium channel
mitoK_ATP_: mitochondrial ATP-regulated potassium channel
mitoKv: mitochondrial potassium voltage-gated channel
ML-9: 1-(5-chloronaphthalen-1-yl)sulfonyl-1,4-diazepane
ML-SA1: 2-[2-oxo-2-(2,2,4-trimethyl-3,4-dihydroquinolin-1-yl)ethyl]isoindole-1,3-dione
MMP-9: matrix metalloproteinase 9
MoA: mechanism of action
Moco: molybdenum cofactor
MRE: metal response element
mRNA: messenger RNA
MRS-1844: *N*-methylnitrendipine
MRS-1845: *N*-propargylnitrendipine
MRS2: mitochondrial RNA splicing 2
MS: methionine synthase
MT: metallothionein
MT1/2/3: metallothionein 1/2/3
MTF-1: metal response element transcription factor-1
mTOR: mammalian target of rapamycin
mTORC1: mammalian target of rapamycin complex 1
MVBs: multivesicular bodies
NAADP: nicotinic acid adenine dinucleotide phosphate
NAD+: nicotinamide adenine dinucleotide
NADH: nicotinamide adenine dinucleotide hydride
NALCN: sodium leak channel non-selective
naMDM: non activated monocyte-derived macrophages
NASH: non alcoholic steatohepatitis
NCBn1: sodium/bicarbonate cotransporter
NCKX: potassium-dependent sodium/calcium exchanger
NCLX: mitochondrial sodium/calcium exchanger
NCOA4: nuclear receptor coactivator 4
NCX: sodium/calcium exchanger
NDRG1: N-myc downstream regulated 1
NF-κB: nuclear factor kappa-light-chain-enhancer of activated B cells
NFAT: nuclear factor of activated T cells
NFAT5: nuclear factor of activated T cells 5
NHE: sodium/proton exchanger
NIPA1–4: nonimprinted in Prader–Willi/Angelman syndrome 1–4
NIS: sodium/iodide symporter
NKCC: sodium/potassium/chloride cotransporter
NLRP3: nucleotide-binding domain, leucine-rich-containing family, pyrin domain-containing-3
NMDAR: *N*-methyl-d-aspartate receptor
NRAMP: natural resistance-associated macrophage protein 1
NRF2: nuclear factor erythroid 2-related factor 2
NSCLC: non small cell lung cancer
ORAI: calcium release-activated calcium modulator
OSCC: oral squamous cell carcinoma
P2X4R: purinergic P2X receptor 4
P2X7R: purinergic P2X receptor 7
PARK7: Parkinson disease protein 7
PCBP1/2: poly(rC)-binding protein 1/2
PD: Parkinson’s disease
PDE3B: phosphodiesterase 3B
PDZD11: PDZ domain-containing protein 11
PHD: iron-dependent prolyl hydroxylase
PHF8: plant homeodomain (PHD) finger protein 8
Pi: inorganic phosphate
PI3K: phosphatidylinositol-3 kinase
pIRF3: phosphorylated interferon regulatory factor 3
Pit1: inorganic phosphate transporter 1
PKC: protein kinase C
PLC: phospholipase C
PLCγ: phospholipase C gamma
PLEKHA: Pleckstrin homology domain containing family A
PLEKHA 5/6/7: Pleckstrin homology domain containing family A 5/6/7
PMCA: plasma membrane calcium ATPase
pNFAT: phosphorylated nuclear factor of activated T cells
pNF-κB: phosphorylated nuclear factor kappa-light-chain-enhancer of activated B cells
PTP: protein tyrosine phosphatases
PTP1b: protein-tyrosine phosphatase 1b
pVHL: Von Hippel–Lindau tumor suppressor
Ras: rat sarcoma virus
RNA: ribonucleic acid
ROS: reactive oxygen species
RyR: ryanodine receptor
Sco1/2: cytochrome *c* oxidase assembly protein 1/2
SERCA: sarcoplasmic/endoplasmic reticulum calcium ATPase
SGLT: sodium/glucose cotransporter
SLC24A5: solute carrier family 24 member 5
SLC25A3: solute carrier family 25 member 3
SLC25A5: solute carrier family 25 member 5
SLC30A10: solute carrier family 30 member 10
SLC39A14: solute carrier family 39 member 14
SLC41A1: solute carrier family 41 member 1
SLC41A2: solute carrier family 41 member 2
SLC41A3: solute carrier family 41 member 3
SLC46A3: solute carrier family 46 member 3
SMAD: suppressor of mothers against decapentaplegic
SMAD1/5/8: mothers against decapentaplegic homologue 1/5/8
SOD: superoxide dismutase
SOD1/2: superoxide dismutase 1/2
SPCA: secretory pathway calcium ATPase
SPCA1/2: secretory pathway calcium-ATPase pump type 1/2
SR: sarcoplasmic reticulum
STAT: signal transducer and activator of transcription
STAT3: signal transducer and activator of transcription 3
STEAP: 6-transmembrane epithelial antigen of prostate
STEAP4: 6-transmembrane epithelial antigen of prostate 4
STIM: stromal interaction molecule
STING: stimulator of interferon genes
TEPA: tetraethylenepentamine
TET: ten-11 translocation
Tf: transferrin
TfR1: transferrin receptor
TF: transcription factor
TGF: transforming growth factor
TGF-β: transforming growth factor beta
TIMP1: tissue inhibitor of metalloprotease-1
TLR: Toll-like receptor
TLR4: Toll-like receptor 4
TMEM94/165/175: transmembrane protein 94/165/175
TMPRSS6: transmembrane serine protease 6
TNBC: triple-negative breast cancer
TPC: two-pore channel
TRP: transient receptor potential
TRPA1: transient receptor potential ankyrin 1
TRPC: transient receptor potential canonical
TRPC1/3–6: transient receptor potential canonical 1/3–6
TRPM2/6/7/8: transient receptor potential melastatin 2/6/7/8
TRPML: transient receptor potential mucolipin
TRPML1: transient receptor potential mucolipin 1
TRPP2: transient receptor potential polycystin-2
TRPV1/2/3/4/6: transient receptor potential vanilloid type 1/2/3/4/6
TTHA: triethylenetetramine hexaacetic acid
TTM: tetrathiomolybdate
ULK1/2: UNC51-like kinase-1/2
UTRs: untranslated regions
VDAC: voltage-dependent anion channel
VEGFR: vascular endothelial growth factor receptor
VGSC: voltage-gated sodium channel
WD: Wilson’s disease
Wnt: wingless/integrated
ZIP: ZRT/IRT-like protein
ZIP1–11/13/14/16: ZRT/IRT-like protein 1–11/13/14/16
ZNFs: zinc-finger proteins
ZNG1: zinc-regulated GTPase metalloprotein activator 1
ZnT: zinc transporter
ZnT1/2/4/5/6/7/9/10: zinc transporter 1/2/4/5/6/7/9/10
